# Effective genome editing and identification of a regiospecific gallic acid 4-*O*-glycosyltransferase in pomegranate (*Punica granatum* L.)

**DOI:** 10.1038/s41438-019-0206-7

**Published:** 2019-11-08

**Authors:** Lijing Chang, Sheng Wu, Li Tian

**Affiliations:** 1 Shanghai Key Laboratory of Plant Functional Genomics and Resources, Shanghai Chenshan Botanical Garden, 201602 Shanghai, China; 2 Shanghai Chenshan Plant Science Research Center, Chinese Academy of Sciences, 201602 Shanghai, China; 3 Department of Plant Sciences, University of California, Davis, CA 95616 USA

**Keywords:** Molecular engineering in plants, Secondary metabolism, Plant molecular biology, Mutagenesis

## Abstract

Pomegranate (*Punica granatum* L.) trees are woody perennials that bear colorful and nutritious fruits rich in phenolic metabolites, e.g., hydrolyzable tannins (HTs) and flavonoids. We here report genome editing and gene discovery in pomegranate hairy roots using Clustered Regularly Interspaced Short Palindromic Repeats (CRISPR)/CRISPR-associated protein 9 (Cas9) (CRISPR/Cas9), coupled with transcriptome and biochemical analyses. Single guide RNAs (sgRNAs) were designed to target two UDP-dependent glycosyltransferases (UGTs), *Pg*UGT84A23 and *Pg*UGT84A24, which possess overlapping activities in β-glucogallin (a galloylglucose ester; biosynthetic precursor of HTs) biosynthesis. A unique accumulation of gallic acid 3-*O*- and 4-*O*-glucosides (galloylglucose ethers) was observed in the *PgUGT84A23* and *PgUGT84A24* dual CRISPR/Cas9-edited lines (i.e., *ugt84a23 ugt84a24*) but not the control (empty vector) or *PgUGT84A23/PgUGT84A24* single edited lines (*ugt84a23* or *ugt84a24*). Transcriptome and real-time qPCR analyses identified 11 UGTs with increased expression in the *ugt84a23 ugt84a24* hairy roots compared to the controls. Of the 11 candidate UGTs, only *Pg*UGT72BD1 used gallic acid as substrate and produced a regiospecific product gallic acid 4-*O*-glucoside. This work demonstrates that the CRISPR/Cas9 method can facilitate functional genomics studies in pomegranate and shows promise for capitalizing on the metabolic potential of pomegranate for germplasm improvement.

## Introduction

The woody plant pomegranate (*Punica granatum* L.) produces colorful flowers and fruits with ornamental and culinary values. Different pomegranate tissues have historically been used for alleviating symptoms or treating various diseases due to the accumulation of a wide diversity of bioactive metabolites^1^. In recent years, pomegranate fruits and juice have been pursued by consumers for their favorable nutritional quality, contributed by the abundant phenolic compounds, e.g., hydrolyzable tannins (HTs) and flavonoids, in these tissues and products^2^. Genetic variations underlying different metabolite profiles reportedly exist in pomegranate and have been utilized for breeding cultivars with desirable traits^3^. Complementary to the classic breeding approach, new molecular techniques, such as genome editing, can enable targeted modification of key metabolic genes for improved nutritional and commercial quality of pomegranate fruits and products.

Among the various genome-editing technologies, Clustered Regularly Interspaced Short Palindromic Repeats (CRISPR)/CRISPR-associated protein 9 (Cas9) (CRISPR/Cas9) has gained increasing popularity for its efficiency and ease of use. In this method, a single guide RNA (sgRNA) directs the Cas9 nuclease to the target gene sequence upstream of a protospacer adjacent motif. Cas9 creates a break in the double-strand DNA, which is then ligated by homology-directed repair or non-homologous end joining^4^. In general, five genotypes can be obtained from the CRISPR/Cas9-mediated genome editing in a diploid species, including wild type (no mutations), homozygous mutant (same mutations in both alleles), heterozygous mutant/monoallelic (mutation in one allele, wild type in the other allele), biallelic (different mutations in the two alleles), and chimera (more than two different mutations in the alleles)^5^. Initially used for disruption of gene function, there has been rapid advancement in the CRISPR/Cas9 technology for more precise (e.g., base editing) and versatile (e.g., controlling gene expression) genome editing^6,7^.

Although CRISPR/Cas9 has been successfully adopted in many plant species (e.g., Arabidopsis, tobacco, tomato, rice etc.), its application has not been reported in pomegranate^8^. In consideration of the time and effort required for transformation and regeneration of pomegranate plants, we chose a hairy root system for testing the feasibility and efficacy of CRISPR/Cas9-mediated genome editing in pomegranate. This is because hairy roots can be induced from different pomegranate explants, accumulate HTs and other phenolic compounds, are transformable, and produce sufficient amounts of tissues for molecular and metabolite analyses within 3 months of transformation^9^.

**Fig. 1:**
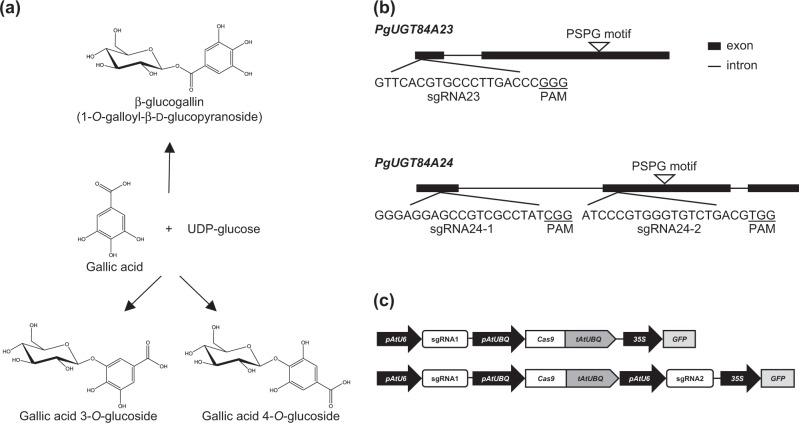
CRISPR/Cas9 editing of *PgUGT84A23* and *PgUGT84A24.* **a** Gallic acid and UDP-glucose are converted to galloylated glucose with an ester (β-glucogallin) or ether (gallic acid 3-*O-* or 4-*O*-glucoside) linkage by UGT activities. **b** The schematic representation of sgRNAs targeting *PgUGT84A23* and *PgUGT84A24*. The protospacer adjacent motif (PAM) sequences are underlined. The location of the Plant Secondary Product Glycosyltransferase (PSPG) motif is indicated. **c** The modified expression plasmids that include the psgR-Cas9-At or p2×sgR-Cas9-At cassettes and a green fluorescent protein (GFP) selection marker.

To select an easily discernable phenotype for verification of successful genome editing in pomegranate hairy roots, we chose *PgUGT84A23* and *PgUGT84A24*, encoding two UDP-dependent glycosyltransferases (UGTs) that form β-glucogallin from gallic acid and UDP-glucose, as target genes (Fig. 1a)^10^. Reduced accumulation of punicalagin α and β isomers (the most abundant HTs in pomegranate; produced from β-glucogallin) was observed in pomegranate hairy roots with attenuated *Pg*UGT84A23 and *Pg*UGT84A24 activities (via RNAi suppression of *PgUGT84A23* and *PgUGT84A24* gene expression)^10^. Therefore, the punicalagin levels in hairy roots can serve as a metabolic phenotype for knocking out *Pg*UGT84A23 and *Pg*UGT84A24 activities through genome editing.

In this work, we generated pomegranate hairy root lines containing CRISPR/Cas9-edited *PgUGT84A23* and/or *PgUGT84A24*. We also modified the expression plasmids by incorporating a green fluorescent protein (GFP) marker for rapid and non-destructive screening of transgenic hairy roots. Metabolite analysis was conducted on the control (empty vector) as well as the single and dual CRISPR/Cas9-edited hairy roots (i.e., *ugt84a23*, *ugt84a24*, and *ugt84a23 ugt84a24*) and showed significant changes in *ugt84a23 ugt84a24*. Comparative transcriptome analysis was subsequently carried out on the control and *ugt84a23 ugt84a24* hairy roots, which led to the identification of a new regioselective UGT toward gallic acid.

## Results

### The CRISPR/Cas9-sgRNAs effectively created mutations in *PgUGT84A23* and *PgUGT84A24*

To knockout the activity of *Pg*UGT84A23 or *Pg*UGT84A24, one sgRNA for *PgUGT84A23* (sgRNA23) and two sgRNAs for *PgUGT84A24* (sgRNA24-1 and sgRNA24-2) were designed, which are specific for each target gene and away from the Plant Secondary Product Glycosyltransferase (PSPG) motif conserved among plant UGTs for binding sugar donors (Fig. 1b). To eliminate both *Pg*UGT84A23 and *Pg*UGT84A24 activities, sgRNA23 and sgRNA24-1/sgRNA24-2 were placed into the same expression plasmid (Fig. 1c). A GFP selection marker was incorporated in the plasmid for sgRNA and Cas9 expression and used for screening of transgenic hairy roots (Fig. 1c).

Two hundred pomegranate hairy roots were transformed with each sgRNA or sgRNA combinations (i.e., sgRNA23, sgRNA24-1, sgRNA24-2, sgRNA23+sgRNA24-1, or sgRNA23+sgRNA24-2) and about 80% of the transformants exhibited green fluorescence emission upon excitation. Multiple GFP-positive hairy roots derived from each expression plasmid were randomly selected for sequencing (Tables 1 and 2). Of the seven sgRNA23 hairy root lines analyzed, there were two homozygous mutants with a 1-bp deletion or a 74-bp deletion and five biallelic mutants carrying deletions (or a deletion and a mismatch) of different sizes (Table 1). For sgRNA24-1, one homozygous mutant of a 1-bp deletion, one heterozygous mutant with a 3-bp mismatch and a 13-bp deletion in one allele, and six biallelic mutants were detected (Table 1). For sgRNA24-2, one homozygous mutant of a combined 5-bp deletion and 1-bp mismatch and eight biallelic mutants with deletions and/or insertions were identified (Table 1).

**Table 1:** Mutations identified in the pomegranate *ugt84a23* or *ugt84a24* hairy root lines.

sgRNA	Mutations in the target gene alleles
23 (85)	Homozygous 1-bp deletion (T)
23 (129)	4-bp deletion (GTCA), [5-bp deletion and 1-bp mismatch (CCGGGCGCACG in mutant vs. CCGGGTCAAGGGCACG in wild type)]
23 (130)	1-bp deletion (T), 5-bp deletion (GTCAA)
23 (241)	Homozygous 74-bp deletion (CATGTCTTCTTGGTCTCCTTCCCGGGTCAAGGGCACGTGAACCCACTGCTGAGGCTCGGGAAGAGGCTCGCCTC)
23 (244)	3-bp deletion (TCA), 52-bp deletion (TATGGGTTCGGAGTCGTCACTTGTCCATGTCTTCTTGGTCTCCTTCCCGGGT)
23 (249)	6-bp deletion (CCCGGG), 74-bp deletion (CATGTCTTCTTGGTCTCCTTCCCGGGTCAAGGGCACGTGAACCCACTGCTGAGGCTCGGGAAGAGGCTCGCCTC)
23 (252)	1-bp deletion (T), 10-bp deletion (CCTTCCCGGG)
24-1 (76B)	WT, [3-bp mismatch and 13-bp deletion (CGCCAGGATTA in mutant vs. CGCCTATCGGGGACGGGTTCATTA in wild type)]
24-1 (77B)	1-bp deletion (T), 1-bp deletion (C)
24-1 (78B)	Homozygous 1-bp deletion (C)
24-1 (86)	1-bp deletion (T), 7-bp deletion (TCGCCTA)
24-1 (140)	1-bp deletion (C), 7-bp deletion (TCGCCTA)
24-1 (208)	1-bp deletion (C), 5-bp deletion (GCCTA)
24-1 (214)	1-bp insertion (T), 4-bp deletion (CGCC)
24-1 (251)	1-bp insertion, [5-bp deletion and 1-bp mismatch (GCCGAATCGG in mutant and GCCGTCGCCTATCGG in wild type)]
24-2 (79)	1-bp deletion (G), 2-bp deletion (TG)
24-2 (80)	1-bp insertion (A), 1-bp insertion (T)
24-2 (81)	1-bp insertion (A), 40-bp deletion (GACGTGGCCGAGAGTCTCGGTCTACCCTCGGCCATGCTCT)
24-2 (87)	46-bp deletion (GTTCATCCCGTGGGTGTCTGACGTGGCCGAGAGTCTCGGTCTACCC), 48-bp deletion (GTGTCTGACGTGGCCGAGAGTCTCGGTCTACCCTCGGCCATGCTCTGG)
24-2 (88)	1-bp insertion (A), 6-bp deletion (GTGTCT)
24-2 (89)	1-bp deletion (G), 2-bp deletion (TG)
24-2 (118)	1-bp insertion (A), 1-bp insertion (C)
24-2 (143)	Homozygous [5-bp deletion and 1-bp mismatch (TCCCGTGGGCACGT in mutant vs. TCCCGTGGGTGTCTGACGT in wild type)]
24-2 (240)	1-bp deletion (A), 2-bp deletion (TG)

sgRNA23 (23) targets *PgUGT84A23*, whereas sgRNA24-1 (24-1) and sgRNA24-2 (24-2) target different regions of *PgUGT84A24*. Specific hairy root line numbers are shown in parenthesis next to the sgRNA designation. For mutations that include a deletion and a mismatch, both the mutant and the reference wild-type sequences are shown

WT, wild-type allele

**Table 2:** Mutations identified in the pomegranate *ugt84a23 ugt84a24* hairy root lines.

sgRNA	Mutations in the target gene alleles	
	*PgUGT84A23*	*PgUGT84A24*
23+24-1 (305)	WT, 6-bp deletion (GGCACG), 7-bp deletion (CCGGGTC), 13-bp insertion (TGAATTAGTTAGT)	1-bp deletion (C), 30-bp deletion (CGTCGCCTATCGGGGACGGGTTCATTAGGT), 1162-bp deletion (CCTCGATCAGTACTTGCCCCAGCTCGAGAAAGTCGGCAAGGAGGTACATATAACTATATATATTATAAATCAGTTAGGAGTTAATTAATTATATGAGTTCTTCGTAATTTTTTTCTCCTGGAATTTTAATTAAAAAAAAGTTAGGAGTTGCTAAATTATTTGGATGCAGACAGACTTCAAATTATGAAAACGGTTTTGGTTTGGTCCATATAACAAAATCATTTATAGTACGCAATTCTACTCTACACATTTTTCAGCTTATACCCCATTAATTGGAGAGTAATTTCTTCATATTCCTACATTCAGGTTGGCAAAATTTTTAATATACCTTTTCGGTTATAAAATTTTTAATATACCTTAGCCGAGGACAACTATATTCATCTTAAACTACCCAAAATATATGCCGATTCAAATGCAGGAATTTGAAGGTATAATATTAAATACTTGGTCCAGACAAGCCATAATAATTAAATAAGGAATCTATATATGGTTTGCAATATTAGGACATAAATAATAAAACATGCCATCATTTTACAGTATTAACTTTTTCTAGAGCACGTATAGTTTTTCAACTTTTTCCTTTTGGGTGAACAGAGCACAATATAGTTATGATAATACATTGAAAACTAAGTCTAAAATACGAGAAATGATTTGGTAATATTTTTTTTTGTGCAAACACTTGTATTCGGAAGCCTAATTGGATCTTAACTAATTCAGTTGAACCGAGTCGATTCACTAAGGGGGTAAAACTCTCATAATAATATTACCTTTATCATGTTATGTGAGAAAACACTAATTAGATTACAACAGGTAAATGAATGCTAATCGTTCGAATAAATATCATTTGTTCTTAATTAAGGTAATATTATTGATTTTTCCTCGTCAGATCATTGTTGGGGAAGTACAATCCTCACCTATAACTTATTATTTTCCAGAGTTGGTGACAAAATTACGACATTGAAATTGATGAAGGGAATACGATATTGATTTTGTCTTTCGATTCATTCACAAATGAAGTGCGATCAATAATGTCTATCACGGACACTGCAGGTAATTCCACGGATGATAAAGAAGAACGAGGAGCAGAACCGTCCCGTGTCCTGCCTCATCAACAACCCGTTCATCCCGTGGGTGTCTGACGTGGCCGAGAGTCTCGGTCTAC)
23+24-1 (307)	Homozygous 2-bp deletion (TC)	3-bp deletion (GCC), 19-bp deletion (CGGGGAGGAGCCGTCGCCT)
23+24-1 (310)	Homozygous 10-bp deletion (GTCAAGGGCA)	Homozygous 1-bp deletion (C)
23+24-1 (316)	Homozygous 1-bp deletion (G)	1-bp deletion (C), 3-bp deletion (CGC)
23+24-1 (318)	1-bp mismatch (G/T), 3-bp deletion (TCA), 9-bp deletion (GTCAAGGGC), 20-bp deletion (CTTGGTCTCCTTCCCGGGTC), 25-bp deletion (TTCTTGGTCTCCTTCCCGGGTCAAG)	WT, 18-bp deletion (GAGCCGTCGCCTATCGGG)
23+24-1 (319)	Homozygous 1-bp deletion (T)	1-bp deletion (T), 29-bp deletion (CGTCGCCTATCGGGGACGGGTTCATTAGG)
23+24-1 (321)	WT, [21-bp mismatch and 6-bp deletion (GAAGGGTTCACCCGACCACAC in mutant vs. TTCCCGGGTCAAGGGCACGTGAACCCA in wild type)]	1-bp deletion (T), 8-bp deletion (CGTCGCCT)
23+24-1 (322)	Homozygous 2-bp insertion (GC)	6-bp deletion (GTCGCC), 7-bp insertion (TCCTTTT)
23+24-1 (323)	Homozygous 1-bp deletion (G)	3-bp deletion (CGC), 21-bp deletion (GAGGAGCCGTCGCCTATCGGG)
**23+24-1 (324)**	1-bp deletion (G), 3-bp deletion (GGG)	1-bp deletion (T), 6-bp deletion (TCGCCT)
23+24-1 (325)	3-bp deletion (TCA), 4-bp deletion (TCAA)	6-bp deletion (GCCGTC), 7-bp deletion (TCGCCTA)
**23+24-1 (327)**	1-bp insertion (T), 5-bp deletion (GGGTC)	1-bp insertion (A), 3-bp deletion (CGC)
**23+24-1 (346)**	4-bp deletion (TCAA), 7-bp deletion (CCGGGTC)	21-bp deletion (GAGGAGCCGTCGCCTATCGGG), 68-bp deletion (CGCCTATCGGGGACGGGTTCATTAGGTTCGAGTTCTTTGAAGATGGATGGGACGAGGATGAGCCCCGG)
23+24-2 (309)	1-bp mismatch (A/T), 9-bp deletion (TCAAGGGCA)	2-bp deletion (TG), 59-bp deletion (CAACAACCCGTTCATCCCGTGGGTGTCTGACGTGGCCGAGAGTCTCGGTCTACCCTCGG)
23+24-2 (312)	1-bp mismatch (C/T), 12-bp deletion (CGGGTCAAGGGC)	WT, 1-bp insertion (T)
23+24-2 (313)	WT, 1-bp deletion (G), 5-bp deletion (GTCAA), 124-bp deletion (TCTCGAATTATTAGCCGCAGAAGAAGAAGCAGAAGAAGGAATTACAGGTGAATTAGTTAGTTCATTATGGGTTCGGAGTCGTCACTTGTCCATGTCTTCTTGGTCTCCTTCCCGGGTCAAGGGC)	5-bp deletion (GTCTG), 7-bp deletion (GTCTGAC)
**23+24-2 (328)**	1-bp deletion (G), 3-bp deletion (TCA)	1-bp insertion (T), 6-bp deletion (TGACGT)
23+24-2 (333)	WT, 3-bp deletion (GTC), 10-bp deletion (GGGTCAAGGG)	WT, 1-bp insertion (A), 1-bp insertion (T)
23+24-2 (335)	3-bp deletion (GTC), 35-bp deletion (TCACTTGTCCATGTCTTCTTGGTCTCCTTCCCGGG)	1-bp insertion (A), 1-bp deletion (G)

sgRNA23 (23) targets *PgUGT84A23*, whereas sgRNA24-1 (24-1) and sgRNA24-2 (24-2) target different regions of *PgUGT84A24*. Specific hairy root line numbers are shown in parenthesis next to the sgRNA designation. For mutations that include a deletion and a mismatch, both the mutant and the reference wild-type sequences are shown. The *ugt84a23 ugt84a24* mutants that were subjected to transcriptome sequencing are highlighted in bold

WT, wild-type allele

For the 36 dual sgRNA lines (sgRNA23+sgRNA24-1/sgRNA24-2) selected for sequencing, 23 were identified as homozygous, biallelic, or chimeric mutants for *PgUGT84A24* and further examined for mutations in the *PgUGT84A23* alleles, 19 of which also showed homozygous, biallelic, heterozygous, or chimeric mutations in *PgUGT84A23* (Table 2). Interesting variations of CRISPR/Cas9 editing were observed in the dual sgRNA lines, e.g., a long deletion of 1162-bp in a *PgUGT84A24* allele in sgRNA23+sgRNA24-1 (line 305) and both a 21-bp mismatch and a 6-bp deletion in a *PgUGT84A23* allele in sgRNA23+sgRNA24-1 (line 321) (Table 2).

### Knockout of *Pg*UGT84A23 and *Pg*UGT84A24 led to changes in galloylglucose conjugates and derivatives in pomegranate hairy roots

**Fig. 2:**
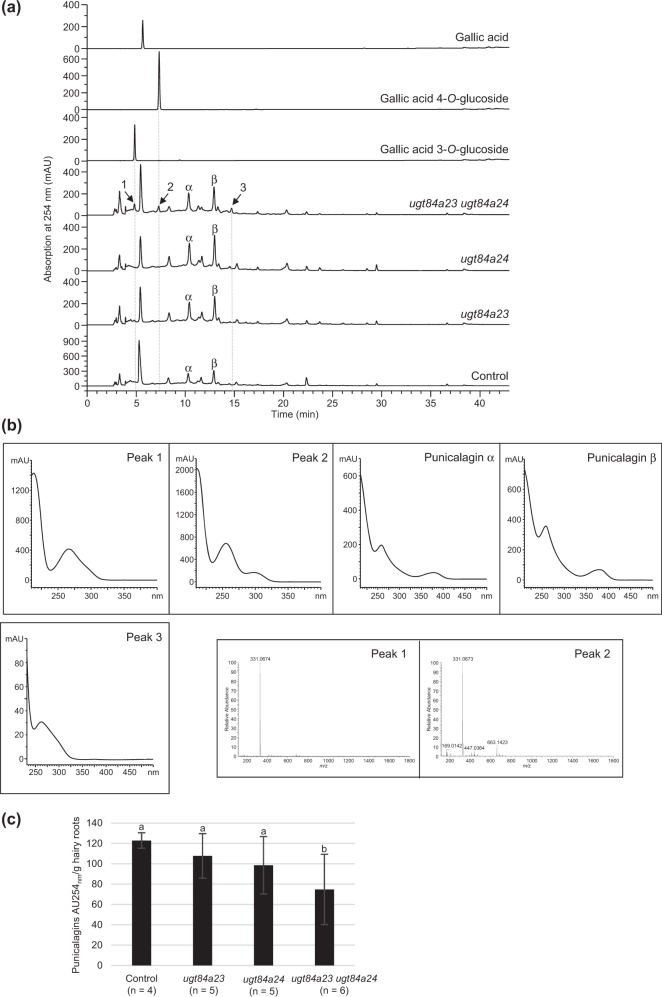
Metabolite analysis of the control and the CRISPR/Cas9-edited *PgUGT84A23* and/or *PgUGT84A24* hairy roots. **a** HPLC chromatograms of authentic standards as well as pomegranate hairy roots containing the vector plasmid (control) or the CRISPR/Cas9-edited *PgUGT84A23* and/or *PgUGT84A24* (*ugt84a23*, *ugt84a24*, and *ugt84a23 ugt84a24*). Representative chromatograms of the hairy root lines are shown. Peaks (1–3) are only present in *ugt84a23 ugt84a24* hairy roots and indicated with arrows. α, punicalagin α; β, punicalagin β. **b** Absorption spectra of peaks 1–3 and punicalagin α and β isomers. Mass spectra of peaks eluted at 4.85 min (peak 1) and 7.32 min (peak 2) are shown. **c** Peak areas of punicalagins (punicalagin α and β isomers) in the control and the CRISPR/Cas9-edited *PgUGT84A23* and/or *PgUGT84A24* hairy root lines. Different letters indicate significant differences (*P* < 0.05) in punicalagin peak areas.

To investigate the effect of the *PgUGT84A23* and/or *PgUGT84A24* mutations, phenolic metabolites in the control and mutant (*ugt84a23*, *ugt84a24*, *ugt84a23 ugt84a24*) hairy roots were analyzed (Fig. 2a). Eliminating *Pg*UGT84A23 or *Pg*UGT84A24 individually did not affect the metabolite profile significantly compared to the controls. However, when both UGT activities were abolished, punicalagins showed a 40% reduction in *ugt84a23 ugt84a24* (Fig. 2a, c). Moreover, three new peaks (peaks 1–3) appeared in the dual CRISPR/Cas9-edited lines *ugt84a23 ugt84a24* (Fig. 2a). The retention times and absorption spectra of peaks 1 and 2 matched those of the gallic acid 3-*O*- and 4-*O*-glucoside standards, respectively (Fig. 2a, b). Mass spectrometric (MS) analysis of peaks 1 and 2 confirmed that both compounds are conjugates of gallic acid and glucose ([M-H]^−^ at *m*/*z* 331.07) (Fig. 2b). In contrast to peaks 1 and 2 that were present in all of the *ugt84a23 ugt84a24* lines, peak 3 (Fig. 2a, b, unidentified) was only detectable in two-thirds of the *ugt84a23 ugt84a24* hairy roots.

### A regiospecific gallic acid 4-*O*-glycosyltransferase was discovered from transcriptome analysis of the CRISPR/Cas9-edited hairy roots and biochemical characterization

**Fig. 3:**
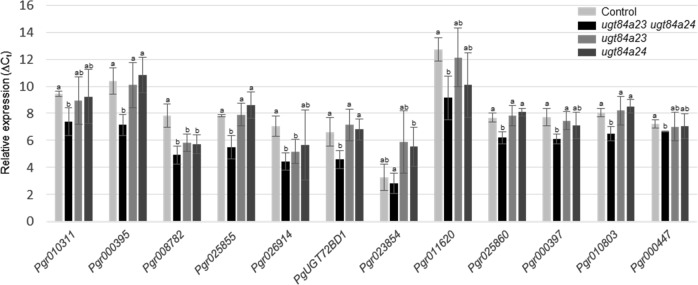
Real-time qPCR analysis of the candidate UGTs in the control and mutant (*ugt84a23*, *ugt84a24*, *ugt84a23 ugt84a24*) hairy roots. The relative expression is indicated by the Δ*C*_t_ value between the target gene and the housekeeping *PgActin* gene. Different letters indicate significant differences (*P* < 0.05) in the expression of the target gene.

**Fig. 4:**
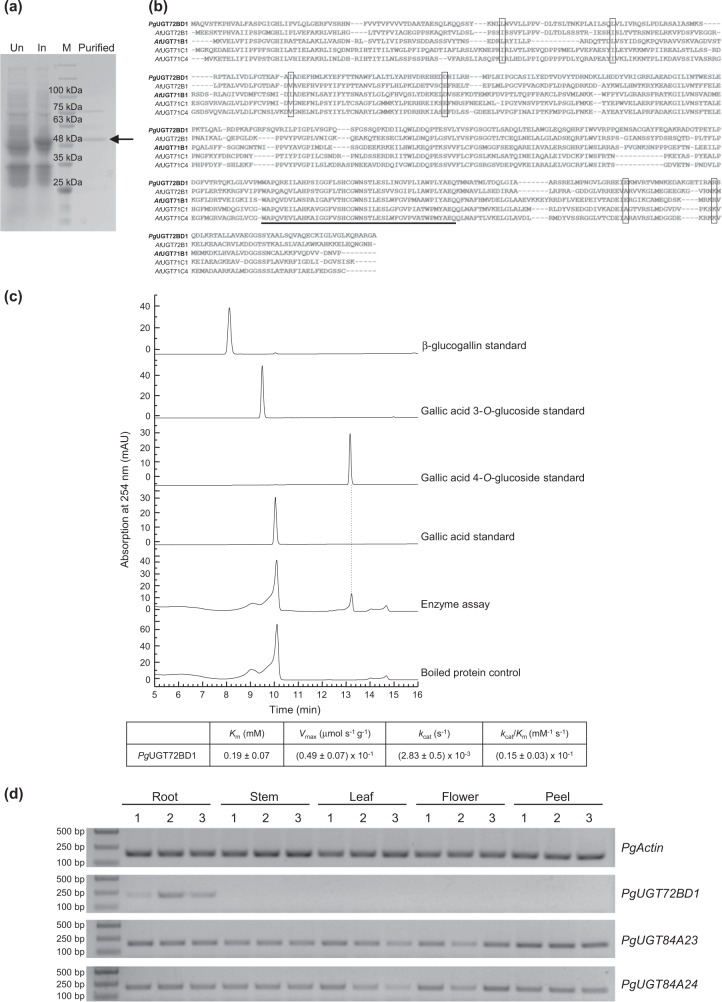
Molecular and biochemical characterization of *PgUGT72BD1*. **a** Expression and purification of the recombinant *Pg*UGT72BD1 protein in *E. coli*. The purified recombinant *Pg*UGT72BD1 is indicated by an arrow. Un, uninduced; In, induced; M, protein molecular weight marker. **b** Multi-sequence alignment of *Pg*UGT72BD1 and the group E UGTs previously shown to catalyze 3-*O*- or 4-*O*-glucosylation of hydroxybenzoic acid substrates. UGTs capable of 4-*O*-glucosylation are highlighted in bold. Amino acids that are common among 3-*O* or 4-*O* UGTs but differ between the two groups are indicated with boxes. The conserved Plant Secondary Product Glycosyltransferase (PSPG) motif is underlined. **c** *Pg*UGT72BD1 produces gallic acid 4-*O*-glucoside from gallic acid and UDP-glucose. The kinetic parameters of *Pg*UGT72BD1 toward gallic acid are shown. **d** Tissue-specific expression of *PgUGT72BD1*, *PgUGT84A23*, and *PgUGT84A24*. Total RNA was extracted from three biological replicates (1–3) of each pomegranate tissue type and used for reverse transcription (RT). The RT products served as templates for the semi-quantitative PCR reactions.

To identify the UGT activities that produce gallic acid glucosides in *ugt84a23 ugt84a24*, transcriptome analysis was conducted on the control (three independent lines) and the dual edited hairy roots (lines 324, 327, 328, and 346; Table 2). Twelve UGTs showed significantly increased expression (greater than two-fold) in the *ugt84a23 ugt84a24* lines compared to the controls (Table 3). Real-time quantitative polymerase chain reaction (qPCR) analysis confirmed that the transcript levels of 11 UGTs were higher in the *ugt84a23 ugt84a24* lines than the controls (Fig. 3). Interestingly, the expression of *Pgr008782* was also increased in the single CRISPR/Cas9-edited lines *ugt84a23* and *ugt84a24* (Fig. 3). These 11 candidate UGTs were expressed as recombinant proteins in *Escherichia coli* and the purified proteins were assayed with gallic acid and UDP-glucose as substrates. Of the 11 recombinant UGTs, only *Pg*UGT72BD1 was active toward gallic acid and formed a single product, gallic acid 4-*O*-glucoside (Fig. 4a, c). The steady-state kinetics of *Pg*UGT72BD1 showed that it had a relatively high affinity to gallic acid (*K*_m_ = 0.19 ± 0.07 mM) but a slow turnover [*k*_cat_ = (2.83 ± 0.5) × 10^−3^ s^−1^)] and low catalytic efficiency [*k*_cat_/*K*_m_ = (0.15 ± 0.03) × 10^−1^ mM^−1^ s^−1^] (Fig. 4c).

**Table 3:** UGTs that showed significantly increased expression in the *ugt84a23 ugt84a24* hairy roots compared to the control hairy roots in the transcriptome analysis.

Gene name	Control (TPM)	*ugt84a23 ugt84a24* (TPM)	Log_2_FC	Adjusted *P* value
*Pgr010311*	0.81	5.24	2.34	6.16E−08
*Pgr000395*	1.55	7.46	2.11	3.00E−05
*Pgr008782*	4.07	19.06	2.02	1.04E−07
*Pgr025855*	3.39	16.01	2.01	3.37E−07
*Pgr026914*	5.75	27.64	1.79	5.10E−04
*PgUGT72BD1*	8.66	32.65	1.60	2.75E−03
*Pgr023854*	0	0.45	1.60	3.00E−02
*Pgr011620*	0.22	2.27	1.60	3.34E−02
*Pgr025860*	3.61	10.54	1.41	4.49E−04
*Pgr000397*	3.59	9.81	1.32	1.03E−03
*Pgr010803*	57.04	147.54	1.30	4.81E−04
*Pgr000447*	10.87	26.70	1.18	7.87E−03

TPM, transcripts per million reads; FC, fold change; Adjusted *P* value, *P* value adjusted for multiple testing

**Fig. 5:**
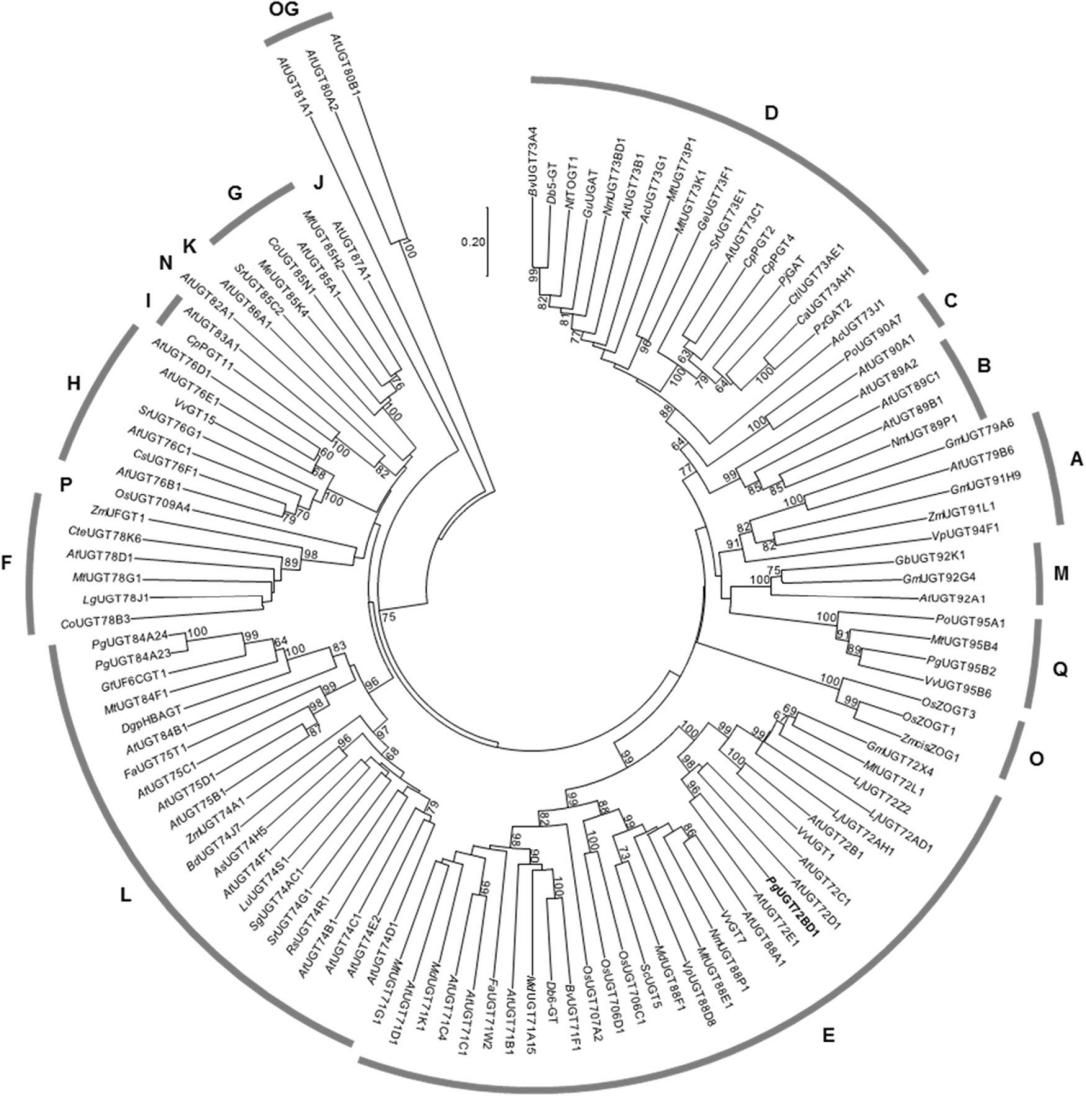
Phylogeny of functionally characterized pomegranate UGTs and selected UGTs representing different plant UGT phylogenetic groups. *Pg*UGT72BD1 is highlighted in bold. Bootstrap values >60 are shown next to the branches. OG, outgroup.

As with other plant UGTs, *Pg*UGT72BD1 also contains a conserved PSPG motif at the C-terminus of the protein (Fig. 4b). Signal peptides were not detected in *Pg*UGT72BD1, suggesting its localization in the cytosol (Fig. 4b). A phylogenetic analysis of representative UGTs placed *Pg*UGT72BD1 in group E, together with *At*UGT71B1, *At*UGT72B1, *At*UGT71C1, and *At*UGT71C4, the UGTs that were previously shown with 3-*O* or 4-*O*-glucosylation activities toward hydroxybenzoic acids (gallic acid was not tested as a substrate for these UGTs) (Fig. 5)^11^. When the protein sequences of *Pg*UGT72BD1, *At*UGT71B1, *At*UGT72B1, *At*UGT71C1, and *At*UGT71C4 were compared, six amino acid sites were identified that are common among the 3-*O* (*At*UGT72B1, *At*UGT71C1, and *At*UGT71C4) or 4-*O* (*Pg*UGT72BD1 and *At*UGT71B1) UGTs but differ between the two groups (Fig. 4b).

To understand the expression of *PgUGT72BD1* in different pomegranate tissues, its transcript levels were initially determined by real-time qPCR. However, with the exception of roots (*C*_t_ values around 27), the *C*_t_ values obtained from other tissues were >38, suggesting a very low abundance of *PgUGT72BD1* transcripts in stems, leaves, flowers, and fruit peels. Semi-qPCR was subsequently conducted, and consistent with the results from the real-time qPCR analysis, amplification products of *PgUGT72BD1* were only detected in the root tissue (Fig. 4d). In contrast, *PgUGT84A23* and *PgUGT84A24* were expressed in all tissues examined (Fig. 4d).

## Discussion

This study demonstrates that CRISPR/Cas9-based genome editing, *A. rhizogenes*-mediated hairy root transformation and non-destructive screening of transgenic hairy roots, as well as transcriptome analysis collectively enable efficient and effective gene discovery in pomegranate. The unique accumulation of gallic acid glucosides in the *ugt84a23 ugt84a24* hairy roots also suggests that the CRISPR/Cas9 method holds the potential for developing new pomegranate cultivars with modified phytochemical profiles. The presence of gallic acid 3-*O*- and 4-*O*-glucosides has only been reported in fruits of blackcurrant, gooseberry, jostaberry, raspberry, blackberry, blueberry, *Arbutus unedo* (strawberry tree), and grape (pomace)^12–14^. Therefore, it is interesting that gallic acid glucosides can also be found in a non-reproductive plant tissue.

The CRISPR/Cas9-sgRNAs generated mismatches, in-frame (3*n*, e.g., 3-bp, 6-bp), or out-of-frame (e.g., 1-bp, 4-bp, 5-bp) deletions, as well as insertions of 1-bp, 2-bp, or 7-bp in *PgUGT84A23* and *PgUGT84A24* (Tables 1 and 2). The above-mentioned insertions and the out-of-frame deletions are expected to result in a frameshift and incorrect translation of the protein. Because the mutant lines containing in-frame deletions (removal of amino acids) exhibited metabolic phenotypes similar to those with insertions and out-of-frame deletions, it suggests that the missing amino acids resulting from the in-frame deletions play important roles in enzyme activities. In addition to the homozygous, monoallelic, and biallelic mutants, chimeras of more than two mutated alleles were also identified in the CRISPR/Cas9-edited hairy roots (Table 2). It could be because the hairy roots induced at the inoculation sites contain heterogeneous cells of different CRISPR/Cas9-edited or non-edited gene alleles. To establish homogeneous hairy root clones, a single hairy root tip would need to be recultured in phytohormone-free growth medium for multiple rounds and then tested for homogeneity. On the other hand, the metabolic phenotype of *ugt84a23 ugt84a24* hairy roots (appearance of new peaks) indicates the advantage of metabolite profiling in detecting knockout of enzyme activities in a heterogeneous cell population.

Eliminating both *Pg*UGT84A23 and *Pg*UGT84A24 activities frees gallic acid from the biosynthesis of β-glucogallin (Fig. 1a). The transitory increase in the cellular gallic acid concentration may regulate the expression/activity of UGT(s) that convert gallic acid to the glucoside derivatives (Fig. 1a). Indeed, gallic acid 3-*O*- and 4-*O*-glucosides accumulated in the *ugt84a23 ugt84a24* hairy roots (Fig. 2). In addition, transcriptome and real-time qPCR analyses identified 11 UGTs with increased expression in *ugt84a23 ugt84a24* and one of the candidate UGTs, *Pg*UGT72BD1, exhibited regioselective glucosylation of gallic acid at the 4-OH position (Table 3; Figs. 3 and 4). However, none of the candidate UGTs produced gallic acid 3-*O*-glucoside, suggesting that the gallic acid 3-*O*-glucosylation activity may be regulated at a level other than transcription.

*Pg*UGT72BD1, *Pg*UGT84A23, and *Pg*UGT84A24 can all use gallic acid as substrate but form galloylglucose conjugates with ether or ester linkages (Fig. 1a). *Pg*UGT72BD1 has a higher affinity to gallic acid (*K*_m_ = 0.19 ± 0.07 mM; Fig. 4c) than *Pg*UGT84A23 (*K*_m_ = 0.89 ± 0.07 mM) and *Pg*UGT84A24 (*K*_m_ = 0.98 ± 0.01 mM)^10^. However, the turnover numbers of *Pg*UGT84A23 (*k*_cat_ = 0.52 ± 0.03 s^−1^) and *Pg*UGT84A24 (*k*_cat_ = 0.55 ± 0.01 s^−1^) are >180-fold higher than that of *Pg*UGT72BD1 [*k*_cat_ = (2.83 ± 0.5) × 10^−3^ s^−1^]. As a result, the catalytic efficiency of *Pg*UGT72BD1 [*k*_cat_/*K*_m_ = (0.15 ± 0.03) × 10^−1^ mM^−1^ s^−1^] is about 38-fold lower than that of *Pg*UGT84A23 (*k*_cat_/*K*_m_ = 0.58 mM^−1^ s^−1^) and *Pg*UGT84A24 (*k*_cat_/*K*_m_ = 0.56 mM^−1^ s^−1^) (Fig. 4c)^10^. Therefore, even though *PgUGT72BD1* is expressed in the wild-type pomegranate roots and hairy roots, gallic acid is mainly used for the biosynthesis of β-glucogallin (and HTs) by *Pg*UGT84A23 and *Pg*UGT84A24 due to their much higher catalytic efficiencies than *Pg*UGT72BD1. Indeed, our previous metabolite profiling analysis did not identify gallic acid 4-*O*-glucoside in any pomegranate tissues^10^. These results also suggest that the primary role of *Pg*UGT72BD1 in pomegranate roots could be glycosylating aglycones other than gallic acid. Intriguingly, HT production was not completely abolished in *ugt84a23 ugt84a24* (Fig. 2), suggesting that there could be additional UGT(s) contributing to β-glucogallin formation in pomegranate.

Of the 17 so far defined UGT phylogenetic groups (A–Q) in plants, *Pg*UGT72BD1 (gallic acid 4-*O* UGT) belongs to group E that contains UGT71, UGT72, and UGT88 gene families (Fig. 5). Regioselective glycosylation of hydroxybenzoic acids (structurally similar to gallic acid) was previously reported for members of group E UGTs, including *At*UGT71B1 that only glycosylates the 4-OH position and *At*UGT71C1, *At*UGT71C4, and *At*UGT72B1 that specifically glycosylate the 3-OH position^11^. Six amino acids are conserved in the hydroxybenzoic acid/gallic acid 3-*O* or 4-*O* UGTs but distinct between the two groups of regioselective UGTs (Fig. 4b). The function of these amino acids in determining the regioselectivity of the corresponding UGTs can be explored by site-directed mutagenesis and enzyme assays. In addition, once the gallic acid 3-*O* UGT is cloned in pomegranate, the protein sequences and structural features of the gallic acid 3-*O* and 4-*O* UGTs can be compared to identify the key amino acid(s) for regioselectivity. Furthermore, it was proposed that the regioselectivity for hydroxycoumarins (a group of phenolic metabolites) was switched among the UGT71, UGT72, and UGT88 families during the evolution of group E UGTs^15^. It will be interesting to understand whether regioselectivity switching event(s) for gallic acid also occurred among these UGT gene families.

## Conclusions

In this work, the CRISPR/Cas9-mediated editing of two galloylglucose ester-forming UGTs, *PgUGT84A23* and *PgUGT84A24*, in pomegranate hairy roots generated various mismatches, insertions, and deletions in the target genes. Metabolite analysis of the transgenic hairy roots showed modified phenolic profiles, particularly the accumulation of 3-*O*- and 4-*O*-glucosides of gallic acid, in the *ugt84a23 ugt84a24* double mutant lines. Transcriptome and real-time qPCR analyses identified multiple UGTs with increased expression in the *ugt84a23 ugt84a24* hairy roots compared to the vector-transformed controls. Biochemical characterization of the candidate genes discovered a group E UGT (*Pg*UGT72BD1) that glycosylates specifically the 4-*O* position of gallic acid.

The pomegranate genome has recently been sequenced, providing an exciting opportunity for exploring this ancient fruit and modern functional food^16,17^. Together with genome, transcriptome, and metabolite analyses, the CRISPR/Cas9 method renders functional genomics in pomegranate, a woody plant and non-traditional model system, more accessible. In addition, building a genome-editing platform in pomegranate will also facilitate germplasm improvement as well as the sustainable development of pomegranate as a horticultural crop and functional food.

## Materials and methods

### Construction of expression plasmids for Cas9 and sgRNAs

The sgRNAs for *PgUGT84A23* and *PgUGT84A24* were designed using Cas-Designer (http://www.rgenome.net/cas-designer/)^18^; those with high-quality scores were then subjected to testing for secondary structures using the mfold web server (http://mfold.rna.albany.edu/?=mfold/RNA-Folding-Form2.3)^19^. The following sgRNAs were selected in this study: sgRNA23 for *PgUGT84A23* (5′-GGGTCAAGGGCACGTGAAC-3′) and two sgRNAs, sgRNA24-1 (5′-GGGAGGAGCCGTCGCCTAT-3’) and sgRNA24-2 (5′-ATCCCGTGGGTGTCTGACG-3′), for *PgUGT84A24*.

The sgRNAs were cloned into the psgR-Cas9-At backbone, which contains the *AtU6* promoter for the expression of the sgRNA as well as the *AtUBQ1* promoter and terminator for the expression of *Sp*Cas9^20^. For easy identification of transgenic hairy roots, the hygromycin B resistance gene in the plant transformation vector pCAMBIA1300 was replaced with a GFP gene and the resulting plasmid vector was designated pCAMBIA1300-GFP. The sgRNA and Cas9 containing psgR-Cas9-At cassette was cloned into the *EcoR*I and *Hind*III sites of pCAMBIA1300-GFP. Because a high-quality sgRNA targeting both *PgUGT84A23* and *PgUGT84A24* was not identified, the sgRNAs for *PgUGT84A23* and *PgUGT84A24* were cloned into the p2xsgR-Cas9-At cassette (i.e., sgRNA23+sgRNA24-1 or sgRNA23+sgRNA24-2) where the two sgRNAs were each directed by an *AtU6* promoter. The resulting cassettes were cloned into the *EcoR*I and *Hind*III sites of pCAMBIA1300-GFP.

### *Agrobacterium rhizogenes*-mediated induction and transformation of pomegranate hairy roots

The sgRNA and Cas9-expressing pCAMBIA1300-GFP plasmids and the empty pCAMBIA1300-GFP vector were transformed into the *A. rhizogenes* strain MSU440 through electroporation. Induction and transformation of pomegranate hairy roots using the hypocotyl explants were carried out as described^9^. The hairy roots were observed under a fluorescent microscope (Leica DM6000B, Leica Microsystems, Wetzlar, Germany) after 21 days. Hairy roots that emitted green fluorescence upon excitation were marked on the plate and the non-green fluorescent hairy roots were removed using a scalpel. Only one green fluorescent hairy root was maintained on each plate. These hairy roots were transferred to plates containing fresh growth media every 3 weeks. After about 2 months, the hairy root tissue was collected, frozen in liquid nitrogen, and ground into fine powder using mortar and pestle.

### Detection of CRISPR/Cas9-mediated gene editing

To identify the CRISPR/Cas9-edited *PgUGT84A23* and *PgUGT84A24* alleles, genomic DNA was extracted from transgenic hairy roots using a Cetyltrimethyl Ammonium Bromide-based method^21^. PCR reactions were performed using the genomic DNA as template and *PgUGT84A23F* (5′-GTTCGGAGTCGTCACTTGTC-3′) and *PgUGT84A23R* (5′-ATCTCGTGCTCAAGTTCCTG-3′) for amplification of the *PgUGT84A23* alleles and *PgUGT84A24F* (5′-GGGGTCCGAGTCGTTGGTTC-3′) and *PgUGT84A24R* (5′-GCACGGCAACTGGACATCG-3′) for the *PgUGT84A24* alleles. The PCR products were analyzed directly by DNA sequencing. The DNA sequence chromatograms of the homozygous wild-type or mutant alleles showed individual, evenly distributed peaks. When a mixture of different mutant alleles or a combination of wild-type and mutant alleles was present in the PCR products, the chromatograms displayed overlapping peaks. In the latter case, the PCR products were cloned into a TA cloning vector pMD19-T (Takara Biomedical Technology Co., Ltd., Beijing, China). The resulting plasmids were transformed into *E. coli* DH5α cells and multiple colonies were selected for plasmid preparation and DNA sequencing.

### Metabolite analysis of transgenic hairy roots

The ground hairy root tissue was extracted in 70% methanol for 60 min under sonication and centrifuged at 13,000 rpm for 10 min. The supernatant was transferred to an high-performance liquid chromatography (HPLC) vial and 20 μL was injected onto an Agilent 1200 HPLC. Metabolite separation was performed using a reverse-phase C_18_ column (Diamonsil, 250 mm × 4.6 mm, particle size 5 μm) and a previously established gradient^22^. Peaks of interest were collected from multiple HPLC runs, pooled, concentrated, and subjected to high-resolution electrospray ionization MS analysis as described^22^. The gallic acid, gallic acid 3-*O*-glucoside, and gallic acid 4-*O*-glucoside standards were purchased from ZZBIO Co. LTD (Shanghai, China). One-way analysis of variance (ANOVA) followed by Tukey’s honestly significant difference (HSD) post hoc test was conducted on the peak areas of punicalagins (punicalagin α and β isomers) using JMP 14.2.0 (SAS Institute, 2018).

### Transcriptome analysis of transgenic hairy roots

Three vector-transformed and four *ugt84a23 ugt84a24* hairy root lines (Table 2) were selected for comparative transcriptome analysis. Total RNA was extracted from hairy roots using Trizol reagent (Invitrogen, Carlsbad, CA). RNAseq libraries were constructed using the Illumina TruSeq RNA Sample Prep Kit and subjected to sequencing on an Illumina HiSeq4000, with about 60 million paired-end reads (2 × 150 bp) per sample. The raw reads were cleaned to remove the adapter sequences as well as short or low-quality sequences using SeqPrep (https://github.com/jstjohn/SeqPrep) and Sickle (https://github.com/najoshi/sickle). The trimmed reads were mapped to the pomegranate genome using Hisat2 to obtain read counts^23^. The read counts were quantified by the RNA-Seq by Expectation Maximization method and expressed as transcripts per million reads^24^. The differential gene expression analysis between the controls and *ugt84a23 ugt84a24* lines was performed using DESeq2, with adjusted *P* value <0.05 and |log_2_FC| ≥ 1^25^. The transcriptome data were deposited in the NCBI sequence reads archive under the accession PRJNA550088.

### Expression and purification of recombinant proteins and UGT enzyme assays

The open reading frames of candidate UGTs were codon optimized for *E. coli* expression, synthesized by Genewiz (Suzhou, China), and cloned into the pET28a vector. The recombinant plasmids were transformed into *E. coli* BL21 (DE3) and the cells were grown at 37 °C in the Luria Bertani media until OD_600_ reached 0.8. Protein expression was induced by adding isopropyl β-D-1-thiogalactopyranoside to a final concentration of 0.1 mM. The cells were grown at 17 °C for an additional 18 h and harvested by centrifugation. The cell pellets were resuspended in the lysis buffer (50 mM MES, pH 5.5, 300 mM NaCl, and 50 mM imidazole) and homogenized using a cell disruptor (Constant Systems Ltd, Northants, UK). The His-tagged recombinant UGT proteins were purified using Ni-NTA resin (Thermo Fisher Scientific, Waltham, MA, USA). The concentration of the purified proteins was measured using the Bradford assay^26^. The purified protein was kept in the storage buffer [50 mM MES, pH 5.5, 100 mM NaCl, and 10% (w/v) glycerol] at −80 °C.

For UGT enzyme assays, the 160-μL reaction mixture contained 50 mM MES, pH 5.5, 0.6 mM UDP-glucose, 0.25 mM gallic acid, 3 mM 2-mercaptoethanol, and 1.6 μg of purified protein. After incubating at 30 °C for 12 h, the reaction was terminated by adding 16 μL of trifluoroacetic acid (100%, w/v) and 400 μL of methanol. The reaction mixture was filtered through a 0.22-μm filter and 70 μL of the flow-through was injected onto an Agilent 1200 HPLC with a reverse-phase C_18_ column (YMC-Pack ODS-AQ C_18_ L1, 250 mm x 4.6 mm, particle size 5 μm). The elution gradient was between (A) 0.1% formic acid in water and (B) acetonitrile at 0–3 min, 3% B; 3–5 min, 3–5% B; 5–15 min, 5–15% B; 15–16 min, 15–60% B; and 16–18 min, 60–3% B. The flow rate was 1 mL min^−1^. The kinetic analysis of *Pg*UGT72BD1 with gallic acid as substrate was performed as previously described^27^, with slight modifications. The gallic acid concentrations were between 30 and 500 μM and the reactions were incubated at 30 °C for 45, 75, and 135 min.

### Reverse transcription (RT) (semi)-qPCR analysis

Total RNA was extracted from transgenic hair roots or pomegranate leaf, stem, root, flower, and fruit peel tissues using the RNAprep Pure Plant Kit (Tiangen Biotech Co., Ltd., Beijing, China) and used for RT with the PrimeScript™ RT Reagent Kit (Takara). qPCR was performed using the TB Green^®^ Premix Ex Taq™ (Tli RNaseH Plus) Kit (Takara) on a StepOnePlus Real-Time PCR System (Thermo Fisher Scientific). Melting curve analysis was conducted immediately after the PCR reactions and only one product was observed for each primer pair. The RT-qPCR reactions were performed using three biological replicates, each with three technical replicates. The expression levels of candidate UGTs in the control and mutant (*ugt84a23*, *ugt84a24*, *ugt84a23 ugt84a24*) samples were presented using Δ*C*_t_ values (*C*_t *PgUGT*_ − *C*_t *PgActin*_). Statistical analysis was conducted using ANOVA and Tukey’s HSD test in JMP 14.2.0 (SAS Institute). The primer sequences, amplicon sizes, and amplification efficiencies are shown in Table S1.

For the semi-qPCR analysis, 1 μL of the first-strand cDNA was used as template for amplification by TaKaRa Taq^®^ DNA polymerase (Takara) and primers specific for *PgUGT72BD1*, *PgUGT84A23*, *PgUGT84A24*, or *PgActin* (Table S1). The PCR conditions were as follows: 94 °C for 5 min, followed by 25 cycles (*PgActin*, *PgUGT84A2*3, *PgUGT84A24*) or 30 cycles (*PgUGT72BD1*) of 94 °C for 30 s, 60 °C for 30 s, and 72 °C for 45 s, and a final extension step of 72 °C for 10 min. The PCR products were analyzed on a 1.5% agarose gel.

### Phylogenetic analysis

Alignment of plant UGT sequences was conducted using Multiple Sequence Comparison by Log-Expectation (MUSCLE)^28^. A neighbor-joining tree was built using Molecular Evolutionary Genetics Analysis and assessed with 1000 bootstrap replicates^29^. The AGI (Arabidopsis sequences) and GenBank (non-Arabidopsis sequences) accession numbers for the UGTs are: *Ac*UGT73G1 (AAP88406), *Ac*UGT73J1 (AAP88407), *As*UGT74H5 (ACD03250), *At*UGT71B1 (AT3G21750), *At*UGT71C1 (AT2G29750), *At*UGT71C4 (AT1G07250), *At*UGT71D1 (AT2G29730), *At*UGT72B1 (AT4G01070), *At*UGT72C1 (AT4G36770), *At*UGT72D1 (AT2G18570), *At*UGT72E1 (AT3G50740), *At*UGT73B1 (AT4G34138), *At*UGT73C1 (AT2G36750), *At*UGT74B1 (AT1G24100), *At*UGT74C1 (AT2G31790), *At*UGT74D1 (AT2G31750), *At*UGT74E2 (AT1G05680), *At*UGT74F1 (AT2G43840), *At*UGT75B1 (AT1G05560), *At*UGT75C1 (AT4G14090), *At*UGT75D1 (AT4G15550), *At*UGT76B1 (AT3G11340), *At*UGT76C1 (AT5G05870), *At*UGT76D1 (AT2G26480), *At*UGT76E1 (AT5G59580), *At*UGT78D1 (AT1G30530), *At*UGT79B6 (AT5G54010), *At*UGT80B1 (AT1G43620), *At*UGT80A2 (AT3G07020), AtUGT81A1 (AT4G31780), *At*UGT82A1 (AT3G22250), *At*UGT83A1 (AT3G02100), *At*UGT84B1 (AT2G23260), *At*UGT85A1 (AT1G22400), *At*UGT86A1 (AT2G36970), *At*UGT87A1 (AT2G30150), *At*UGT88A1 (AT3G16520), *At*UGT89A2 (AT5G03490), *At*UGT89B1 (AT1G73880), *At*UGT89C1 (AT1G06000), *At*UGT90A1 (AT2G16890), *At*UGT92A1 (AT5G12890), *Bd*UGT74J7 (XP_003581017), *Bv*UGT71F1 (AY526081), *Bv*UGT73A4 (AY526080), *Ca*UGT73AH1 (AUR26623), *Co*UGT78B3 (AEB61484), *Co*UGT85N1 (AEB61489), *Cp*PGT2 (AIS39471), *Cp*PGT4 (AIS39473), *Cp*PGT11 (AIS39477), *Cs*UGT76F1 (KDO69246), *Cte*UGT78K6 (BAF49297), *Cti*UGT73AE1 (AJT58578), *Db*5-GT (CAB56231), *Db*6-GT (AAL57240), *Dg*pHBAGT (BAO66179), *Fa*UGT71W2 (XP_011468178), *Fa*UGT75T1 (XP_004307485), *Gb*UGT92K1 (ASK39406), *Ge*UGT73F1 (BAC78438), *Gm*UGT72X4 (KRH46505), *Gm*UGT79A6 (BAN91401), *Gm*UGT91H9 (NP_001348424), *Gm*UGT92G4 (KRH14708), *Gt*UF6CGT (AB985754), *Gu*UGAT (ANJ03631), *Lg*UGT78J1 (AEB61487), *Lj*UGT72AD1 (AP009657), *Lj*UGT72AH1 (AOG18241), *Lj*UGT72Z2 (AKK25344), *Lu*UGT74S1 (AGD95005), *Md*UGT71A15 (AAZ80472), *Md*UGT71K1 (ACZ44835), *Md*UGT88F1 (ARV88476), *Me*UGT85K4 (AEO45781), *Mt*UGT71G1 (AAW56092), *Mt*UGT72L1 (ACC38470), *Mt*UGT73K1 (AAW56091), *Mt*UGT73P1 (ABI94026), *Mt*UGT78G1 (ABI94025), *Mt*UGT84F1 (ABI94023), *Mt*UGT85H2 (ABI94024), *Mt*UGT88E1 (ABI94021), *Mt*UGT95B4 (XP_003612636), *Nm*UGT73BD1 (LC368259), *Nm*UGT88P1 (CEO43476), *Nm*UGT89P1 (LC368262), *Nt*TOGT1 (AAB36653), *Os*UGT706C1 (BAB68090), *Os*UGT706D1 (BAB68093), *Os*UGT707A2 (BAC83994), *Os*UGT709A4 (BAC80066), *Os*ZOGT1 (BAS90436), *Os*ZOGT3 (BAS90518), *Pg*UGT72BD1 (MN124519), *Pg*UGT84A23 (ANN02875), *Pg*UGT84A24 (ANN02877), *Pg*UGT95B2 (MH507175), *Pj*GAT (AYA60333), *Po*UGT90A7 (EU561019), *Po*UGT95A1 (ACB56927), *Pz*GAT2 (AYA60331), *Rs*UGT74R1 (ABP49574), *Sc*UGT5 (BAJ11653), *Sg*UGT74AC1 (AEM42999), *Sr*UGT73E1 (AAR06917), *Sr*UGT74G1 (AY345982), *Sr*UGT76G1 (AAR06912), *Sr*UGT85C2 (AAR06916), *Vp*UGT88D8 (BAH47552), *Vp*UGT94F1 (BAI44133), *Vv*GT7 (XP_002276546), *Vv*GT15 (XP_002281513), *Vv*UGT1 (CBI34463), *Vv*UGT95B6 (XP_010664783), *Zm*UFGT1 (P16167), *Zm*UGT74A1 (NP_001105326), *Zm*UGT91L1 (NP_001347041), and *Zm*cisZOG1 (AAK53551). *Ac*, *Allium cepa*; *As, Avena strigosa*; *At*, *Arabidopsis thaliana*; *Bd, Brachypodium distachyon*; *Bv*, *Beta vulgaris*; *Ca, Centella asiatica*; *Co, Consolida orientalis*; *Cp, Citrus paradise*; *Cs, Citrus sinensis*; *Cte, Clitoria ternatea*; *Cti, Carthamus tinctorius*; *Db, Dorotheanthus bellidiformis*; *Dg, Delphinium grandiflorum*; *Fa*, *Fragaria* × *ananassa*; *Gb*, *Ginkgo biloba*; *Ge, Glycyrrhiza echinata*; *Gm*, *Glycine max*; *Gt*, *Gentiana triflora*; *Gu, Glycyrrhiza uralensis*; *Lg, Lamium galeobdolon*; *Lj, Lotus japonicus*; *Lu, Linum usitatissimum*; *Md, Malus* × *domestica*; *Me, Manihot esculenta*; *Mt*, *Medicago truncatula*; *Nm, Nemophila menziesii*; *Nt, Nicotiana tabacum*; *Os*, *Oryza sativa*; *Pg*, *Punica granatum*; *Pj, Panax japonicus*; *Po*, *Pilosella officinarum*; *Pz, Panax zingiberensis*; *Rs, Rhodiola sachalinensis*; *Sc, Sinningia cardinalis*; *Sg, Siraitia grosvenorii*; *Sr, Stevia rebaudiana*; *Vp, Veronica persica*; *Vv*, *Vitis vinifera*; *Zm, Zea mays*.

## Supplementary information

Table S1

Figure S1
