# Supplementary material for: Effective genome editing and identification of a regiospecific gallic acid 4-O-glycosyltransferase in pomegranate (Punica granatum L.)
Source: Hortic Res. 2019 Nov 8;6:123. doi: 10.1038/s41438-019-0206-7 (PMC6838055; doi:10.1038/s41438-019-0206-7)
Supplement: Supplementary file 1 — Table S1 [file 41438_2019_206_MOESM1_ESM.docx]

**Table S1.** Primers used for the real-time- and semi-qPCR reactions. The amplicon sizes and amplification efficiencies of the primer pairs are indicated. -, primers were used for the semi-qPCR analysis and amplification efficiency was not tested.

| Gene name | Forward | Reverse | Amplicon size (bp) | Amplification efficiency |
| --- | --- | --- | --- | --- |
| *Pgr010311* | TTCGGTTCGTCACGCTTCCTA | CGGGATGTTCCTCCTTTTGCC | 203 | 89.8 % |
| *Pgr008782* | CTTGCCTGACTGCCCATCGTA | AATGAGTTGAACAGCACGCCC | 135 | 97.1 % |
| *Pgr025855* | AGCCCAAAGAAGTGTCTCCGA | TATGACATCCCCTGTGTTGGCA | 222 | 94.2 % |
| *Pgr000395* | CTGAACGGGCAGGAAAGAAAC | CGCCTCTATTCCAACTGCCAC | 155 | 104.9 % |
| *Pgr025860* | CTCCCCAAATGCTTCCAACTA | GTCGGTCTCGTCCAACATCAG | 155 | 106.9 % |
| *Pgr010803* | CTCCCCAAATGCTTCCAACTA | GTCGGTCTCGTCCAACATCAG | 175 | 104.2 % |
| *Pgr026914* | AGATTTGATAAGGCTGCTGCG | CATAGGTTGCTCCCAATGTGC | 179 | 96.3 % |
| *Pgr000397* | CTCAATGTCCGTTACCTCCGC | GGCTGACCGATGACTCAAAAA | 169 | 98.8 % |
| *PgUGT72BD1* | CACCAGCCTCACGAATAAACC | AGCGTATAAGGTGAGTGCGAGAA | 230 | 90.9 % |
| *Pgr000447* | GCCGAAGGAATCATTGCTAATAAGA | CGACTGTGGGGTGGTTGAGAA | 109 | 102. 9% |
| *Pgr023854* | GGATGTCCCCTCCCTCTGCTAC | AAGTAGCCCAAGAGCCCACAAG | 223 | 118.3 % |
| *Pgr011620* | CTGATGGATTTGACGACGGTTC | GCCTTCTTTCAGGTTGTTCTCG | 122 | 94.8 % |
| *PgActin* | TTGGAGATCCACATCTGCTG | TCCACCATGTTCCCTGGTAT | 170 | 103.2 % |
| *PgUGT84A23* | AGGCAGCGGAGATGAAGAAAA | TGTCTCTACCACCGATTCTGCG | 203 | - |
| *PgUGT84A24* | CGAGAACAAGCTGATCATGCG | GGCTTGGGTTCCGACTTGTTT | 222 | - |
