# Supplementary material for: Effective genome editing and identification of a regiospecific gallic acid 4-O-glycosyltransferase in pomegranate (Punica granatum L.)
Source: Hortic Res. 2019 Nov 8;6:123. doi: 10.1038/s41438-019-0206-7 (PMC6838055; doi:10.1038/s41438-019-0206-7)
Supplement: Supplementary file 2 — Figure S1 [file 41438_2019_206_MOESM2_ESM.pdf]

```
AtUGT81A1      1 --------------------------------MQNPSTVTQESAAPVFDFFPRLRGLTSR
AtUGT80A2      1 ----------------MPEISPAELAKVSSSSSSSSSSSSGRASVKIEE----IEGGAAA
AtUGT80B1      1 ----------------------------------------MASNVFDHP-LQELEGEDNG
OsZOGT3        1 --------------------------------MAEKGNPANKVAIVAVP-FP-AQGHLNQ
OsZOGT1        1 ----------------------------------MAIGTVESVAVVAVP-FP-AQGHLNQ
ZmcisZOG1      1 ----------------------------------MAVDTMESVAVVAVP-FP-AQGHLNQ
GmUGT79A6      1 -------------------------------MPSELAMNNDELHVVMFP-FL-AFGHISP
AtUGT79B6      1 --------------------------------------MGSKFHAFMFP-WF-GFGHMTA
GmUGT91H9      1 ----------------------------------------MPLHIAMLP-WL-AVGHVNP
ZmUGT91L1      1 ----------------------------------MAAADSSPLHIVIFP-WL-AFGHLIP
PoUGT95A1      1 --------------------MDNETQIKKRKLQTATMEAKATGEIFVVP-FF-GQGHLFP
MtUGT95B4      1 ----------------------------------------MSQEICILP-FF-GQGHLLP
PgUGT95B2      1 ----------------------------------------MALEILVVP-FF-GQGHLLP
VvUGT95B6      1 ----------------------------------------MSAHIIVFP-FF-GQGHLLP
AtUGT82A1      1 ------------------------------------MKVTQKPKIIFIP-YP-AQGHVTP
VpUGT94F1      1 ----------------------------------MEKEEAKMFKILMFP-WL-AHGHIFP
AtUGT87A1      1 -----------------------------------------------MP-WP-GRGHINP
AtUGT72C1      1 ----------------------------------------MELHGALVA-SP-GMGHAVP
AtUGT72D1      1 ---------------------------------------MDQPHALLVA-SP-GLGHLIP
LjUGT72AD1     1 ------------------------------------METTTTTHIAIVL-VP-IYSHIRS
AtUGT72B1      1 ------------------------------------MEESKTPHVAIIP-SP-GMGHLIP
VvUGT1         1 -------------------------------------MEHPTPHIAIVP-NP-GMGHLIP
LjUGT72AH1     1 --------------------------------------MEARTHIALVS-IP-ALSHQVS
LjUGT72Z2      1 --------------------------------------MAKTIHIAAVS-IP-AFSHQAS
GmUGT72X4      1 --------------------------------------MAKTTHIAIVS-SP-GYTHLVP
MtUGT72L1      1 -------------------------------MNLASNFMDKTIHIAVVP-GV-GYGHLVP
PgUGT72BD1     1 -----------------------------------MAQVSTKPHVALFA-SP-GIGHLIP
AtUGT72E1      1 ----------------------------------------------MFA-SP-GMGHIIP
OsUGT706C1     1 -------------------------------------MAMVEKTVLLYP-CP-AVGHLNP
OsUGT706D1     1 ------------------------------------MESKPSRNVVLYA-AM-GAGHLLP
MtUGT88E1      1 ----------------------------------------MKDTIVLYP-AF-GSGHLMS
VpUGT88D8      1 ----------------------------------------MEDTIILYA----SSVHLNS
NmUGT88P1      1 ---------------------------------------MEKKTIILYP-SP-GIGHLVS
AtUGT88A1      1 ---------------------------------------MGEEAIVLYP-AP-PIGHLVS
VvGT7          1 -----------------------------------------MESVVLYP-SP-GMGHLIS
MdUGT88F1      1 ----------------------------------------MGDVIVLYA-SP-GMGHIVS
ScUGT5         1 ----------------------------------------MEDTIVLYP-AP-GIGHLLS
OsUGT707A2     1 ---------------------------------MAPAMASSAATVVLIP-FC-VSGHLTP
AtUGT71B1      1 ----------------------------------------MKVELVFIP-SP-GVGHIRA
MdUGT71A15     1 --------------------------------------MKRPAQLVFVP-AP-GIGHIVS
BvUGT71F1      1 -----------------------------MSSSTASLTVTADAELVFVP-AP-GMGHLKS
Db6-GT         1 ---------------------------------------MSKIELVLVP-TP-GMGHLLS
AtUGT71C1      1 ------------------------------------MGKQEDAELVIIP-FP-FSGHILA
AtUGT71C4      1 --------------------------------------MVKETELIFIP-VP-STGHILV
AtUGT71D1      1 ---------------------------------------MRNVELIFIP-TP-TVGHLVP
FaUGT71W2      1 ---------------------------------------MKRAELVFIP-TP-STGHLVS
MtUGT71G1      1 ---------------------------------MSMSDINKNSELIFIP-AP-GIGHLAS
MdUGT71K1      1 ---------------------------------------MKKVELVFIP-SP-GAGHHLP
AtUGT89A2      1 -------------------------MTEVLLLPGTKSENSKPPHIVVFP-FP-AQGHLLP
AtUGT89C1      1 ----------------------------------MTTTTTKKPHVLVIP-FP-QSGHMVP
AtUGT89B1      1 ------------------------------MKVNEENNKPTKTHVLIFP-FP-AQGHMIP
NmUGT89P1      1 ----------------------------------MPSILSNSAHILLFP-FP-TSGHIIP
AtUGT92A1      1 ----------------------------------MAEAKPRNLRIVMFP-FM-GQGHIIP
GbUGT92K1      1 --------------------------------MAAGSQETVRMHLVMFP-YM-AQGHIIP
GmUGT92G4      1 -----------------------------------MAETPNKGHIVMVP-LM-AQGHLIP
PoUGT90A7      1 ------------------------------MASLLPDNSTHRPHFVLFP-FM-SKGHTIP
AtUGT90A1      1 -----------------------------------MSVSTHHHHVVLFP-FM-SKGHIIP
AcUGT73J1      1 -----------------------------------MAFDKKQLHILFFP-LM-SPGHFIP
MtUGT73P1      1 -----------------------------MESFGVKVEEETMLKAVFLP-FI-SKSHLIF
SrUGT73E1      1 -------------------------------MSPKMVAPPTNLHFVLFP-LM-AQGHLVP
CpPGT4         1 -----------------------------------MASEASQVHFLLLP-YL-APGHLIP
AtUGT73C1      1 ----------------------------------MASEFRPPLHFVLFP-FM-AQGHMIP
CpPGT2         1 -----------------------------------MDTQANQLHFVLFP-FL-AQGHMIP
PjGAT          1 -----------------------------MSQSPAMSKMQNQLHFVLVP-LL-AQGHMIP
CtiUGT73AE1    1 ----------------------------------MAPESASRIHFLLIP-LL-APGHTIP
CaUGT73AH1     1 -----------------------------------MDSQFQQLHFVMIP-LM-CPGHLIP
PzGAT2         1 -----------------------------------MDSPSDQLHIVMIP-LM-CPGHLIP
AcUGT73G1      1 -----------------------------------MNSNHHPLHVIIFP-FL-AQGHIIP
MtUGT73K1      1 -----------------------------------MGTESKPLKIYMLP-FF-AQGHLIP
GeUGT73F1      1 -------------------------------MDLEREGTEKPLKLYFIH-YL-AAGHMIP
AtUGT73B1      1 ---------------------------------MGTPVEVSKLHFLLFP-FM-AHGHMIP
GuUGAT         1 ---------------------------------MTMGNENRELHIIFFP-FL-ANGHIIP
NmUGT73BD1     1 --------------------------------------MTAQFHVVFFP-LM-AQGHLIP
```

```
NtTOGT1       1 ---------------------------------------MGQLHIFFFP-VM-AHGHMIP
BvUGT73A4     1 -----------------------------------MDDKSQQLHIVLFP-FM-AHGHMIP
Db5-GT        1 ---------------------------------MGTHSTAPDLHVVFFP-FL-AHGHMIP
AtUGT86A1     1 ----------------------------------MERAKSRKPHIMMIP-YP-LQGHVIP
ZmUFGT1       1 -------------------------------MAPADGESSPPPHVAVVA-FP-FSSHAAV
CteUGT78K6    1 --------------------------------------MKNKQHVAIFP-FP-FGSHLPP
AtUGT78D1     1 --------------------------------MTKFSEPIRDSHVAVLAFFP-VGAHAGP
CoUGT78B3     1 ---------------------------------MGQSKSESEAHVAVIA-FP-FGSHAAQ
MtUGT78G1     1 -----------------------------MSTFKNEMNGNNLLHVAVLA-FP-FGTHAAP
LgUGT78J1     1 --------------------------------------MVFESHIGVVA-FP-FGTHAAP
CpPGT11       1 ---------------------------------------MSSPHVVVIP-NP-EQGHVIP
AtUGT83A1     1 -------------------------------MDNNSNKRMGRPHVVVIP-YP-AQGHVLP
FaUGT75T1     1 -------------------------------------MESRNHHFLVIS-CA-AQGHLNP
AtUGT75C1     1 -------------------------------MATSVNGSHRRPHYLLVT-FP-AQGHINP
AtUGT75B1     1 ---------------------------------------MAPPHFLLVT-FP-AQGHVNP
AtUGT75D1     1 -------------------------------MANNNSNSPTGPHFLFVT-FP-AQGHINP
ZmUGT74A1     1 ----------------------------------------MAPHVLVVP-FP-GQGHMNP
AsUGT74H5     1 --------------------------------MGAEWEHVSDIHVLLLP-YP-VQGHINP
BdUGT74J7     1 ------------------------METNSPSSAEEGSGTGGGAHVLLLA-FPGAQGHLNP
SrUGT74G1     1 --------------------------------MAEQQKIKKSPHVLLIP-FP-LQGHINP
AtUGT74B1     1 ---------------------------------MAETTPKVKGHVVILP-YP-VQGHLNP
RsUGT74R1     1 -------------------------------------MATKKTQILILP-YP-IQGHINP
LuUGT74S1     1 ----------------------------------MAGDEREKAHCLVVP-AA-AQGHFTP
AtUGT74F1     1 -------------------------------------MEKMRGHVLAVP-FP-SQGHITP
SgUGT74AC1    1 -------------------------------------MEKGDTHILVFP-FP-SQGHINP
AtUGT74C1     1 ------------------------------------MSEAKKGHVLFFP-YP-LQGHINP
AtUGT74D1     1 ------------------------------------MGEKAKANVLVFS-FP-IQGHINP
AtUGT74E2     1 --------------------------------------MREGSHLIVLP-FP-GQGHITP
AtUGT84B1     1 ----------------------------------MGSSEGQETHVLMVT-LP-FQGHINP
DgpHBAGT      1 ---------------------------------MGSDQTLHHHHVLLVS-FP-SQGHVNP
MtUGT84F1     1 ------------------------------------MASEASIHILLVS-FP-AQGHINP
GtUF6CGT1     1 ---------------------------------MGSLTNNDNLHIFLVC-FI-GQGVVNP
PgUGT84A23    1 -----------------------------------MGSESSLVHVFLVS-FP-GQGHVNP
PgUGT84A24    1 ------------------------------------MGSESLVHVFLVS-FP-GQGHVNP
AtUGT76E1     1 -----------------------------------MEELGVKRRIVLVP-VP-AQGHVTP
AtUGT76D1     1 ------------------------------------MAEIRQRRVLMVP-AP-FQGHLPS
SrUGT76G1     1 ------------------------------MENKTETTVRRRRRIILFP-VP-FQGHINP
VvGT15        1 MGFHTRSSLRKSIPSMIVMLRERTGQKVKESHLSTLPRREMPRRVVLVP-CP-FQGHINP
AtUGT76C1     1 ------------------------------------MEKRNERQVILFP-LP-LQGCINP
AtUGT76B1     1 -----------------------------------METRETKPVIFLFP-FP-LQGHLNP
CsUGT76F1     1 ------------------------------------MDQRKGRRLVLFP-LP-LQGHISP
OsUGT709A4    1 -------------------------------------MGAAAAHVLVFP-MP-LQGHINV
SrUGT85C2     1 --------------------------------MDAMATTEKKPHVIFIP-FP-AQSHIKA
CoUGT85N1     1 ----------------------------------MASSSPKTPHIVCVP-AP-AQGHINP
MeUGT85K4     1 ---------------------------------MGSISPQKPPHAILVP-YP-AQGHVNP
AtUGT85A1     1 -------------------------------MGSQIIHNSQKPHVVCVP-YP-AQGHINP
MtUGT85H2     1 ----------------------------------MGNFANRKPHVVMIP-YP-VQGHINP
MtUGT71G1     1 ---------------------------------MSMSDINKNSELIFIP-AP-GIGHLAS
MdUGT71K1     1 ---------------------------------------MKKVELVFIP-SP-GAGHHLP
AtUGT89A2     1 -------------------------MTEVLLLPGTKSENSKPPHIVVFP-FP-AQGHLLP
AtUGT89C1     1 ----------------------------------MTTTTTKKPHVLVIP-FP-QSGHMVP
AtUGT89B1     1 ------------------------------MKVNEENNKPTKTHVLIFP-FP-AQGHMIP
NmUGT89P1     1 ----------------------------------MPSILSNSAHILLFP-FP-TSGHIIP
AtUGT92A1     1 ----------------------------------MAEAKPRNLRIVMFP-FM-GQGHIIP
GbUGT92K1     1 --------------------------------MAAGSQETVRMHLVMFP-YM-AQGHIIP
GmUGT92G4     1 -----------------------------------MAETPNKGHIVMVP-LM-AQGHLIP
PoUGT90A7     1 ------------------------------MASLLPDNSTHRPHFVLFP-FM-SKGHTIP
AtUGT90A1     1 -----------------------------------MSVSTHHHHVVLFP-FM-SKGHIIP
AcUGT73J1     1 -----------------------------------MAFDKKQLHILFFP-LM-SPGHFIP
MtUGT73P1     1 -----------------------------MESFGVKVEEETMLKAVFLP-FI-SKSHLIF
SrUGT73E1     1 -------------------------------MSPKMVAPPTNLHFVLFP-LM-AQGHLVP
CpPGT4        1 -----------------------------------MASEASQVHFLLLP-YL-APGHLIP
AtUGT73C1     1 ----------------------------------MASEFRPPLHFVLFP-FM-AQGHMIP
CpPGT2        1 -----------------------------------MDTQANQLHFVLFP-FL-AQGHMIP
PjGAT         1 -----------------------------MSQSPAMSKMQNQLHFVLVP-LL-AQGHMIP
CtiUGT73AE1   1 ----------------------------------MAPESASRIHFLLIP-LL-APGHTIP
CaUGT73AH1    1 -----------------------------------MDSQFQQLHFVMIP-LM-CPGHLIP
PzGAT2        1 -----------------------------------MDSPSDQLHIVMIP-LM-CPGHLIP
AcUGT73G1     1 -----------------------------------MNSNHHPLHVIIFP-FL-AQGHIIP
MtUGT73K1     1 -----------------------------------MGTESKPLKIYMLP-FF-AQGHLIP
GeUGT73F1     1 -------------------------------MDLEREGTEKPLKLYFIH-YL-AAGHMIP
AtUGT73B1     1 ---------------------------------MGTPVEVSKLHFLLFP-FM-AHGHMIP
GuUGAT        1 ---------------------------------MTMGNENRELHIIFFP-FL-ANGHIIP
NmUGT73BD1    1 --------------------------------------MTAQFHVVFFP-LM-AQGHLIP
```

```
AtUGT81A1     29 NRSPCSNSDGYALSSSNALYFNGFRTL--PSRRMG-KTLASLSFN-TKS----SAG--SS
AtUGT80A2     41 SGVVIV-SEELE--------T--------NPKTVV-ASIADETVA-ESS----GTG----
AtUGT80B1     20 VKSEKA-S--LL--------E------------TS-GSVDTTPED-SGH----RSS----
OsZOGT3       27 VLHLSL-Q--LA--------S--------SSHGLA-VHYAAPAPQ-LRQ----ARA----
OsZOGT1       25 LMHLSL----LL-----------------ASRGLD-VHYAAPPAH-LRQ-----------
ZmcisZOG1     25 LLHLSL----LL-----------------ASRGLS-VHYAAPPPH-VRQ-----------
GmUGT79A6     28 FVQLSNKL--FS--------H--------G---VH-VTFLSAASN-IPR-----------
AtUGT79B6     21 FLHLANKL---------------------AEKDHK-ITFLLPKKA-RKQ-----------
GmUGT91H9     19 YLELSK----IL-----------------AQKGHF-VTFISTPKNIDGM-----------
ZmUGT91L1     25 GLEFAERL---------------------AEHGQR-VSFVSTQGI-LRR-----------
PoUGT95A1     39 AMELCKNI---------------------SAHNYN-VTLIIPSHL-SSS-----------
MtUGT95B4     19 CFQLCNHL---------------------TSTNFH-VTLLISSTL-ATS-----------
PgUGT95B2     19 SMELCK-H--FA--------S---R----N---FR-AVLVISSNL-SSS-----------
VvUGT95B6     19 CIELCRHL---------------------ASRTFN-TTLIISSNL-SSS-----------
AtUGT82A1     23 MLHLASAF--LS--------R--------G---FS-PVVMTPESI-HRR----ISA----
VpUGT94F1     25 FLELAKTL---------------------SKRNFT-IHFCSTAIN-LDS-----------
AtUGT87A1     12 MLNLCKSL--VR-----------------RDPNLT-VTFVVTEEW---------------
AtUGT72C1     19 ILELGK-H--LL-----------------NHHGFDRVTVFLVTDD-VSR----SKS--LI
AtUGT72D1     20 ILELGN-R--LS-----------------SVLNIH-VTILAVTSG-SSS---------PT
LjUGT72AD1    23 ILEFCK-R--LA--------H--------LHQDIH-ITCINPTFE-SPC----SYV----
AtUGT72B1     23 LVEFAK-R--LV-----------------HLHGLT-VTFVIAGEG-PPS---------KA
VvUGT1        22 LIEFAR-R--LV-----------------LHHNFS-VTFLIPTDG-SPV---------TP
LjUGT72AH1    21 ILEFAK-R--LV--------H---L----HQDNFQ-VTCIIPTLS-NSP----TIA----
LjUGT72Z2     21 IIEFCKKL--LH-----------------LHHHYH-VTCIFPTID-APV---------AA
GmUGT72X4     21 IIEFSKRL--IK-----------------HHQNFH-VTCIVPSLG-PPP---------ES
MtUGT72L1     28 ILHFSKLL--IQ-----------------LHPDIH-VTCIIPTLG-SPP---------SS
PgUGT72BD1    24 VLQLGE-R--FV-----------------SRHNFV-VTVFVVVTD-AAT----AES--Q-
AtUGT72E1     13 VIELGK-R--LA-----------------GSHGFD-VTIFVLETD-AAS----AQS--QF
OsUGT706C1    22 MVQLAEAL--VR-----------------RGVSVT-LAVADPPDK-GAV----LAG---A
OsUGT706D1    23 MVELAKLF--LT-----------------RGLDVT-IAVPATPGS-GTT----GSP---T
MtUGT88E1     19 MVELGKLI--LT-----------------HHPSFS-IKILILTPP-NQDTNTINVSTSQY
VpUGT88D8     17 VLVIAK----FI-----------------NKHHPS-ISIIILSNAPDSA-----------
NmUGT88P1     20 MVELAKLI--LN-----------------REPSYS-IIIFISSAP-YST-----GSSAPY
AtUGT88A1     20 MVELGKTI--LS-----------------KNPSLS-IHIILVPPP-YQP-----ESTATY
VvGT7         18 MVELGKLI--LK-----------------HHPSFS-ITIFIVTPP-YNT-----GSTAPY
MdUGT88F1     19 MVELGKFI--VH-------RY--------GPHKFS-ITILYTCGS-IVD----TASIPVY
ScUGT5        19 TVELGK-L--IL-----------------RHHHFSTIHVLITTGF-DDS-----PHTATY
OsUGT707A2    26 MLEVGKRM--LR--------SRCCGDDDGRPAMS-LTVLLAQLP-ESHRAPEIDEIIRR
AtUGT71B1     19 TTALAK-L--LV--------A--------SDNRLS-VTLIVIPSR-VSD----DAS----
MdUGT71A15    21 TVEMAK-Q--LA--------A--------RDDQLF-ITVLVMKLP-YAQ-----------
BvUGT71F1     30 AVELAKLI--IQ-----------------RNHRIS-IVILIINIPTTTS----LINDFVH
Db6-GT        20 AVELSK-L--II--------R---RE---NRISVL-ILILSFPFD-SGL----VNA----
AtUGT71C1     23 TIELAKRL--IS--------Q--------DNPRIHTITILYWGLPFIPQ-----ADTIAF
AtUGT71C4     21 HIEFAK-R--LI--------N--------LDHRIHTITILNLSSP-SSP----HAS--VF
AtUGT71D1     20 FLEFAR-R--LI--------E--------QDDRIR-ITILLMKLQ-GQS----HLD--TY
FaUGT71W2     20 TIEFSK-R--LL--------D--------RCDQFS-VTILLMKSP-FGV----AAD----
MtUGT71G1     26 ALEFAK-L--LT--------N--------HDKNLY-ITVFCIKFP-GMP----FAD--SY
MdUGT71K1     20 TLQFVK-R--LI--------D--------RNDRIS-ITILAIQSY-FPT----TLS----
AtUGT89A2     34 LLDLTHQL--CL--------R--------G---FN-VSVIVTPGN-LTY-----------
AtUGT89C1     25 HLDLTHQI--LL-------------------RGAT-VTVLVTPKN-SSY---------LD
AtUGT89B1     29 LLDFTH-R--LA--------L---R----GGAALK-ITVLVTPKN-LPF-----------
NmUGT89P1     25 ILDLANQL--LA--------R--------G---LT-ITILITPAN-LTL----LST----
AtUGT92A1     25 FVALAL-R--LE------------KIMIMNRANKTTISMINTPSN-IPK-----------
GbUGT92K1     27 FIELSK-L--LA-----------------SRGGFT-ITIVNTPLN-IQR----LQSKIAL
GmUGT92G4     24 FLALAR-Q--IQ-----------------QNTSFT-ITIANTPQN-IQH----LRS----
PoUGT90A7     29 LLHLAK----LL-----------------ATRGIN-VTVFTTKAN-RPF-----------
AtUGT90A1     24 LLQFGR-L--LL--------R---HHR--KEPTIT-VTVFTTPKN-QPF----ISD----
AcUGT73J1     24 MIDMAC----IF-----------------ASHNVR-STVVATPSD-ASK-----------
MtUGT73P1     30 VVDIAR----LF-----------------AMHNVD-VTIITTPAN-AAI----FQT----
SrUGT73E1     28 MVDIAR-I--LA--------Q---R----G---AT-VTIITTPYH-ANR----VRP--VI
CpPGT4        24 MFDIAR-L--LA--------Q---H----G---AI-VTIVTTPVN-VAR----FKT--VI
AtUGT73C1     25 MVDIAR----LL-----------------AQRGVT-ITIVTTPQNAGRF-------KNVL
CpPGT2        24 MIDIAR----LL-----------------AQRGVI-ITIVTTPVN-AAR----FNG--IL
PjGAT         30 MIDMAR----LL-----------------AQHGVV-VSLVTTPHN-ASR----FAS----
CtiUGT73AE1   25 MIDIAK-L--LA-----------------QRPNVV-VTIVTTPVN-AAR----YGP--NL
CaUGT73AH1    24 LVDMAK----LL-----------------AQRAVT-VTIVTTPRN-AIR----FGA--AT
PzGAT2        24 MVDMAK----LL-----------------AQRAVT-VTIVATPRN-AIR----FGA--VI
AcUGT73G1     24 TIDLCK----LF-----------------ASHGVK-VTVLTTKGN-LSR----FHSPLTR
MtUGT73K1     24 LVNLAR----LV-----------------ASKNQH-VTIITTPSN-AQL----FDK----
GeUGT73F1     28 LCDIAT-L--FA--------S---R----G---HH-VTIITTPSN-AQT----LRR----
AtUGT73B1     26 TLDMAK----LF-----------------ATKGAK-STILTTPLNAKLF----FEKPIKS
GuUGAT        26 CVDLAR----VF-----------------AARGIR-ATIVTTHLN-VPY-----------
NmUGT73BD1    21 TLEIVK----LF-----------------SSRGLK-TTIVTTKLH-VPV-------ITKS
NmUGT73BD1     1 -----------------------------------MTAQFHVVFFP-LM-AQGHLIP
```

```
NtTOGT1      20 TLDMAK----LF-----------------ASRGVK-ATIITTPLNEFVF----------
BvUGT73A4    24 TLDIAR----LF-----------------AARGVK-TTLITTPRN-APT----FLT--AI
Db5-GT       26 SLDIAK----LF-----------------AARGVK-TTIITTPLN-ASM----FTKAIEK
AtUGT86A1    25 FVHLAIKL---------------------ASHGFT-ITFVNTDSI-HHHISTAHQDDAGD
ZmUFGT1      28 LLSIARALAAAA-----------------APSGAT-LSFLSTASS-LAQ----LRK--AS
CteUGT78K6   21 LLNLVL-K--LA-------H---------IAPNTS-FSFIGTHSS-NAF----------
AtUGT78D1    28 LLAVTR-R--LA-----------------AASPSTIFSFFNTARS-NAS----------
CoUGT78B3    26 ILNLTR-R--LA-------A---------SAPEVT-FSFFSTAKS-NKA----------
MtUGT78G1    30 LLSLVKKI--AT-----------------EAPKVT-FSFFCTTTT-NDT---------L
LgUGT78J1    21 LLDVVQ-R--IA-------A---------SAPGTL-FSFFNTADS-NRK----LFN----
CpPGT11      20 LLELSQNL---------------------AKHGLR-ITFVNSEYN-HNR----------
AtUGT83A1    28 LISFSR----YL-----------------AKQGIQ-ITFINTEFN-HNR----------
FaUGT75T1    22 SLQLAK-R--LI-----------------DIGSSH-VTFVTNIHG-LTQ----------
AtUGT75C1    28 ALQLANRL---------------------IHHGAT-VTYSTAVSA-HRR----------
AtUGT75B1    20 SLRFAR-R--LI-----------------KRTGAR-VTFVTCVSV-FHN----------
AtUGT75D1    28 SLELAK-R--LA-----------------GTISGARVTFAASISA-YNR----------
ZmUGT74A1    19 MVQFAK-R--LA-------S---K----G---VA-TTLVTTRFI-QRT----------
AsUGT74H5    27 MLQFGK-R--LA-------H---I----GGVGVR-CTLAITPYL-LRQ----------
BdUGT74J7    36 LLQFGRRL---------------------AYHGLR-PTFVTTRYL-LST----------
SrUGT74G1    27 FIQFGK-R--LI-------S---K----G---VK-TTLVTTIHT-LNS----------
AtUGT74B1    26 MVQFAKRL---------------------VSKNVK-VTIATTTYT-ASS----------
RsUGT74R1    22 MLQFAKRL--AS-----------------KSRHLI-LTLLLPTSH--------------
LuUGT74S1    25 LLQFSK-R--LI-------P---K----R---IR-VTLALTRFI-HST----------
AtUGT74F1    22 IRQFCK-R--LH-------S---K----G---FK-TTHTLTTFI-FNT----------
SgUGT74AC1   22 LLQLSKRL--IA-------------------KGIK-VSLVTTLHV-SNH----------
AtUGT74C1    23 MIQLAKRL---------------------SKKGIT-STLIIASKD-HRE----------
AtUGT74D1    23 LLQFSKRL--LS-------------------KNVN-VTFLTTSST-HNS--------IL
AtUGT74E2    21 MSQFCKRL---------------------ASKGLK-LTLVLVSDK-PSP----------
AtUGT84B1    25 MLKLAK-H--LS-------L---------SSKNLH-INLATIESA-RDL----------
DgpHBAGT     26 LLRLAKHL---------------------ASKGLL-ATFSSTHHIIHKI----------
MtUGT84F1    23 LLRLGKCL---------------------AAKGAS-VIFITTEKG-GKN----MRITNKL
GtUF6CGT1    26 MLRLGKAF---------------------ASKGLL-VTLSAPEIVGTEI--------RK
PgUGT84A23   24 LLRLGKRL---------------------ASKGLL-VTFTTPESI-GKQ--------MR
PgUGT84A24   23 LLRLGKRL---------------------ASKGLL-VTFTTPESI-GKQ--------MR
AtUGT76E1    24 IMQLGKAL---------------------YSKGFS-ITVVLTQYN--------------
AtUGT76D1    23 MMNLASYL---------------------SSQGFS-ITIVRNEFN--------------
SrUGT76G1    29 ILQLAN----VL-----------------YSKGFS-ITIFHTNFN-KPK----------
VvGT15       59 MLQLGA----IL-----------------HSRGFS-ITVAHTQYN-SPD----------
AtUGT76C1    23 MLQLAK----IL-----------------YSRGFS-ITIIHTRFN-APK----------
AtUGT76B1    24 MFQLAN----IF-----------------FNRGFS-ITVIHTKFN-SPN----------
CsUGT76F1    23 MLQLAN----IL-----------------HSQGFT-ITIIHTSFN-SPN----------
OsUGT709A4   22 MLHLAT-A--LA-----------------AAAGVH-VTFLHTDHN-LHR-------LGNA
SrUGT85C2    27 MLKLAQ----LL-----------------HHKGLQ-ITFVNTDFI-HNQ----FLE--SS
CoUGT85N1    25 MFKLAK-L--FH-------S---R----G---FY-ITFVHSEFS-YQR----LLQ--AS
MeUGT85K4    26 LMQLGK----LL-----------------HARGFY-ITFVNTEHN-HRR----LIR--SR
AtUGT85A1    28 MMRVAK----LL-----------------HARGFY-VTFVNTVYN-HNR----FLR--SR
MtUGT85H2    25 LFKLAK----LL-----------------HLRGFH-ITFVNTEYN-HKR----LLK----
MdUGT71K1    20 TLQFVK-R--LI-------D---------RNDRIS-ITILAIQSY-FPT----TLS----
AtUGT89A2    34 LLDLTHQL--CL-------R---------G---FN-VSVIVTPGN-LTY----------
AtUGT89C1    25 HLDLTHQI--LL-------------------RGAT-VTVLVTPKN-SSY--------LD
AtUGT89B1    29 LLDFTH-R--LA-------L---R----GGAALK-ITVLVTPKN-LPF----------
NmUGT89P1    25 ILDLANQL--LA-------R---------G---LT-ITILITPAN-LTL----LST----
AtUGT92A1    25 FVALAL-R--LE-----------KIMIMNRANKTTISMINTPSN-IPK----------
GbUGT92K1    27 FIELSK-L--LA-----------------SRGGFT-ITIVNTPLN-IQR----LQSKIAL
GmUGT92G4    24 FLALAR-Q--IQ-----------------QNTSFT-ITIANTPQN-IQH----LRS----
PoUGT90A7    29 LLHLAK----LL-----------------ATRGIN-VTVFTTKAN-RPF----------
AtUGT90A1    24 LLQFGR-L--LL-------R---HHR--KEPTIT-VTVFTTPKN-QPF----ISD----
AcUGT73J1    24 MIDMAC----IF-----------------ASHNVR-STVVATPSD-ASK----------
MtUGT73P1    30 VVDIAR----LF-----------------AMHNVD-VTIITTPAN-AAI----FQT----
SrUGT73E1    28 MVDIAR-I--LA-------Q---R----G---AT-VTIITTPYH-ANR----VRP--VI
CpPGT4       24 MFDIAR-L--LA-------Q---H----G---AI-VTIVTTPVN-VAR----FKT--VI
AtUGT73C1    25 MVDIAR----LL-----------------AQRGVT-ITIVTTPQNAGRF-------KNVL
CpPGT2       24 MIDIAR----LL-----------------AQRGVI-ITIVTTPVN-AAR----FNG--IL
PjGAT        30 MIDMAR----LL-----------------AQHGVV-VSLVTTPHN-ASR----FAS----
CtiUGT73AE1  25 MIDIAK-L--LA-----------------QRPNVV-VTIVTTPVN-AAR----YGP--NL
CaUGT73AH1   24 LVDMAK----LL-----------------AQRAVT-VTIVTTPRN-AIR----FGA--AT
PzGAT2       24 MVDMAK----LL-----------------AQRAVT-VTIVATPRN-AIR----FGA--VI
AcUGT73G1    24 TIDLCK----LF-----------------ASHGVK-VTVLTTKGN-LSR----FHSPLTR
MtUGT73K1    24 LVNLAR----LV-----------------ASKNQH-VTIITTPSN-AQL----FDK----
GeUGT73F1    28 LCDIAT-L--FA-------S---R----G---HH-VTIITTPSN-AQT----LRR----
AtUGT73B1    26 TLDMAK----LF-----------------ATKGAK-STILTTPLNAKLF----FEKPIKS
GuUGAT       26 CVDLAR----VF-----------------AARGIR-ATIVTTHLN-VPY----------
NmUGT73BD1   21 TLEIVK----LF-----------------SSRGLK-TTIVTTKLH-VPV-------ITKS
NmUGT73BD1    1 ----------------------------------MTAQFHVVFFP-LM-AQGHLIP
```

```
AtUGT81A1     79 LRRFISDFNSF-------------IRFHCDKVVPE-----SFASVGGVGLSSDENGIREN
AtUGT80A2     74 NKSFSRVWTMP--LEGSSSSDKAESSSTNQPRLDK-----SKT--ERQQKVTH-------
AtUGT80B1     47 DGHRGLDHCETAPVGLYGDMLINDSEIQYSRSLTE----KGSPAIHNLKLDRLSEQE---
OsZOGT3       58 RVHGWDDKALLS------------VQFHDL-GISTYVSPPPDP--TADTPFPSHLMPL--
OsZOGT1       51 ARLRLHGWDPDALRS---------IRFHDL-DVPA---YESPPPDPTAPPFPSHLMPM--
ZmcisZOG1     51 ARARVHGWDPRALGS---------IRFHDL-DVPP--YDSPAPDLAAPSPFPNHLMPM--
GmUGT79A6     54 IRSTLNLNPA--------------INVISL-KFPN-----------GITNTAELPPH---
AtUGT79B6     47 -LESLNLFPDC-------------IVFQTL-TIPS---VDGLP--DGAETTSDIPIS---
GmUGT91H9     46 PKIPETLQPS--------------IKLVRL-PLPHTDHHHHLP--EDAESTMDIPSN---
ZmUGT91L1     51 LRPVAPALASL-------------IDLVAL-PFPR---IDGLP--DGAEATSDLPPG---
PoUGT95A1     65 IPSTFSNHSSF-------------IHVTEISVAAS----------PPPEAADEPGSGTEV
MtUGT95B4     45 IPSSLHQHPL--------------FQVTLI---------------PSQPPPPSPEHH---
PgUGT95B2     45 VPSSLRSLPL--------------VEVVEI-PSSP------TP--PPPPPPSQPGSGD--
VvUGT95B6     45 IPSDLRRIPL----------------FHIF-EISS-----SLP--PPPPPSSPSPDSD--
AtUGT82A1     52 TNED--------------------LGITFL-ALSD-----GQD--RPDAPPSD-------
VpUGT94F1     51 IKSNLANDPSVLDDS---------IKLLEL-EIES----PELP--PELHTTKNLPPH---
AtUGT87A1     37 -LGFIGSDPKP-------------NRIHFA-TLPN-----IIP--SELVRANDFIAF---
AtUGT72C1     52 GKTLMEEDPK--------------FVIRFI-PLDV----------SGQDLSGSLLTK---
AtUGT72D1     49 ETEAIHAAAA--------------RTICQITEIPS----------VDVDNLVEPDAT---
LjUGT72AD1    54 VKALFENLPSN-------------IECMFLPPVNL----------DDMAQTSDPAIL---
AtUGT72B1     52 QRTVLDSLPSS-------------ISSVFLPPVDL----------TDLSSSTRIESR---
VvUGT1        51 QKSVLKALPTS-------------INYVFLPPVAF----------DDLPEDVRIETR---
LjUGT72AH1    53 SKPFFNALPTS-------------IQCIFL-PPVN------VK--NGNDPLETQ------
LjUGT72Z2     51 TLKLLQSLPST-------------IDFKFLPPVNK----------QDLPQDVSPAVQ---
GmUGT72X4     51 SKAYLKTLPSN-------------IDTILLPPISK----------EQLPQGVHPAIL---
MtUGT72L1     58 SETILQTLPSN-------------IDYMFL-PEVQ----------PSDLPQGLPMEIQ--
PgUGT72BD1    55 LKQQSSYKNL--------------LNVVLLPPVDL----------TSLTNKPLAILSQ--
AtUGT72E1     45 LNSPGCDAAL--------------VDIVGL-PTPD---------ISGLVDPSAFFGIK--
OsUGT706C1    54 IARIAAVCPS--------------IGVRLL-PIPS----------CEGKTYSHPVMW---
OsUGT706D1    55 IAGIAASNPS--------------ITFHHL-PPPP----------SCADPDNPLLL---
MtUGT88E1     58 ISSVSNKFPS--------------INFHYI---------------PSISFTFTLPPHLQ-
VpUGT88D8     44 ASSITSEASS--------------ITYHRL-PTPD----------IPPNIITNPVEL---
NmUGT88P1     54 ISHVSATTSG--------------ISFHHL-PVLV------LP--PNTFSSFEEIAYK--
AtUGT88A1     54 ISSVSSSFPS--------------ITFHHL-PAVT-------PYSSSSTSRHHHESL---
VvGT7         52 LARVSSTIPS--------------ITFHHL-PTIS------LP--LDSFSSPNHETL---
MdUGT88F1     56 IRRISHSHPF--------------ISFRQF-PRVT----------NNITRNISVPAI---
ScUGT5        53 IDQISKTNPS--------------ITFHRL-PFLP----------LNLSPTVSSIAT---
OsUGT707A2    74 EAAGASEHSG--------------FDVRFH-CLPA----------EELPDFRGGEDF---
AtUGT71B1     50 SSVYTNSEDR--------------LRYILL---------------PARDQTTD-------
MdUGT71A15    49 PFTNTDSSIS--------------HRINFV-NLPE-----AQP--DKQDIVPNPGSF---
BvUGT71F1     66 SQSRNNPYPTH-------------LTFMTLPPLSN----------PPERSSPDF------
Db6-GT        53 YVDFQSRDPDNS------------GSLTFI-TLPP------LSNIPDCTSSTF-------
AtUGT71C1     60 LRSLVKNEPR--------------IRLVTLPEVQD-------P--PPMELFVEFAESY--
AtUGT71C4     55 ARSLIASQPK--------------IRLHDLPPIQD-------P--PPFDLYQRAPEAY--
AtUGT71D1     53 VKSIASSQPF--------------VRFIDVPELEE-------K--PTLGSTQSVEAY---
FaUGT71W2     51 --QSLPAASN--------------TNIKLI-HLPN-------I--NPPIKLDSVEKF---
MtUGT71G1     59 IKSVLASQPQ--------------IQLIDL-PEVE-------P--PPQELLKSPEFY---
MdUGT71K1     51 SYTKSIAASEPR------------IRFIDV-PQPQ----DRPP--QEMYKSRAQI-----
AtUGT89A2     60 LSPLLSAHPSS-------------VTSVVF-PFPP---HPSLS--PGVENVKDVGNS---
AtUGT89C1     53 ALRSLHSPEH--------------FKTLIL-PFPS---HPCIP--SGVESLQQLPLE---
AtUGT89B1     58 LSPLLSAVVN--------------IEPLIL-PFPS---HPSIP--SGVENVQDLPPS---
NmUGT89P1     54 QLIELDRLGS--------------LHTLVL-PFPN----------PPNPSETS-------
AtUGT92A1     58 IRSNLPPESS--------------ISLIEL-PFNS--SDHGLP--HDGENFDSLPYS---
GbUGT92K1     61 LRLQAENLD---------------IRLAEL-PFDG--TSHGLP--PNTESTESLPHS---
GmUGT92G4     54 ALSSSTSPNHQ-------------IHLAELVPFNS--TQHSNK--DN--NTQKAPLT---
PoUGT90A7     55 IAQFLHRHSNS-------------VSIIDL-PFPR--DVEGIP--QGIESTDKLPSMS--
AtUGT90A1     58 FLSDTPE-----------------IKVISL-PFPE--NITGIP--PGVENTEKLPSMS--
AcUGT73J1     50 IPLSKSKY----------------ISVVTI-PFPS-PSLTNLP--PDHENLATIRSS---
MtUGT73P1     59 SIDHDSSRGRS-------------IRTHIV-KFPQ---VPGLP--QGMESFNADTPKD--
SrUGT73E1     59 SRAIATN-----------------LKIQLL-ELQLRSTEAGLP--EGCESFDQLPSFE--
CpPGT4        55 ARAIKSGLQ---------------IRLIEI-QFPW--QEAGIP--EGCENCDLLPTT---
AtUGT73C1     56 SRAIQSGLP---------------INLVQV-KFPS--QESGSP--EGQENLDLLDSLG--
CpPGT2        55 SRAIESGLQ---------------IKIVQF-QLPC--EEAGLP--EGCENLDMVASLG--
PjGAT         59 TIHRARDSGLK-------------IQLIQI-PFPC--QEVGLP--PGCENLDSVPSRD--
CtiUGT73AE1   57 QPHIDSGLP---------------VRFLEL-PFPA--AEAGLP--AGIESADSLPGLH--
CaUGT73AH1    55 SRAIQSGLP---------------IRLLEL-RFPT--LEAGLP--EGCENVDQLSSFD--
PzGAT2        55 GRAIESGLP---------------IRLLEV-RFPA--LEAGLP--EGCESVDDLPSLA--
AcUGT73G1     57 ANELSTFLHP--------------IQISLI-PFPS---VSGLP--ENCENMATVPPH---
MtUGT73K1     53 TIEEEKAAGHH-------------IRVHII-KFPS--AQLGLP--TGVENLFAASDNQ--
GeUGT73F1     57 SIPFNDYHQ---------------LCLHTV-PFPS--QEVGLP--DGVESLSSVTDLD--
AtUGT73B1     60 FNQDNPGLED--------------ITIQIL-NFPC--TELGLP--DGCENTDFIFSTPDL
GuUGAT        52 ISRTIGKAN---------------INIRTI-KFPS-TEDSGLP--EGCENTESALAPD--
NmUGT73BD1    51 IEKTNHSGNQ--------------INISII-KFPC--KEVGLP--EGSESLDTLKQPD--
NmUGT73BD1     1 --------------------------------------MTAQFHVVFFP-LM-AQGHLIP
```

```
NtTOGT1       47  SKAIQRNKHLGIE-----------IEIRLI-KFPA--VENGLP--EECERLDQIPSDE--
BvUGT73A4     55  EKGNKSGAPT--------------INVEVF-NFQA--QSFGLP--EGCENLEQALGPG--
Db5-GT        59  TRKNTETQ----------------MEIEVF-SFPS--EEAGLP--LGCENLEQAMAIG--
AtUGT86A1     62  IFSAARSSGQ--------------HDIRYT-TVSD-----GFP--LDFDRSLNHDQF---
ZmUFGT1       63  SASAGHGLP---------------GNLRFV-EVPD-----GAPAAEESVPVPRQMQLF--
CteUGT78K6    49  --LFTKRHIP--------------NNIRVF-TISD-----GIP--EGHVPANNPIEK---
AtUGT78D1     56  --LFSSDHPEN-------------IKVHDV---SD-----GVP--EGTMLGNPLEM----
CoUGT78B3     54  --VSGSTGGA--------------ENIKFY-DVHH-----GVP--ENHSFSGNPLEE---
MtUGT78G1     59  FSRSNEFLPN--------------IKYYNV---HD-----GLP--KGYVSSGNPREP---
LgUGT78J1     52  --TC----AN--------------IRIHEV---WD-----GTP--RDQVFTGSHFEA---
CpPGT11       46  VLESLEGKNYIG------------EQIHLV-SIPD-----GIEPWDDRSDMRKL------
AtUGT83A1     54  IISSLPNSPHEDYVG---------DQINLV-SIPD-----GLE--DSPEERNIPGKL---
FaUGT75T1     49  ----IKSLPS--------------LEGLSFASFSD-----GFD--DGVHPANDPEHI---
AtUGT75C1     54  ----MGEPPS--------------TKGLSFAWFTD-----GFD--DGLKSFEDQKIY---
AtUGT75B1     47  --SMIANHNKV-------------ENLSFL-TFSD-----GFD--DGGISTYEDRQKR--
AtUGT75D1     56  RMFSTENVP---------------ETLIFA-TYSD-----GHD--DGFKSSAYSDKSRQD
ZmUGT74A1     45  --ADVDAHPA------------------MVEAISD-----GHD--EGGFASAAGVAEY--
AsUGT74H5     56  -----CQDPCP-------------GAVHLV-EISD-----GFD--SAGFEEVGDVAAY--
BdUGT74J7     62  -----VPPPA--------------GPFRVA-AISD-----GFDAGGMAACSTGFGDY---
SrUGT74G1     53  --TLNHSNTT--------------TTSIEIQAISD-----GCD--EGGFMSAGESY----
AtUGT74B1     52  -----ITTPS--------------LSVE---PISD-----GFDFIPIGIPGFSVDTY---
RsUGT74R1     47  ARSISSHIGS--------------INVQ---PISD-----GAD--QQGQQFQTAETY---
LuUGT74S1     51  --MTVTAQSG--------------IHIDTI---SD-----GFD--HSGLILQDPEHY---
AtUGT74F1     48  ------------------------IHLDPSSPISIATISDGYD--QGGFSSAGSVPEY--
SgUGT74AC1    48  LQLQGAYSNS--------------VKIEVI-SDGS----------EDRLETDTMRQT---
AtUGT74C1     49  --PYTSDDYS--------------ITVHTI---HD-----GFF--PHEHPHAKFVD----
AtUGT74D1     51  RRAITGGATA--------------LPLSFV-PIDD-----GFE--EDHPSTDTSPDY---
AtUGT74E2     47  --PYKTEH----------------DSITVF-PISN-----GFQ--EGEEPLQDLDDY---
AtUGT84B1     53  LSTVEKPRYP--------------VDLVFF---SD-----GLP--KEDPKAPETL-----
DgpHBAGT      53  HKSTGHVQPIDIGG----------GRLRFE-SFTD-----GWE--DHDDPRRSDLNVY--
MtUGT84F1     56  ATPIGDGS----------------LMFQFF----D----------DGLPDYAHPLDH---
GtUF6CGT1     55  ANNLNDDQPIKVGSGM--------IRFEFF---DD-----GWESVNGSKPFDVWVY----
PgUGT84A23    52  KASNISDQPAPVGDGF--------IRFEFF---ED-----GWD--EDEPRRQDLDQY---
PgUGT84A24    51  KASNIGEEPSPIGDGF--------IRFEFF---ED-----GWD--EDEPRRQDLDQY---
AtUGT76E1     47  --RVSSSKDF--------------SDFHFL-TIPG-----SLT--ESDLKNLGPFKF---
AtUGT76D1     46  FKDISHNFPG--------------IKFFTI---KD-----GLS--ESDVKSLGLLEF---
SrUGT76G1     55  ----TSNYPH--------------FTFRFI-LDND----------PQDERISNLPTHGPL
VvGT15        85  ----PSNHPD----------------FSFL-PIPD-----GL---SDGQNFASLLNL---
AtUGT76C1     49  ----SSDHPL----------------FTFL-QIRD-----GLS--ESQTQSRDLLLQ---
AtUGT76B1     50  ----SSNFPH----------------FSFV-SIPD-----GL---SEPESYPDVIEI---
CsUGT76F1     49  ----PSSHPH--------------LTFHFIQENLS----------ASEASTDDLVAF---
OsUGT709A4    53  AAATTAGSP---------------RRLRFL-SVPD-----GLP--DDHPRSASDVPVM--
SrUGT85C2     58  GPHCLDGAPG--------------FRFETI---PD-----GVS--HSPEASIPIRESL--
CoUGT85N1     56  ALDHLKGL----------------NNFRFE-TIPD-----GLP--PENKRGVSDVPEL--
MeUGT85K4     57  GQEFIDGLPD--------------FKFEAI---PD-----GLPY-TDRDATQHVPSL---
AtUGT85A1     59  GSNALDGLPS--------------FRFESI---AD-----GLP--ETDMDATQDITAL--
MtUGT85H2     54  SRGPKAFDGF--------------TDFNFE-SIPD-----GLTPMEGDGDVSQDVPTL--
MdUGT71K1     51  SYTKSIAASEPR------------IRFIDV-PQPQ----DRPP--QEMYKSRAQI-----
AtUGT89A2     60  LSPLLSAHPSS-------------VTSVVF-PFPP---HPSLS--PGVENVKDVGNS---
AtUGT89C1     53  ALRSLHSPEH--------------FKTLIL-PFPS---HPCIP--SGVESLQQLPLE---
AtUGT89B1     58  LSPLLSAVVN--------------IEPLIL-PFPS---HPSIP--SGVENVQDLPPS---
NmUGT89P1     54  QLIELDRLGS--------------LHTLVL-PFPN----------PPNPSETS-------
AtUGT92A1     58  IRSNLPPESS--------------ISLIEL-PFNS--SDHGLP--HDGENFDSLPYS---
GbUGT92K1     61  LRLQAENLD---------------IRLAEL-PFDG--TSHGLP--PNTESTESLPHS---
GmUGT92G4     54  ALSSSTSPNHQ-------------IHLAELVPFNS--TQHSNK--DN--NTQKAPLT---
PoUGT90A7     55  IAQFLHRHSNS-------------VSIIDL-PFPR--DVEGIP--QGIESTDKLPSMS--
AtUGT90A1     58  FLSDTPE-----------------IKVISL-PFPE--NITGIP--PGVENTEKLPSMS--
AcUGT73J1     50  IPLSKSKY----------------ISVVTI-PFPS-PSLTNLP--PDHENLATIRSS---
MtUGT73P1     59  SIDHDSSRGRS-------------IRTHIV-KFPQ---VPGLP--QGMESFNADTPKD--
SrUGT73E1     59  SRAIATN-----------------LKIQLL-ELQLRSTEAGLP--EGCESFDQLPSFE--
CpPGT4        55  ARAIKSGLQ---------------IRLIEI-QFPW--QEAGIP--EGCENCDLLPTT---
AtUGT73C1     56  SRAIQSGLP---------------INLVQV-KFPS--QESGSP--EGQENLDLLDSLG--
CpPGT2        55  SRAIESGLQ---------------IKIVQF-QLPC--EEAGLP--EGCENLDMVASLG--
PjGAT         59  TIHRARDSGLK-------------IQLIQI-PFPC--QEVGLP--PGCENLDSVPSRD--
CtiUGT73AE1   57  QPHIDSGLP---------------VRFLEL-PFPA--AEAGLP--AGIESADSLPGLH--
CaUGT73AH1    55  SRAIQSGLP---------------IRLLEL-RFPT--LEAGLP--EGCENVDQLSSFD--
PzGAT2        55  GRAIESGLP---------------IRLLEV-RFPA--LEAGLP--EGCESVDDLPSLA--
AcUGT73G1     57  ANELSTFLHP--------------IQISLI-PFPS---VSGLP--ENCENMATVPPH---
MtUGT73K1     53  TIEEEKAAGHH-------------IRVHII-KFPS--AQLGLP--TGVENLFAASDNQ--
GeUGT73F1     57  SIPFNDYHQ---------------LCLHTV-PFPS--QEVGLP--DGVESLSSVTDLD--
AtUGT73B1     60  FNQDNPGLED--------------ITIQIL-NFPC--TELGLP--DGCENTDFIFSTPDL
GuUGAT        52  ISRTIGKAN---------------INIRTI-KFPS-TEDSGLP--EGCENTESALAPD--
NmUGT73BD1    51  IEKTNHSGNQ--------------INISII-KFPC--KEVGLP--EGSESLDTLKQPD--
NmUGT73BD1     1  -------------------------------------MTAQFHVVFFP-LM-AQGHLIP
```

```
AtUGT81A1     121 GTGGVLGEEGLPLNGVEADRPKKVLILMSDTGGGHRASAEAIRAAFNQEFGDEYQVFITD
AtUGT80A2     118 -----ILAEDAAKIFDDKISAGKKLKLLNRIATVKHDGTVEFEVPADAI----PQPIVVD
AtUGT80B1     100 -----KQKL-------IVELVRIQNDGTVEVIDNGTPVSELWEFEPTKG----QSTITYE
OsZOGT3       101 -----WEAY-------TADARAPLSALLDEL-------------SASHR----RVVVVCD
OsZOGT1        96 -----LESF-------AVAARVPLAALLERL-------------SASYR----RVVVVYD
ZmcisZOG1      97 -----FEAF-------AAAARAPLAALLQRL-------------STSYR----RVAVVFD
GmUGT79A6      85 -----LAGN-------LIHALDLTQDQVKSL-------------LLELK----PHYVFFD
AtUGT79B6      84 -----LGSF-------LASAMDRTRIQVKEA-------------VSVGK----PDLIFFD
GmUGT91H9      86 -----KSYY-------LKLAYEALQGPVSEL-------------LKTSK----PDWVFYD
ZmUGT91L1      89 -----TAEL-------HVQAMDRLAPAFSAFLGAACAD------GSDRK----VDWVLLD
PoUGT95A1     102 RSSGPRGNP-------LQDQNLQMGKGIKSFLSA----------RSGTR----PTCVVID
MtUGT95B4      73 ----------------HDELTKGLQNIFSNY-------------PRPTR----PVCAIVD
PgUGT95B2      80 -----LMAQ-------HRNHHSQMAQRLESVLSARASA------PNSIP----IACAVTD
VvUGT95B6      79 -----PMSH-------HHPHHQQMGTAIESLLSS----------RSTSPDYVPPLCAIID
AtUGT82A1      77 -----FFSI-------ENSMENIMPPQLERLLL-----------EEDLD----VACVVVD
VpUGT94F1      92 -----QFPL-------LIKDFENSKSSFFSI-------------FDTLK----PDMLIYD
AtUGT87A1      72 ----------------IDAVLTRLEEPFEQLLD-----------RLNSP----PTAIIAD
AtUGT72C1      84 -----LAEM-------MRKALPEIKSSVMEL---------------EPR----PRVFVVD
AtUGT72D1      82 -----IFTK-------MVVKMRAMKPAVRDAVK-----------LMKRK----PTVMIVD
LjUGT72AD1     88 -----IQAT-------IIRSLPSIYNVLNTLHCS----------SNSRG----LASIVVD
AtUGT72B1      86 -----ISLT-------VTRSNPELRKVFDSFV------------EGGRL----PTALVVD
VvUGT1         85 -----ISLS-------MTRSVPALRDSLRTL-------------TESTR----LVALVVD
LjUGT72AH1     85 -----LQIA-------ISDAMPSVREALRSL-------------ASSSK----VVALVVD
LjUGT72Z2      85 -----IQLA-------VSQSMPSFRDTMRSLH------------STTPH----LTALIAD
GmUGT72X4      85 -----IQLT-------ITLSLPSIHEALKSL-------------CSKAP----LTALVVD
MtUGT72L1      92 -----IQLT-------VTNSLPYLHEALKSL-------------ALRIP----LVALVVD
PgUGT72BD1     89 -----LVLI-------VRQSLPDLRSAIASM---------------KSR----PTALIVD
AtUGT72E1      79 -----LLVM-------MRETIPTIRSKIEEM---------------QHK----PTALIVD
OsUGT706C1     86 -----IVDA-------LRLANPVLRELLRSF-------------PAA------VDALVVD
OsUGT706D1     87 -----MLDV-------LRRSVPSLASLLRSI----------------PS----VAALVLD
MtUGT88E1      88 -----TLEL-------SPRSNHHVHHILQSI-------------AKTSN----LKAVMLD
VpUGT88D8      76 -----LFEV-------PRLNNPNVKQYLEQI-------------SQKTN----VKAFIID
NmUGT88P1      89 -----IPQL----------NNPNLKLALQTIS------------KESSD----LKAFIID
AtUGT88A1      89 -----LLEI-------LCFSNPSVHRTLFSL-------------SRNFN----VRAMIID
VvGT7          86 -----AFEL-------LRLNNPNIHQALVSI-------------SNNSS----VRALIVD
MdUGT88F1      88 -----TFDF-------IRQNDPHVRSALQEI-------------SKSAT----VRAFIID
ScUGT5         85 -----LFEF-------IRLNATSVLHTLQKI-------------LQTSK----VRALILD
OsUGT707A2    106 -----ISRF-------MQQHASHAREAIAGL---------------ESR----VAAVVLD
AtUGT71B1      74 -----LVSY-------IDSQKPQVRAVVSKVAGDVST-------RSDSR----LAGIVVD
MdUGT71A15     84 -----FRMF-------VENHKSHVRDAVINVLPESDQSES----TSKPR----LAGFVLD
BvUGT71F1      97 -----MRIL-------IELHEPLVKQAVEERIR-----------AGSSK----LAGFVLD
Db6-GT         87 -----FTTV-------IELHKPNVKQVVEERVR-----------SGSPK----PAGFVID
AtUGT71C1      95 -----ILEY-------VKKMVPIIREALSTLLSSRDE-------SGSVR----VAGLVLD
AtUGT71C4      90 -----IVKL-------IKKNTPLIKDAVSSIVASRRGG------SDSVQ----VAGLVLD
AtUGT71D1      87 -----VYDV-------IERNIPLVRNIVMDILTSLA--------LDGVK----VKGLVVD
FaUGT71W2      82 -----LSDY-------IETYKHHVKDTILNQVL-----------PNSSR----IAGVVID
MtUGT71G1      92 -----ILTF-------LESLIPHVKATIKTILSNK------------------VVGLVLD
MdUGT71K1      87 -----FSLY-------IESHVPSVKKIITNLVSSSANS------SDSIR----VAALVVD
AtUGT89A2      98 -----GNLP-------IMASLRQLREPIINWFQ-----------SHPNP----PIALISD
AtUGT89C1      90 -----AIVH-------MFDALSRLHDPLVDF-------------LSRQPPSDLPDAILGS
AtUGT89B1      95 -----GFPL-------MIHALGNLHAPLISWIT-----------SHPSP----PVAIVSD
NmUGT89P1      82 -----LAAR-------VHASSQ-LSNTIIQWFQ-----------SHTSP----PVAIVSD
AtUGT92A1      96 -----LVIS-------LLEASRSLREPFRDFMTKILK-------EEGQS----SVIVIGD
GbUGT92K1      98 -----LFLP-------LLQASQQLEQPFQQLVQDIVR-------KEGRL----PACIISD
GmUGT92G4      92 -----DLLK-------LGYASLTLEPPFRSLISQITE-------EDGHP----PLCIISD
PoUGT90A7      95 -----FFPK-------FATATKLMQPDFEQAL------------EKIPD----VTCIVSD
AtUGT90A1      94 -----LFVP-------FTRATKLLQPFFEETL------------KTLPK----VSFMVSD
AcUGT73J1      87 -----MFDL-------FVSALSLFQPPLQNL-------------IHDLK----PDCLISD
MtUGT73P1      98 -----IISK-------IYQGLAILQEQFTQL-------------FRDMK----PDFIVTD
SrUGT73E1      97 -----YWKN-------ISTAIDLLQQPAEDLLR-----------ELSPP----PDCIISD
CpPGT4         92 -----DFAR-------FMKSLHMLQQPFENLFK-----------EKTLK----PCCIISD
AtUGT73C1      94 -----ASLT-------FFKAFSLLEEPVEKLLK-----------EIQPR----PNCIIAD
CpPGT2         93 -----LAFD-------FFTAADMLQEPVENVFA-----------QLKPR----PNCIISD
PjGAT          99 -----LIGN-------FFSALNKLQQPLEQHLQ-----------ELMPP----PSCIISD
CtiUGT73AE1    95 -----LLQN-------FSLAVDMLQQRLEQRFE-----------SLEPR----PSCILSD
CaUGT73AH1     93 -----FSKN-------FIDASKMQQGPLEKIFG-----------DIKPS----PSCIISD
PzGAT2         93 -----MSIN-------FFAATKMLQEPVEKMLK-----------DIKPS----PSCILSD
AcUGT73G1      94 -----LKSL-------FFDAVAMLQQPFRAF-------------LKETN----PDCVVAG
MtUGT73K1      93 -----TAGK-------IHMAAHFVKADIEEF-------------MKENP----PDVFISD
GeUGT73F1      95 -----NLAK-------VFQATTLLRTPIEHF-------------VEENP----PDCIVAD
AtUGT73B1     101 NVGD-LSQK-------FLLAMKYFEEPLEEL-------------LVTMR----PDCLVGN
GuUGAT         91 -----KFIK-------FMKATLLLRDPLEHV-------------LQEEQ----PHCLVAD
NmUGT73BD1     90 -----MFMK-------FFRGLSLLQEPFEQI-------------LQELS----PDCLVAD
NmUGT73BD1      1 --------------------------------------MTAQFHVVFFP-LM-AQGHLIP
```

```
NtTOGT1       89 -----KLPN-------FFKAVAMMQEPLEQL-------------IEECR----PDCLISD
BvUGT73A4     94 -----IRDR-------FFKAAAMLRDQLEHF-------------LEKTR----PNCLVAD
Db5-GT        96 -----ANNE-------FFNAANLLKEQLENF-------------LVKTR----PNCLVAD
AtUGT86A1     97 -----FEGI-------LHVFSAHVDDLIAKLSR-----------RDDPP----VTCLIAD
ZmUFGT1      100 -----MEAA-------EAGGVKAWLEAARAA-------------AGGAR----VTCVVGD
CteUGT78K6    82 -----LDLF-------LSTGPDNLRKGIELAVA-----------ETKQS----VTCIIAD
AtUGT78D1     87 -----VELF-------LEAAPRIFRSEIAAAEI-----------EVGKK----VTCMLTD
CoUGT78B3     87 -----IDLF-------IKATPGNFIEAIHKAVE-----------ESDRK----ITCLTND
MtUGT78G1     92 -----IFLF-------IKAMQENFKHVIDEAVA-----------ETGKN----ITCLVTD
LgUGT78J1     79 -----LGLF-------LKACPHNLEKAIGEAEE-----------DTGLT----ICSLISD
CpPGT11       82 -----LEKR-------LQVMPGKLEGLIEEIHG-----------RGGEK----IACLIAD
AtUGT83A1     94 -----SESV-------LRFMPKKVEELIERMMAET---------SGGTI----ISCVVAD
FaUGT75T1     81 -----MSEL-------KRAGSESLTALIEKISTS----------DEHGP----ITFLIYT
AtUGT75C1     86 -----MSEL-------KRCGSNALRDIIKANLDAT---------TETEP----ITGVIYS
AtUGT75B1     82 -----SVNL-------KVNGDKALSDFIEATK------------NGDSP----VTCLIYT
AtUGT75D1     93 ATGNFMSEM-------RRRGKETLTELIEDNR------------KQNRP----FTCVVYT
ZmUGT74A1     76 -----LEKQ-------AAAASASLASLVEARA------------SSADA----FTCVVYD
AsUGT74H5     88 -----LAGM-------ESAGSRTLDELLRSEA------------EKGRP----IHAVVYD
BdUGT74J7     94 -----GRRL-------AAAGSETLEALFRSEA------------EAGRS----VRALVYD
SrUGT74G1     86 -----LETF-------KQVGSKSLADLIKKLQ------------SEGTT----IDAIIYD
AtUGT74B1     82 -----SESF-------KLNGSETLTLLIEKFK------------STDSP----IDCLIYD
RsUGT74R1     80 -----LQQF-------QRAVPGSLDDLIRLERG-----------HDQPQ----PTILIYD
LuUGT74S1     82 -----SQTF-------RRVGSETLTDLIRKQS------------ESRHP----VHCIIYD
AtUGT74F1     80 -----LQNF-------KTFGSKTVADIIRKHQ------------STDNP----ITCIVYD
SgUGT74AC1    80 -----LDRF-------RQKMTKNLEDFLQKAM------------VSSNP----PKFILYD
AtUGT74C1     79 -----LDRF-------HNSTSRSLTDFISSAK------------LSDNP----PKALIYD
AtUGT74D1     86 -----FAKF-------QENVSRSLSELISSM---------------DPK----PNAVVYD
AtUGT74E2     78 -----MERV-------ETSIKNTLPKLVEDMK------------LSGNP----PRAIVYD
AtUGT84B1     84 -----LKSL-------NKVGAMNLSKIIEEKR----------------------YSCIISS
DgpHBAGT      93 -----IPQF-------QTVGHDSFVQLVKKHS------------ESGTP----VSCIISN
MtUGT84F1     83 -----HKKL-------ELVGRQFISQMIKNHA------------DSNKP----ISCIINN
GtUF6CGT1     95 -----INHL-------DQTGRQKLPIMLKKHE------------ETGTP----VSCLILN
PgUGT84A23    91 -----LPQL-------EKVGKKVLIPQMIQKNA-----------EQGRP----VSCLINN
PgUGT84A24    90 -----LPQL-------EKVGKEVIPRMIKKNE------------EQNRP----VSCLINN
AtUGT76E1     80 -----LFKL-------NQICEASFKQCIGQLLQ-----------EQGND----IACVVYD
AtUGT76D1     79 -----VLEL-------NSVCEPLLKEFLT---------------NHDDV----VDFIIYD
SrUGT76G1     86 AGMR-IPII-------NEHGADELRRELELLMLAS---------EEDEE----VSCLITD
VvGT15       113 -----VLAA-------NVNCESPLRECLAEKQ------------EQHGD----IACIIHD
AtUGT76C1     78 -----LTLL-------NNNCQIPFRECLAKLIKPSSDSG-----TEDRK----ISCVIDD
AtUGT76B1     78 -----LHDL-------NSKCVAPFGDCLKKLI------------SEEPT----AACVIVD
CsUGT76F1     78 -----VSLL-------NTKCLVPFRDCLAKLLAD----------VEEEP----IACLISD
OsUGT709A4    88 -----VDSL-------LGAGQQAAYRALLGSLLVGSGGA-----GGFPP----VTSVVAD
SrUGT85C2     92 -----LRSI-------ETNFLDRFIDLVTKL---------------PDP----PTCIISD
CoUGT85N1     90 -----CKSM-------RNTCADPFRSLILKLNSS----------SDVPP----VTCIVAD
MeUGT85K4     91 -----SDST-------RKHCLAPFIDLIAKLKAS----------PDVPP----ITCIISD
AtUGT85A1     93 -----CEST-------MKNCLAPFRELLQRINAG----------DNVPP----VSCIVSD
MtUGT85H2     92 -----CQSV-------RKNFLKPYCELLTRLNHS----------TNVPP----VTCLVSD
MdUGT71K1     87 -----FSLY-------IESHVPSVKKIITNLVSSSANS------SDSIR----VAALVVD
AtUGT89A2     98 -----GNLP-------IMASLRQLREPIINWFQ-----------SHPNP----PIALISD
AtUGT89C1     90 -----AIVH-------MFDALSRLHDPLVDF-------------LSRQPPSDLPDAILGS
AtUGT89B1     95 -----GFPL-------MIHALGNLHAPLISWIT-----------SHPSP----PVAIVSD
NmUGT89P1     82 -----LAAR-------VHASSQ-LSNTIIQWFQ-----------SHTSP----PVAIVSD
AtUGT92A1     96 -----LVIS-------LLEASRSLREPFRDFMTKILK-------EEGQS----SVIVIGD
GbUGT92K1     98 -----LFLP-------LLQASQQLEQPFQQLVQDIVR-------KEGRL----PACIISD
GmUGT92G4     92 -----DLLK-------LGYASLTLEPPFRSLISQITE-------EDGHP----PLCIISD
PoUGT90A7     95 -----FFPK-------FATATKLMQPDFEQAL------------EKIPD----VTCIVSD
AtUGT90A1     94 -----LFVP-------FTRATKLLQPFFEETL------------KTLPK----VSFMVSD
AcUGT73J1     87 -----MFDL-------FVSALSLFQPPLQNL-------------IHDLK----PDCLISD
MtUGT73P1     98 -----IISK-------IYQGLAILQEQFTQL-------------FRDMK----PDFIVTD
SrUGT73E1     97 -----YWKN-------ISTAIDLLQQPAEDLLR-----------ELSPP----PDCIISD
CpPGT4        92 -----DFAR-------FMKSLHMLQQPFENLFK-----------EKTLK----PCCIISD
AtUGT73C1     94 -----ASLT-------FFKAFSLLEEPVEKLLK-----------EIQPR----PNCIIAD
CpPGT2        93 -----LAFD-------FFTAADMLQEPVENVFA-----------QLKPR----PNCIISD
PjGAT         99 -----LIGN-------FFSALNKLQQPLEQHLQ-----------ELMPP----PSCIISD
CtiUGT73AE1   95 -----LLQN-------FSLAVDMLQQRLEQRFE-----------SLEPR----PSCILSD
CaUGT73AH1    93 -----FSKN-------FIDASKMQQGPLEKIFG-----------DIKPS----PSCIISD
PzGAT2        93 -----MSIN-------FFAATKMLQEPVEKMLK-----------DIKPS----PSCILSD
AcUGT73G1     94 -----LKSL-------FFDAVAMLQQPFRAF-------------LKETN----PDCVVAG
MtUGT73K1     93 -----TAGK-------IHMAAHFVKADIEEF-------------MKENP----PDVFISD
GeUGT73F1     95 -----NLAK-------VFQATTLLRTPIEHF-------------VEENP----PDCIVAD
AtUGT73B1    101 NVGD-LSQK-------FLLAMKYFEEPLEEL-------------LVTMR----PDCLVGN
GuUGAT        91 -----KFIK-------FMKATLLLRDPLEHV-------------LQEEQ----PHCLVAD
NmUGT73BD1    90 -----MFMK-------FFRGLSLLQEPFEQI-------------LQELS----PDCLVAD
NmUGT73BD1     1 ---------------------------------------MTAQFHVVFFP-LM-AQGHLIP
```

```
AtUGT81A1     181 L----WTD-HTPWPFNQLPRSYNFLVKHGTLWKMTYYGTSPRIVHQSNFAATSTFI----
AtUGT80A2     169 RGESKNGV-CADESIDGVDL-QYIPPMQIVMLIVGTRGDVQPFVAIAKRLQDYGHRVRLA
AtUGT80B1     144 KSLT-----ESFRSIPRLKIAILVVGTRGDVQPFLA--MAKRLQEFGHRVRLATHANFRS
OsZOGT3       132 TINS-FAV-EEAARL---PNGEAFPVSCVAVSALAL--HIDTGHR--------------
OsZOGT1       127 RLNS-FAA-AQAARL---PNGEAF---GLQCVAMSY--NIGWLDPEHR-----------
ZmcisZOG1     128 RLNP-FAA-TEAARL------ANADAFGLQCVAISY--NVGWLDPGHRLLSDYG------
GmUGT79A6     116 FAQH-WLP-KLASEV-GIKS--VHFSVYSAISDAYI--TVPSRFADVEGRNITFEDLKKP
AtUGT79B6     115 FAH--WIP-EIAREY-GVKS-VNFITISAACVAISF---VPGRSQDDLGST---------
GmUGT91H9     117 FATE-WLP-PIAKSL-NIPC-AHYNLTAAWNKVFID--PPKDYQLNNSITLQDMCLP---
ZmUGT91L1     127 NFHA-SMA-DVASEH-KVPC-ILNMPYSAATTE--------------------------
PoUGT95A1     141 VMMS-WSK-EIFVDH-EIPV-VSFSTSGATASAVGY--GMWKAEVGNMKPGEIRE-----
MtUGT95B4     100 VMMS-WSN-NVFKKF-EIPT-VAFFTSGACSAAMEL--AAWKAQPLDLKYGEICF-----
PgUGT95B2     118 VMMS-WTA-EIFQKF-RIPL-VGFFTSGACSAAMEF--AQWKAGVDGLK-PGETRLLPGL
VvUGT95B6     117 VMMS-WSK-DIFHKF-NIPV-VSFFTSGACSAAMEY--ASWKAGAFNIKPGEVLP-----
AtUGT82A1     110 LLAS-WAI-GVADRC-GVPV-AGFWPVMFAAYRLIQ--AIPELVRTGLVSQKGCPRQLEK
VpUGT94F1     123 VFNP-WAA-KHALSH-GSPS-VWFMASGATICSFHYHQHLHKTGSLVPYEGVDFGEIK--
AtUGT87A1     101 TYII-WAV-RVGTKR-NIPV-ASFWTTSATILSLFINSDLLASHGHFPIEPSESKLDEI-
AtUGT72C1     113 LLGT-EAL-EVAKEL-GIMRKHVLVTTSAWFLAFTV--YMASLDKQELYKQLSSIG----
AtUGT72D1     115 FLGT-ELM-SVADDV-GMTAKYVYVPTHAWFLAVMV--YLPVLDTVVEGEYVDIKE----
LjUGT72AD1    122 GLIT-QVL-PMANEL-NVLS-YAYFPSTAMLLSLCL--YPSTFDKTFPCE----------
AtUGT72B1     118 LFGT-DAF-DVAVEF-HVPP-YIFYPTTANVLELTE-----------------------
VvUGT1        116 LFGT-DAF-DVANEF-GIPP-YIFFPTTAMVLSLIF--HVPELDQKFSCEYRDLPE----
LjUGT72AH1    116 AFAH-EAM-EFGKEL-NMLS-YIYFPCSIMMLSLGL--HSSKLDET----VSCEYRDHPQ
LjUGT72Z2     117 PFAN-EAL-EIGKEF-NLLS-YIYFPPSAMTLSLFL--HLPKLHEEVSCEYRDLTE----
GmUGT72X4     116 VFAF-QAL-EYAKEF-NALS-YFYFPSSAMILSLLM--HAPKLDEEVSGEYKDLTE----
MtUGT72L1     123 AFAV-EAL-NFAKEF-NMLS-YIYFCAAASTLAWSF--YLPKLDEETTCEYRDLPE----
PgUGT72BD1    118 LFGT-EAF-AIADEF-HMLK-YEFFTTNAWFLALTL--YAPHVDREHEDDHILRHM----
AtUGT72E1     108 LFGL-DAI-PLGGEF-NMLT-YIFIASNARFLAVAL--FFPTLDKDMEEEHIIKKQ----
OsUGT706C1    115 MFCI-DAL-DVAAEL-AVPA-YMFYPSAASDLAIYL--QVPHVARSAPSSFKDMADT---
OsUGT706D1    115 IFCA-EAV-DAAAAL-HVPA-YIYFTSAAGAFAASL--GLMHHYSTTTNLRDMGKA---
MtUGT88E1     119 FLNY-SAS-QVTNNL-EIPT-YFYYTSGASLLCLFL--NFPTFHKNATIPIKDYNMHT--
VpUGT88D8     107 FFCN-SAF-EVSTSL-NIPT-YFYVSSGGFGLCAFL--HFPTTDEIIPQDIGDLND----
NmUGT88P1     118 FFCT-AAV-EVSSNL-EIPT-YFFFTSGSSAMCQFL--YLPTLHETITQKDLQDPNT---
AtUGT88A1     120 FFCT-AVL-DITADF-TFPV-YFFYTSGAACLAFSF--YLPTIDETTPGKNLKDIP----
VvGT7         117 CFCT-AAL-SVAAQL-NIPF-YYFFTSGACCLASFL--YLPFIHQQTTKSFKDLNT----
MdUGT88F1     119 LFCT-SAL-PIGKEF-NIPT-YYFCTSGAAILAAFL--YLPKIDEQTKTTESFKDLRDT-
ScUGT5        116 FFCT-SAF-PISESL-GIPV-YYFFTSGLAAVAAYL--YFPTIDKQVDQSFKDLVDT---
OsUGT707A2    135 WFCT-TLL-DVTRDL-GLPG-YVFFTSAASMLSLLL--RLPALDKEVAVDFEEMGG----
AtUGT71B1     111 MFCT-SMI-DIADEF-NLSA-YIFYTSNASYLGLQF--HVQSLYDEKELDVSEFKDTEM-
MdUGT71A15    124 MFSA-SLI-DVANEF-KVPS-YLFFTSNASALALMS--HFQSLRDEGGIDITELTSSTA-
BvUGT71F1     130 MFCT-NMI-DIATNL-NVPA-YIFFTSGANMLSLMF--HFQSMNDEGVFDLTKDHISPNA
Db6-GT        120 MLCP-AMM-DVAEEL-EVPS-YILFTSGANLLNVVF--HFLSLADNGV--DIATEVNDPD
AtUGT71C1     132 FFCV-PMI-DVGNEF-NLPS-YIFLTCSAGFLGMMK--YLPERHREIKSEFNRSFNEE--
AtUGT71C4     128 LFCN-SLVKDVGNEL-NLPS-YIYLTCNARYLGMMK--YIPDRHRKIASEFDLSSGDE--
AtUGT71D1     123 FFCL-PMI-DVAKDI-SLPF-YVFLTTNSGFLAMMQ--YLADRHSRDTSVFVRNSEE---
FaUGT71W2     115 MFCT-TMI-DIANEL-KVPS-FLFFTSGAAFLGLLL--CLPERYDLVGKEFVHSDP----
MtUGT71G1     122 FFCV-SMI-DVGNEF-GIPS-YLFLTSNVGFLSLML--SLKNRQIEEVFDDSDRDHQ---
MdUGT71K1     125 LFCV-SMI-DVAKEL-NIPS-YLFLTSNAGYLAFML--HLPILHE-----KNQIAVEESD
AtUGT89A2     131 FFLG-WTH-DLCNQI-GIPR-FAFFSISFFLVSVLQ--FCFENIDLIKSTDPIH------
AtUGT89C1     125 SFLSPWIN-KVADAF-SIKS-ISFLPINAHSISVMW-----------------------
AtUGT89B1     128 FFLG-WTK-----NL-GIPR-FDFSPSAAITCCILN--TLWIEMPTKINEDDDNEILH--
NmUGT89P1     114 FFLG-WTN-SLASQL-GIPR-LVFWPSGVQRSSLVD--YIWQNDQLSDSDHQIQDNSVIS
AtUGT92A1     133 FFLG-WIG-KVCKEV-GVYS-VIFSASGAFGLGCYR--SIWLNLPHKETKQDQFL-----
GbUGT92K1     135 IFLG-WTL-DVANRL-GIPR-IMFCTCGAYSTSIYY--SLWAHLPHRQTDSDTFC-----
GmUGT92G4     129 MFLG-WVN-NVAKSL-GTRN-LTFTTCGAYGILAYI--SIWSNLPHRKTDSDEFH-----
PoUGT90A7     127 GFLS-WTL-ASANKF-RIPR-LAFYGMNNYVGAVSR--DVALNRLLSGPESDDELLT---
AtUGT90A1     126 GFLW-WTS-ESAAKF-NIPR-FVSYGMNSYSAAVSI--SVFKHELF----TEPESKSDTE
AcUGT73J1     118 SLFP-WTA-DLALQF-KIPR-IIFHGAGVFPMYVSA--NIFSHFPLDESKEEFF------
MtUGT73P1     129 MFYP-WSV-DVADEL-GIPR-LICIGGSYFAHSAMN--SIEQFEPHAKVKSNSVSFL---
SrUGT73E1     130 FLFP-WTT-DVARRL-NIPR-LVFNGPGCFYLLCIH--VAITSNILGENEPVSSNTE---
CpPGT4        125 MCFP-WTV-DTAAKF-NVPR-IIFHGFSCFCLFCHH--LLGVSKVHENVTSDSE------
AtUGT73C1     127 MCLP-YTN-RIAKNL-GIPK-IIFHGMCCFNLLCTH--IMHQNHEFLETIESDKEYFP--
CpPGT2        126 MCLP-YTA-HIAGKF-NIPR-ITLHGTCCFCLVCYN--NLFTSKVFESVSSESEYLV---
PjGAT         132 KYLS-WTT-KTAEKI-HVPR-LVFHGMCCFSLLSSH--NIRLYNAHLSVTSDSQPFV---
CtiUGT73AE1   128 RYML-WTA-DTAVKH-GLPR-IIFDGMNCFKQLCTH--NMYLSNVLDG-------LSDSD
CaUGT73AH1    126 KHIM-WTA-KTAKKF-QIPW-IVFDGMSCFNQLCTE--ILHTTKVHENVSESEPFV----
PzGAT2        126 KHVF-WTS-DTAKKL-QIPW-IMFDGMSCFTQLCTE--NIYNSKVHESVSSESESFV---
AcUGT73G1     125 LFLA-WIH-NVASEL-NIPS-LDFHGSNFSSKCMSH--TVEHHNLLDNSTAETVL-----
MtUGT73K1     124 IIFT-WSE-STAKNL-QIPR-LVFNPISIFDVCMIQ--AIQSHPESFVSDSGPYQ-----
GeUGT73F1     126 FIYQ-WVD-ELANKL-NIPR-LAFNGFSLFAICAIESVKAHSLYASG-----------
AtUGT73B1     136 MFFP-WST-KVAEKF-GVPR-LVFHGTGYFSLCASHCIRLPKNVATSSEPFV--------
GuUGAT        122 MFFP-WAT-DSAAKF-GIPR-IVFHGLGYFPLCVLA--CTRQYKPQDKVSSYTEPFV---
NmUGT73BD1    121 MFFP-WTT-NVAAKY-NIPR-FVFHGFCLFALCVSE--NIRSYKPFKDVESDSEPIV---
NmUGT73BD1      1 -------------------------------------MTAQFHVVFFP-LM-AQGHLIP
```

```
NtTOGT1      120 MFLP-WTT-DTAAKF-NIPR-IVFHGTSFFALCVEN--SVRLNKPFKNVSSDSETFV---
BvUGT73A4    125 MFFP-WAT-DSAAKF-NIPR-LVFHGHCLFALCALE--IIRLHEPYNNASSDEEPFL---
Db5-GT       127 MFFT-WAA-DSTAKF-NIPT-LVFHGFSFFAQCAKE--VMWRYKPYKAVSSDTEVFS---
AtUGT86A1    130 TFYV-WSS-MICDKH-NLVN-VSFWTEPALVLNLYY--HMDLLISNGHFKSLDNRKDV--
ZmUFGT1      131 AFV--WPAADAASA-GAPW-VPVWTAASCALLAHI--RTDALREDVGDQAANRVDEP--
CteUGT78K6   115 AFVT-SSL-LVAQTL-NVPW-IAFWPNVSCSLSLYF--NIDLIRDKCSKDAKNAT-----
AtUGT78D1    120 AFF--WFAADIAAEL-NATW-VAFWAGGANSLCAHL--YTDLIRETIGLKDVSMEET---
CoUGT78B3    120 SFM--WMGVDIAQTL-QVPC-VSVWAPGASSLCAHL--YTDILRQNIGVGANAKYDEY--
MtUGT78G1    125 AFF--WFGADLAEEM-HAKW-VPLWTAGPHSLLTHV--YTDLIREKTGSKEVHDVKS---
LgUGT78J1    112 AFL--WFSCDLAEKR-GVPW-VALWTSASCSLSAHM--YTHEILQALES-GVAERDEHDK
CpPGT11      115 GAAG-WAI-EVAEKM-KLRR-AVVVITSAATVALTF--SIPKLIEDGVINSNGTPIKEQM
AtUGT83A1    129 QSLG-WAI-EVAAKF-GIRR-TAFCPAAAASMVLGF--SIQKLIDDGLIDSDGTVRVNKT
FaUGT75T1    115 VLLP-WAA-EVASSF-GIASAFLCITSATSFAICGH--YFKDYYKSQSSLPFPS------
AtUGT75C1    121 VLVP-WVS-TVAREF-HLPT-TLLWIEPATVLDIYY--YYFNTSYKHLFDVE--------
AtUGT75B1    114 ILLN-WAP-KVARRF-QLPS-ALLWIQPALVFNIYY--THFMGNKS--------------
AtUGT75D1    130 ILLT-WVA-ELAREF-HLPS-ALLWVQPVTVFSIFY--HYFNGYEDAISEMANTPSS---
ZmUGT74A1    108 SYED-WVL-PVARRM-GLPA-VPFSTQSCAVSAVYY--HFSQGRLAVPPGAAADGSDGGA
AsUGT74H5    120 AFLQPWVP-RVARLH-GAAC-VSFFTQAAAVNVAYS-------RRVGKI-----------
BdUGT74J7    126 PHLP-WAA-RVARAA-GVRT-AAFFSQPCAVDLIYG--EVWSGRVGLPIKDGSA------
SrUGT74G1    118 SMTE-WVL-DVAIEF-GIDG-GSFFTQACVVNSLYY--HVHKGLISLPLGE---------
AtUGT74B1    114 SFLP-WGL-EVARSM-ELSA-ASFFTNNLTVCSVLR--KFSNGDFPLPADPNSAPFR---
RsUGT74R1    113 SFFP-WAL-DVAHSN-GLAA-APFFTQTCSVSSVYF--LFKEGRLSDEMEL---------
LuUGT74S1    114 ASMP-WFL-DVAKRF-GIVG-AAFLTQSCAVNAIYY--HLREGTIKRPVVSDPAAG----
AtUGT74F1    112 SFMP-WAL-DLAMDF-GLAA-APFFTQSCAVNYINYLSYINNGSLTLP------------
SgUGT74AC1   112 STMP-WVL-EVAKEF-GLDR-APFYTQSCALNSINY--HVLHGQLKLPPETP--------
AtUGT74C1    111 PFMP-FAL-DIAKDL-DLYV-VAYFTQPWLASLVYY--HINEGTYDVPVDRHENPT----
AtUGT74D1    115 SCLP-YVL-DVCRKHPGVAA-ASFFTQSSTVNATYI--HFLRGEFKEFQN----------
AtUGT74E2    110 STMP-WLL-DVAHSY-GLSG-AVFFTQPWLVTAIYY--HVFKGSFSVPSTKYGHST----
AtUGT84B1    111 PFTP-WVP-AVAASH-NISC-AILWIQACGAYSVYYRYYMKTNSFPDLEDLNQTVE----
DgpHBAGT     125 PFIP-WVS-DVAAEL-GIPC-ALLWVQSCFVYSVYY--HYFHNLASFPTVDQPNK-----
MtUGT84F1    115 PFFP-WVS-DIAFEH-NIPS-ALLWTNSSAVFTICY----DYVHKLLPFPSNEEPYI---
GtUF6CGT1    127 PLVP-WVA-DVADSL-QIPC-ATLWVQSCASFSAYY--HYHHGLVPFPTESEPEI-----
PgUGT84A23   123 PFIP-WVS-DVAETL-GLPS-AMLWVQSCACFLAYY--HYYHGLVPFPSENAMEI-----
PgUGT84A24   122 PFIP-WVS-DVAESL-GLPS-AMLWVQSCACFAAYY--HYYHGLVPFPSESAMEI-----
AtUGT76E1    113 EYMY-FSQ-AAVKEF-QLPS-VLFSTTSATAFVCRS--VLSRVNAESFLLDMKDPKVS--
AtUGT76D1    108 EFVY-FPR-RVAEDM-NLPK-MVFSPSSAATSISRC--VLMENQSNGLLPPQDARSQL--
SrUGT76G1    125 ALWY-FAQ-SVADSL-NLRR-LVLMTSSLFNFHAHV--SLPQFDELGYLDPDDKTRLEEQ
VvGT15       145 ITMY-FAE-AVANHL-KVPS-INLVTSNVSTTIAHN--AFPSLLEKGHIPLQGSTL----
AtUGT76C1    117 SGWV-FTQ-SVAESF-NLPR-FVLCAYKFSFFLGHF--LVPQIRREGFLPVPDSEA----
AtUGT76B1    110 ALWY-FTH-DLTEKF-NFPR-IVLRTVNLSAFVAFS--KFHVLREKGYLSLQETKADSP-
CsUGT76F1    112 AMLP-FTQ-AVADSL-KLPR-IVLRTGGASSFVVFA--AFPLLKERGYFPIQDSKGQEP-
OsUGT709A4   126 ALLT-FAI-DVAEEL-GVPA-LAFRTASASSLLAYM--SVPRLFELGELPFPPGGDLDEP
SrUGT85C2    121 GFLSVFTI-DAAKKL-GIPV-MMYWTLAACGFMGFY--HIHSLIEKGFAPLKDASYLTNG
CoUGT85N1    124 VAMD-FTL-QVSEEL-GPPV-VLFFTLSGCGVLGYM--HYGELLERGYFPLREESFLSNG
MeUGT85K4    125 GVMA-FAI-DAARHF-GILE-IQFWTTSACGFMAYL--HHIELVRRGIVPFKDESFLHDG
AtUGT85A1    127 GCMS-FTL-DVAEEL-GVPE-VLFWTTSGCAFLAYL--HFYLFIEKGLCPLKDESYLTKE
MtUGT85H2    126 CCMS-FTI-QAAEEF-ELPN-VLYFSSSACSLLNVM--HFRSFVERGIIPFKDESYLTNG
MdUGT71K1    125 LFCV-SMI-DVAKEL-NIPS-YLFLTSNAGYLAFML--HLPILHE-----KNQIAVEESD
AtUGT89A2    131 FFLG-WTH-DLCNQI-GIPR-FAFFSISFFLVSVLQ--FCFENIDLIKSTDPIH------
AtUGT89C1    125 SFLSPWIN-KVADAF-SIKS-ISFLPINAHSISVMW------------------------
AtUGT89B1    128 FFLG-WTK-----NL-GIPR-FDFSPSAAITCCILN--TLWIEMPTKINEDDDNEILH--
NmUGT89P1    114 FFLG-WTN-SLASQL-GIPR-LVFWPSGVQRSSLVD--YIWQNDQLSDSDHQIQDNSVIS
AtUGT92A1    133 FFLG-WIG-KVCKEV-GVYS-VIFSASGAFGLGCYR--SIWLNLPHKETKQDQFL-----
GbUGT92K1    135 IFLG-WTL-DVANRL-GIPR-IMFCTCGAYSTSIYY--SLWAHLPHRQTDSDTFC-----
GmUGT92G4    129 MFLG-WVN-NVAKSL-GTRN-LTFTTCGAYGILAYI--SIWSNLPHRKTDSDEFH-----
PoUGT90A7    127 GFLS-WTL-ASANKF-RIPR-LAFYGMNNYVGAVSR--DVALNRLLSGPESDDELLT---
AtUGT90A1    126 GFLW-WTS-ESAAKF-NIPR-FVSYGMNSYSAAVSI--SVFKHELF----TEPESKSDTE
AcUGT73J1    118 SLFP-WTA-DLALQF-KIPR-IIFHGAGVFPMYVSA--NIFSHFPLDESKEEFF------
MtUGT73P1    129 MFYP-WSV-DVADEL-GIPR-LICIGGSYFAHSAMN--SIEQFEPHAKVKSNSVSFL---
SrUGT73E1    130 FLFP-WTT-DVARRL-NIPR-LVFNGPGCFYLLCIH--VAITSNILGENEPVSSNTE---
CpPGT4       125 MCFP-WTV-DTAAKF-NVPR-IIFHGFSCFCLFCHH--LLGVSKVHENVTSDSE------
AtUGT73C1    127 MCLP-YTN-RIAKNL-GIPK-IIFHGMCCFNLLCTH--IMHQNHEFLETIESDKEYFP--
CpPGT2       126 MCLP-YTA-HIAGKF-NIPR-ITLHGTCCFCLVCYN--NLFTSKVFESVSSESEYLV---
PjGAT        132 KYLS-WTT-KTAEKI-HVPR-LVFHGMCCFSLLSSH--NIRLYNAHLSVTSDSQPFV---
CtiUGT73AE1  128 RYML-WTA-DTAVKH-GLPR-IIFDGMNCFKQLCTH--NMYLSNVLDG-------LSDSD
CaUGT73AH1   126 KHIM-WTA-KTAKKF-QIPW-IVFDGMSCFNQLCTE--ILHTTKVHENVSESEPFV----
PzGAT2       126 KHVF-WTS-DTAKKL-QIPW-IMFDGMSCFTQLCTE--NIYNSKVHESVSSESESFV---
AcUGT73G1    125 LFLA-WIH-NVASEL-NIPS-LDFHGSNFSSKCMSH--TVEHHNLLDNSTAETVL-----
MtUGT73K1    124 IIFT-WSE-STAKNL-QIPR-LVFNPISIFDVCMIQ--AIQSHPESFVSDSGPYQ-----
GeUGT73F1    126 FIYQ-WVD-ELANKL-NIPR-LAFNGFSLFAICAIESVKAHSLYASG-------------
AtUGT73B1    136 MFFP-WST-KVAEKF-GVPR-LVFHGTGYFSLCASHCIRLPKNVATSSEPFV--------
GuUGAT       122 MFFP-WAT-DSAAKF-GIPR-IVFHGLGYFPLCVLA--CTRQYKPQDKVSSYTEPFV---
NmUGT73BD1   121 MFFP-WTT-NVAAKY-NIPR-FVFHGFCLFALCVSE--NIRSYKPFKDVESDSEPIV---
NmUGT73BD1     1 ------------------------------------MTAQFHVVFFP-LM-AQGHLIP
```

```
AtUGT81A1     232 ----------AREIAQG-------LMKYQPDIII--------------------------
AtUGT80A2     227 THANFKEFVLTAGLEFY--PLGG-DPKVLAGYMV-------------------KNKGFLP
AtUGT80B1     197 FVR-------AAGVEFY--PLGG-DPRELAAYMA-------------------RNKGLIP
OsZOGT3       170 ----------LLRENGL-------NHAPLETYMT-------------------QE-----
OsZOGT1       165 ----------LVREHGL-------QFHPVEACMT--------------------------
ZmcisZOG1     172 ----------LQFLP--------------PDACM--------------------------
GmUGT79A6     169 ----------PPGYPQN-SNISLKAFEAMDFMFL-------------------FTRFG--
AtUGT79B6     158 ----------PPGYPSSKVLL---RGHETNSLSF--------------------LSYPFG
GmUGT91H9     168 ----------PTWLPFT-------TTVHLRPHEI-------------------RRATSSI
ZmUGT91L1     156 ----------DFGIP---------DPSVLPMFRP--------------------------
PoUGT95A1     190 ----------IPGLPKE-MAVTFADLSRGPQRRIRPPGGPGKSDGRAGPPNRMRSGSRHG
MtUGT95B4     149 ----------LPGLPYD-MAL---TYSDLKQHLH--------DPPPPPPPQHGIPPPPHE
PgUGT95B2     171 PEEMALTEFDLKKRPHG--PPHLRNGGGGPGGGG--------APGGGGFPFAPPPGRPGP
VvUGT95B6     166 ----------LPGLPED-MAL---TYSDLQRGPHGPPGGPPGPPGSGGPPGPPGARGPPR
AtUGT82A1     164 ----------TIVQPEQ--PL-L-SAEDLPWLIG-------------------TPKAQ-K
VpUGT94F1     177 ----------RHISPNT-------KGADFGGFIL--------------------------
AtUGT87A1     156 ----------VDYIPGL-SPT---RLSDLQILHG-------------------YSHQ---
AtUGT72C1     164 ----------ALLIPGC-SPVKFERAQDPRKYIR--------------------------
AtUGT72D1     166 ----------PLKIPGC-KPV---GPKELMETML-------------------DRSGQQ-
LjUGT72AD1    166 ----------TIEIPGC-IPI---HITDLPNQIQ-------------------NRYNE--
AtUGT72B1     150 ----------PLMLPGC-VPV---AGKDFLDPAQ-------------------DRKDD--
VvUGT1        166 ----------PVKFPGC-VPV---QGRDLIDPLQ-------------------DRKNE--
LjUGT72AH1    166 P----------IEIPGC-ISV---HGKDLPNSIQ-------------------NRSSP--
LjUGT72Z2     167 ----------PIQIPGC-IPI---QGQDLPEHFQ-------------------DRSSL--
GmUGT72X4     166 ----------PIRLPGC-VPV---MGVDLPDPAQ-------------------DRSSE--
MtUGT72L1     173 ----------PIKVPGC-VPL---HGRDLLTIVQ-------------------DRSSQ--
PgUGT72BD1    168 ----------PLHIPGC-ASI---LYEDTVDVYT-------------------DRNDKLL
AtUGT72E1     158 ----------PMVMPGC-EPV---RFEDTLETFL-------------------DPNSQL-
OsUGT706C1    166 ----------VLSFSGVPTI----RALDMPDTMQ-------------------DRESD--
OsUGT706D1    166 ----------LLRFPGV-PPI---PASDMPSLVQ-------------------DREGR--
MtUGT88E1     171 ----------PIELPGL-PRL---SKEDYPDEGK-------------------DPSSP--
VpUGT88D8     157 ----------YLEIPGC-PPV---HSLDFPKGMF-------------------FRHTN--
NmUGT88P1     169 ----------YVHIPGI-PPI---HSLDLPKVLS-------------------NRSTV--
AtUGT88A1     170 ----------TVHIPGV-PPM---KGSDMPKAVL-------------------ERDDE--
VvGT7         167 ----------HLHIPGL-PPV---PASDMAKPIL-------------------DREDK--
MdUGT88F1     172 ----------VFEFPGWKSPL---KATHMVQLVL-------------------DRNDPA-
ScUGT5        167 ----------KFHIPGL-PPL---PSRHMPQPVL-------------------NRNDP--
OsUGT707A2    185 ----------AVDLPGL-PPV---PAALLPTPVM--------------------KKGC--
AtUGT71B1     164 ----------KFDVPTL-TQP-F-PAKCLPSVML-------------------NKK----
MdUGT71A15    177 ----------ELAVPSFINPY---PAAVLPGSLL--------------------------
BvUGT71F1     184 ----------EFDVPGFVNRV---PEKVLPAVLI-------------------DKES---
Db6-GT        172 K---------EVDVPGF-RNR-V-PCKVLPLPFL--------------------------
AtUGT71C1     184 ----------LNLIPGYVNSV---PTKVLPSGLF-------------------MKE----
AtUGT71C4     181 ----------ELPVPGFINAI---PTKFMPPGLF--------------------------
AtUGT71D1     174 ----------MLSIPGFVNPV---PANVLPSALF-------------------VEDG---
FaUGT71W2     165 ----------DSIVPSYVNPV---PTNVLPGFVF-------------------NNGG---
MtUGT71G1     173 ----------LLNIPGISNQV---PSNVLPDACF-------------------NKDGG--
MdUGT71K1     174 P---------DWSIPGIVHPV---PPRVLPAALT---------------------DGR--
AtUGT89A2     179 ----------LLDLPRA-PIF---KEEHLPSIVR------------------RSLQTPSP
AtUGT89C1     158 ------------------------AQEDRSFFND--------------------------
AtUGT89B1     176 ----------FPKIPNC-PKY---RFDQISSLYR-----------------SYVHGDP--
NmUGT89P1     168 FPD-------VPNSPAY--PK-W-QACGLSTQY--------------------KKGDP--
AtUGT92A1     182 ----------LDDFPEA-GEI---EKTQLNSFML-------------------EADGTDD
GbUGT92K1     184 ----------VPDLPHI--RL---HRSQLSQILA-----------------VSEASDP--
GmUGT92G4     178 ----------VPGFPQN-YRF---HKTQLHRFLQ-------------------AADGTDD
PoUGT90A7     178 ----------VPTFPWIKITR---NDFDFPLNQR-------------------DPSGP--
AtUGT90A1     176 PVT-------VPDFPWI--KV---KKCDFDHGTT-------------------EPEES-G
AcUGT73J1     166 ----------MDGLAEK-IKL---YRKGLPDMFS--------------------------
MtUGT73P1     180 ----------LPGLPHN-VEM---TRLQLPDWLR-------------------APN----
SrUGT73E1     181 ----------RVVLPGL--PDRI-EVTKLQIVGS-------------------SRPANVD
CpPGT4        173 ----------YFNIPGL--PDHI-QFTKVQLLIS-------------------KRDDD--
AtUGT73C1     179 ----------IPNFPDR-VEF---TKSQLPMVLV---------------------AGD--
CpPGT2        177 ----------VPCLPDK-IEF---TTQQVDSSLG--------------------------
PjGAT         183 ----------VPGMPQR-VEI---TKAQLPGAFV---------------------TLP--
CtiUGT73AE1   175 PFI-------LPGLPDR-IEI---TKVQLPQEFN-------------------YSDLG--
CaUGT73AH1    176 ----------VPGLPDV-IEF---KRAQLPGLFN-------------------PGSNPGS
PzGAT2        177 ----------VRGLPDH-IEF---TKAQLPGLFN-------------------PGSVP--
AcUGT73G1     174 ----------LPNLPHK-IEM---RRALIPDFRK-------------------VAPS---
MtUGT73K1     173 ----------IHGLPHP-LTL---PIKPSPGFAR--------------------------
GeUGT73F1     169 ----------SFVIPGL-PHP---IAMNAAPPKQ--------------------------
AtUGT73B1     184 ----------IPDLPGD-ILITEEQVMETEEESV--------------------------
GuUGAT        173 ----------VPNLPGE-ITLTKMQLPQLPQHDK--------------------------
NmUGT73BD1    172 ----------LPNFPNQ-IEF---SKAQVPGSDL-------------------GIKD---
NmUGT73BD1      1 --------------------------------------MTAQFHVVFFP-LM-AQGHLIP
```

```
NtTOGT1       171 ----------VPDLPHE-IKL---TRTQVSPFER--------------------SGEET--
BvUGT73A4     176 ----------LPHLPHE-IEL---TRLQFSEELW--------------------KNGG---
Db5-GT        178 ----------LPFLPHE-VKM---TRLQVPESMR--------------------KGEET--
AtUGT86A1     182 ----------IDYVPGV-KAI---EPKDLMSYLQ--------------------VSDKDVD
ZmUFGT1       183 ----------LISHPGL-ASY---RVRDLPDGVV--------------------SGDFN--
CteUGT78K6    164 ----------LDFLPGL-SKL---RVEDVPQDML--------------------DVGEKET
AtUGT78D1     171 ----------LGFIPGM-ENY---RVKDIPEEVV------------------FEDLDSV--
CoUGT78B3     172 ----------LTFIPAM-EKV---RAGDLPDEIL---------------------KGNLDS
MtUGT78G1     176 ----------IDVLPGF-PEL---KASDLPEGVI-------------------KDIDVP--
LgUGT78J1     165 I---------QPLIPGL-EMA---TFRDLPPEVF--------------------LDKNPSP
CpPGT11       169 IQL-------APNMPAI-------STGELFWTRF-----------------------GDLT
AtUGT83A1     183 ----------IQLSPGM-PKM---ETDKFVWVCL--------------------KNKESQK
FaUGT75T1     164 ----------CITIDGL-PPF---ASDELPSYLL---------------------PTSPHV
AtUGT75C1     167 ----------PIKLPKL-PLI---TTGDLPSFLQ---------------------PSKA--
AtUGT75B1     154 ----------VFELPNL-SSL---EIRDLPSFLT--------------------PSNTNKG
AtUGT75D1     181 ----------SIKLPSL-PLL---TVRDIPSFIV---------------------SSNVYA
ZmUGT74A1     162 GAAALSEA--FLGLPEM-------ERSELPSFVF---------------------DHGP--
AsUGT74H5     159 ----------EEGLPAG-----F-EAEDLPTFLT---------------------LPLP--
BdUGT74J7     174 ----------LRGLLSL--EL---EPEDVPSFVA--------------------APDSYRL
SrUGT74G1     163 ----------TVSVPGF-PVL---QRWETPLILQ--------------------NHEQIQS
AtUGT74B1     165 ----------IRGLPSL-------SYDELPSFVG--------------------RHWLTHP
RsUGT74R1     158 ----------PHGIPRL-------EQRDLPSFIQ--------------------DKENSAH
LuUGT74S1     164 ----------TLVIDGL-PPL---EVSDLPSFIW----------------------DDL--
AtUGT74F1     156 ----------IKDLPLL-------ELQDLPTFVT---------------------PTGSHL
SgUGT74AC1    158 ----------TISLPSM-PLL---RPSDLPAYDF--------------------DPASTDT
AtUGT74C1     161 ----------LASFPGF-PLL---SQDDLPSFAC---------------------EKGSYP
AtUGT74D1     160 ----------DVVLPAM-PPL---KGNDLPVFLY--------------------DNNLCRP
AtUGT74E2     160 ----------LASFPSF-PML---TANDLPSFLC---------------------ESSSYP
AtUGT84B1     163 ----------LPALPLL-------EVRDLPSFML--------------------PSGGAH-
DgpHBAGT      174 ----------SVELPGL-PTL---ESDEIPSFLH--------------------PVYSPDK
MtUGT84F1     164 ----------DVQLNSS-IVL---KYNEIPDFIH---------------------------
GtUF6CGT1     176 ----------DVQLPGM-PLL---KYDEVPDYLH---------------------PRTPYP
PgUGT84A23    172 ----------DVQLPSM-PLL---KHDEVPSFLY---------------------PTTPYP
PgUGT84A24    171 ----------DVQLPCM-PLL---KHDEVPSFLY---------------------PTTPYP
AtUGT76E1     165 ----------DKEFPGL-HPL---RYKDLPTSAF-----------------------GP--
AtUGT76D1     160 ----------EETVPEF-HPF---RFKDLPFTAY---------------------------
SrUGT76G1     179 ----------ASGFPML-------KVKDIKSAYS---------------------------
VvGT15        195 ----------HDPVPEL-HPL---RFKDLPISRL--------------------GDLEA--
AtUGT76C1     167 ----------DDLVPEF-PPL---RKKDLSRIMG------------------TSAQSKP--
AtUGT76B1     163 ----------VPELPYL-------RMKDLPWFQT--------------------EDPRS--
CsUGT76F1     165 ----------VVELPPL-------KIKDLPVINT--------------------RDPET--
OsUGT709A4    180 ----------VRGVPGMEGFL---RRRDLPSTFR---------------------RHGNDH
SrUGT85C2     176 YLDTV-----IDWVPGM-EGI---RLKDFPLDWS-------------------TDLNDK--
CoUGT85N1     178 YLDTE-----IDWIPAM-KGI---RLKDLPSFLR---------------------TTDPDD
MeUGT85K4     179 TLDQP-----VDFIPGM-PNM---KLRDMPSFIR------------------VTDVNDI--
AtUGT85A1     181 YLEDTV----IDFIPTM-KNV---KLKDIPSFIR------------------TTNPDDV--
MtUGT85H2     180 CLETK-----VDWIPGL-KNF---RLKDIVDFIR------------------TTNPNDI--
MdUGT71K1     174 P---------DWSIPGIVHPV---PPRVLPAALT----------------------DGR--
AtUGT89A2     179 ----------LLDLPRA-PIF---KEEHLPSIVR-------------------RSLQTPSP
AtUGT89C1     158 ------------------------AQEDRSFFND---------------------------
AtUGT89B1     176 ----------FPKIPNC-PKY---RFDQISSLYR------------------SYVHGDP--
NmUGT89P1     168 FPD-------VPNSPAY--PK-W-QACGLSTQY---------------------KKGDP--
AtUGT92A1     182 ----------LDDFPEA-GEI---EKTQLNSFML--------------------EADGTDD
GbUGT92K1     184 ----------VPDLPHI--RL---HRSQLSQILA------------------VSEASDP--
GmUGT92G4     178 ----------VPGFPQN-YRF---HKTQLHRFLQ--------------------AADGTDD
PoUGT90A7     178 ----------VPTFPWIKITR---NDFDFPLNQR--------------------DPSGP--
AtUGT90A1     176 PVT-------VPDFPWI--KV---KKCDFDHGTT--------------------EPEES-G
AcUGT73J1     166 ----------MDGLAEK-IKL---YRKGLPDMFS---------------------------
MtUGT73P1     180 ----------LPGLPHN-VEM---TRLQLPDWLR--------------------APN----
SrUGT73E1     181 ----------RVVLPGL--PDRI-EVTKLQIVGS--------------------SRPANVD
CpPGT4        173 ----------YFNIPGL--PDHI-QFTKVQLLIS--------------------KRDDD--
AtUGT73C1     179 ----------IPNFPDR-VEF---TKSQLPMVLV----------------------AGD--
CpPGT2        177 ----------VPCLPDK-IEF---TTQQVDSSLG---------------------------
PjGAT         183 ----------VPGMPQR-VEI---TKAQLPGAFV----------------------TLP--
CtiUGT73AE1   175 PFI-------LPGLPDR-IEI---TKVQLPQEFN--------------------YSDLG--
CaUGT73AH1    176 ----------VPGLPDV-IEF---KRAQLPGLFN--------------------PGSNPGS
PzGAT2        177 ----------VRGLPDH-IEF---TKAQLPGLFN--------------------PGSVP--
AcUGT73G1     174 ----------LPNLPHK-IEM---RRALIPDFRK--------------------VAPS---
MtUGT73K1     173 ----------IHGLPHP-LTL---PIKPSPGFAR---------------------------
GeUGT73F1     169 ----------SFVIPGL-PHP---IAMNAAPPKQ---------------------------
AtUGT73B1     184 ----------IPDLPGD-ILITEEQVMETEEESV---------------------------
GuUGAT        173 ----------VPNLPGE-ITLTKMQLPQLPQHDK---------------------------
NmUGT73BD1    172 ----------LPNFPNQ-IEF---SKAQVPGSDL--------------------GIKD---
NmUGT73BD1      1 ---------------------------------------MTAQFHVVFFP-LM-AQGHLIP
```

```
AtUGT81A1     249 ----SVHPLMQHVPLRVLRSKGLLKKIVFTTVITDLS-TCH-PTWFH--KLV--------
AtUGT80A2     265 S---GPSEIPIQR--NQMKDIIY---SLLPAC-KEPD-PDSGISFKA---DA-IIANPPA
AtUGT80B1     228 S---GPSEISKQR--KQLKAIIE---SLLPAC-IEPD-LET-ATSFR--AQA-IIANPPA
OsZOGT3       189 ----FLDYASERA--RASESILSGAGILANAS-RALE-GDF-IDDLA--ETLAAGG----
OsZOGT1       182 -----KEFVELISRAEQDEENAASSGILMNTS-RALE-AEF-IDEIATHPMFKE------
ZmcisZOG1     182 ----SREFVDLVFRMEEEEQGAPVAGLVMNTC-RALE-GEF-LDVVAAQPPFQG------
GmUGT79A6     197 ----EKNLTGYERVLQSLGECSF---IVFKTC-KEIE-GPY-LDYIE--TQF--------
AtUGT79B6     185 DGTSFYERIMIGL--KNCDV------ISIRTC-QEME-GKF-CDFIE--NQF--------
GmUGT91H9     192 KDSDTGRMANFDL--RKAYSSCD--MFLLRTC-RELE-GEW-LDYLA--HKY--------
ZmUGT91L1     171 ----FVETFKRCK------------VIAARSS-FELE-PES-LPLMT--KIL--------
PoUGT95A1     239 PGGGPSPGPGQKP--RWVDEVDGSIALLINTC-DDLE-HVF-INYMA--EQT--------
MtUGT95B4     187 CGPSMMGPPKLGGQPPWLDEIQETIALMINTC-DDLE-HPF-INYIA--NHV--------
PgUGT95B2     221 K---FMGPPKPGQEPPWVDDVADSVALLFNTC-DDLE-GPF-IEYLA---DR-VG-----
VvUGT95B6     212 SGPKFGGPPKPGHQPPWVDETAGSIALMINTC-DDLE-RPF-IEYVA--HQT--------
AtUGT82A1     190 K---RFKFWQRTL--ERTKS-LR--WILTSSFKDEYEDVDNHKASYK---KS-NDLNKEN
VpUGT94F1     194 ---------------GSLNSSSE--IILLKTS-KELE-KKY-IDYLSFLCR---------
AtUGT87A1     180 ----VFNIFKKSF--GELYKAKY---LLFPSA-YELE-PKA-IDFFT--SKF--------
AtUGT72C1     187 ------ELAESQRIGDEVIT-AD--GVFVNTW-HSLE-QVT-IGSFL--DPENLGRVMRG
AtUGT72D1     192 ----YKECVRAGL---EVPM-SD--GVLVNTW-EELQ-GNT-LAALR--EDEELSRVMK-
LjUGT72AD1    191 ----DYKVFLESN--KRFYL-AD--GVIINSF-FDLE-PET-FRALQ--ENQGTSPPSV-
AtUGT72B1     175 ----AYKWLLHNT--KRYKEAEG---ILVNTF-FELE-PNA-IKALQ--EPGLDK-----
VvUGT1        191 ----AYKWVVHHA--KRYKTGP---GIIVNSF-MDLE-PGA-FKALKEIEPDY-------
LjUGT72AH1    191 ----AYKFYLRRC--QQLHI-TD--GILVNSF-TEME-PEA-MKAIS--QNGNGA-----
LjUGT72Z2     192 ----AYDLILKRC--KRFSLAD---GFLVNSF-LELE-EGT-VKALQEQNRGNRGENRGN
GmUGT72X4     191 ----IYNNFLERA--KAMATAD---GILINTF-LEME-PGA-IRALQEFENG--------
MtUGT72L1     198 ----AYKYFLQHV--KSLSFADG---VLVNSF-LEME-MGP-INALT--EEGSGN-----
PgUGT72BD1    195 ----HDDYVRIGR---RLAE-AD--GILINTW-ESLE-PKT-LQALR--DPKAFGRFSQ-
AtUGT72E1     184 ----YREFVPFGS---VFPT-CD--GIIVNTW-DDME-PKT-LKSLQ--DPKLLGRIAG-
OsUGT706C1    191 ----VGTTRIHHC--SRMAE-AR--GILVNSF-DWLE-TRA-LKAIR--GGLCLPSGRSV
OsUGT706D1    191 ----FYKARVKLY--ARAME-AS--GVLLNTY-EWLE-ARA-MGALR--EGA-CSPDRPT
MtUGT88E1     196 ----SYQVLLQSA--KSLRE-SD--GIIVNTF-DAIE-KKA-IKALR--NGLCVPDGTT-
VpUGT88D8     182 ----THNHFLDTA--RNMRKANG---ILVNSF-DALE-YRS-KAALL--NGICVPNGPT-
NmUGT88P1     194 ----LYKELINTA--NQMAKCSG---ILINAF-ETLE-PKA-VKALK--EGLCTPGMPT-
AtUGT88A1     195 ----VYDVFIMFG--KQLSKSSG---IIINTF-DALE-NRA-IKAIT--EELCF------
VvGT7         192 ----AYELFVNMS--IHLPRSAG---IIVNTF-EALE-PRA-VKTIL--DGLCVLDGPT-
MdUGT88F1     199 ----YSDMIYFCS--HLPK-SN--GIIVNTF-EELEPPSV-LQAIA--GGLCVPDGPT-
ScUGT5        192 ----AYHDVLYFS--HHLARSSG---IIVNTF-DGLE-PIA-LKAIT--DGLCIPDIPT-
OsUGT707A2    209 ----NYEWLVYHG--SRFMEAA---GIIVNTV-AELE-PAV-LEAIA--DGRCVPGRRV-
AtUGT71B1     188 ----WFPYVLGRA--RSFRA-TK--GILVNSV-ADME-PQA-LSFFS--GGNGNTNI---
MdUGT71A15    198 ----DMESTKSTL--NHVSKYKQTKGILVNTF-MELE-SHA-LHYLD--SGDKI------
BvUGT71F1     209 ----GVPMLLNLV--RGLRRSKG---ILVNSF-TELE-TSG-VQALL--DQATEGGSI--
Db6-GT        194 ----EKDFLVKRG--RRFRR-SN--GILVNTS-NELE-SYA-IQTLL---EQ-AKDNKI-
AtUGT71C1     208 ----TYEPWVELA--ERFPEAKG---ILVNSY-TALE-PNG-FKYFDRCPDNY-------
AtUGT71C4     202 ----NKEAYEAYV--ELAPRFADAKGILVNSF-TELE-PHP-FDYFS--HLEKF------
AtUGT71D1     199 ----YDAYVKLAI---LFTK-AN--GILVNSS-FDIE-PYS-VNHFL--QEQNY------
FaUGT71W2     190 ----YVSFASHAR---RFKE-TK--GVIINTL-VELESHAV-HSIFR--VGEGDQSDQPW
MtUGT71G1     199 ----YIAYYKLAE---RFRD-TK--GIIVNTF-SDLE-QSS-IDALY--DHDEKI-----
MdUGT71K1     199 ----LSAYIKLAS---RFRE-TR--GIIVNTF-VELE-THA-ITLFS--NDD-RV-----
AtUGT89A2     207 DLESIKDFSMNLL--------SY--GSVFNSS-EILE-DDY-LQYVK--QRMGH------
AtUGT89C1     168 ----LETATTESY-----------GLVINSF-YDLE-PEF-VETVK--TRFLNH-----
AtUGT89B1     203 ----AWEFIRDSF--RDNVA-SW--GLVVNSF-TAME-GVY-LEHLK---REMGH-----
NmUGT89P1     195 ----SWEFFKNGV--LANTQ-SW--GAIYNSF-RDLE-GVY-IDYIK--KKM-GH-----
AtUGT92A1     209 ----WSVFMKKII--PGWSD-FD--GFLFNTV-AEID-QMG-LSYFR--RIT--------
GbUGT92K1     210 ----WTLLWKRNT---SCNLSSW--GTIFNTF-EDLE-HDF-LDYFR--RIT--------
GmUGT92G4     205 ----WSRFLVPQI---QLSMKSD--GWICNTI-EKIE-PLG-LKLLR--NYL--------
PoUGT90A7     204 ----YMDFIMETV---IASANSY--GLITNSF-YELE-PLF-LDYLN--REA--------
AtUGT90A1     204 A---ALELSMDQI--KSTTT-SH--GFLVNSF-YELE-SAF-VDYNN---NS-GDK----
AcUGT73J1     186 ----NIPFLITMG---EAEAKSY--GVVVNTF-REME-PTY-VDFYK---GT--------
MtUGT73P1     203 ----GYTYLMKMI--KDSEKKSY--GSLFDSY-YEIE-GTY-EDYYK--IAM--------
SrUGT73E1     209 E---MGSWLRAVE---AEKA-SF--GIVVNTF-EELE-PEYVEEYKT--VKD--------
CpPGT4        199 ----RKELREQIL--AADKK-TY--GAIINTF-EELE-SPFIENYKK--AKQ--------
AtUGT73C1     202 ----WKDFLDGMT---EGDNTSY--GVIVNTF-EELE-PAYVRDYKK--VKA--------
CpPGT2        197 ---SRFNVFQKKM--GAADTGTY--GVIVNSF-EELE-PAYIKEYKK--IRH--------
PjGAT         206 ----GLDDIRDQM--REAESSAY--GVVVNSF-SELE-QGCSEEYKK--AIA--------
CtiUGT73AE1   203 ----TKEQLERVR--ETEAT-AY--GIVINSF-EELE-QEY-VNELK---KVKNG-----
CaUGT73AH1    203 A---NVNDIREQI--RATEVGAY--GVVINSF-EELE-HDY-VREFKKIKE---------
PzGAT2        202 ----AIDEIREQV--RATEVGAY--GVVINSF-EELE-QDY-VDEFK--KVR--------
AcUGT73G1     198 ----VFQLLIKQ---KEAEKLSY--GLIINSF-YELE-PGY-VDYFR--NVV--------
MtUGT73K1     193 ----LTESLIEAE--------NDSHGVIVNSF-AELD-EGY-TEYYE--NLT--------
GeUGT73F1     189 ----MSDFLESML--ETELK-SH--GLIVNNF-AELDGEEY-IEHYE--KTT--------
AtUGT73B1     207 ----MGRFMKAIR---DSERDSF--GVLVNSF-YELE-QAY-SDYFK--SFV--------
GuUGAT        196 ----VFTQLLEES--NESELKSF--GVIVNSF-YELE-PVY-ADHYR--NEL--------
NmUGT73BD1    196 ----GATELFKQI--NEADITSY--GIIINSF-NELE-KDY-VDYYR--NVC--------
NmUGT73BD1      1 -------------------------------------MTAQFHVVFFP-LM-AQGHLIP
```

```
NtTOGT1       196 ----AMTRMIKTV--RESDSKSY--GVVFNSF-YELE-TDY-VEHYT--KVL--------
BvUGT73A4     200 ----DSDYKERSKAIKESELKCY--GVLVNSF-YELE-PDY-AEYFR--KDL--------
Db5-GT        203 ----HFTKRTERI--RELERKSY--GVIVNSF-YELE-PDY-ADFLR--KEL--------
AtUGT86A1     209 TNTVVYRILFKAF--KDVKRADF---VVCNTV-QELE-PDS-LSALQ---AK--------
ZmUFGT1       208 ----YVINLLVHRMGQCLPRSAA--AVALNTF-PGLDPPDV-TAALA--EIL--------
CteUGT78K6    191 L---FSRTLNSLG--VVLPQAKA---VVVNFF-AELDPPLF-VKYMR--SKL--------
AtUGT78D1     198 ----FPKALYQMS--LALPRASA---VFISSF-EELE-PTL-NYNLR--SKL--------
CoUGT78B3     198 L---FARLLHKAA--QVMPQ-AD--VVVINSF-EELE-QDI-VDHLK--TKF--------
MtUGT78G1     202 ----FATMLHKMG--LELPRANA---VAINSF-ATIH-PLI-ENELN--SKF--------
LgUGT78J1     193 ----LAVTINKAV--EKLPR-SH--AVILNSF-EEID-PII-AKDLK--SKF--------
CpPGT11       193 MQKIFFDFMVQNM--RATRAVDF---QLCNST-YELE-GGA-FSMI--------------
AtUGT83A1     210 N---IFQLMLQNN--NSIESTDW---LLCNSV-HELETAAFGLG----------------
FaUGT75T1     190 S---ILPTFQEHY--QIWEK-SPNSCVLVNTF-DGLE-EAA-IRDMR--AHM--------
AtUGT75C1     191 ----LPSALVTLREHIEALETESNPKILVNTF-SALE-HDA-LTSVE-------------
AtUGT75B1     181 AYDAFQEMMEFLI--KETKP-----KILINTF-DSLE-PEA-LTAFP--NI---------
AtUGT75D1     207 F---LLPAFREQI--DSLKE-EINPKILINTF-QELE-PEA-MSSVP--DNF--------
ZmUGT74A1     191 ----YPTIAMQAI--KQFAHAGKDDWVLFNSF-EELE-TEV-LAGLT---KY--------
AsUGT74H5     181 ----YQDMLLSQF--VGLDAVDH---VLVNSF-HELQ-PQE-SAYME---ST--------
BdUGT74J7     200 ----FLDAVVGQF--EGLEDADD---VFVNSF-HDLE-PKE-ADYLS---ST--------
SrUGT74G1     190 P---WSQMLFGQF--ANIDQ-AR--WVFTNSF-YKLE-EEV-IEWTR---KI--------
AtUGT74B1     189 E---HGRVLLNQF--PNHENADW---LFVNGF-EGLEETQD-CENGE--SDA--------
RsUGT74R1     182 ----LLELLVDQF--SNLDEADY---VFFNTF-DKLE-NQM-VEWMA---RQ--------
LuUGT74S1     187 ----HTEFLAAHL--RQFSN-DGADWVFCNTV-YQLE-LEA-VDWLT---KQWL------
AtUGT74F1     179 A---YFEMVLQQF--TNFDK-AD--FVLVNSF-HDLD-LHE-EELLS---KV--------
SgUGT74AC1    185 ----IIDLLTSQY--SNIQDANL---LFCNTF-DKLE-GEI-IQWME--TLG--------
AtUGT74C1     187 L---LHEFVVRQF--SNLLQAD---CILCNTF-DQLE-PKV-VKWMN---DQ--------
AtUGT74D1     187 ----LFELISSQF--VNVDDIDF---FLVNSF-DELE-VEV-LQWMK---NQ--------
AtUGT74E2     186 N---ILRIVVDQL--SNIDRVDL---VLCNTF-DTLE-EKL-LKWVQ---SL--------
AtUGT84B1     186 ----FYNLMAEFA--DCLRYVKW---VLVNSF-YELE-SEI-IESMA---DL--------
DgpHBAGT      201 RV--LKDVILGQF--KNLEK-TF--CVLVDSF-EELE-RES-LKPLL--SSA--------
MtUGT84F1     184 --PFCRYPILGTLTTAQIKDMSKVFCVLVDTF-EELE-HDF-IDYIS--EKS--------
GtUF6CGT1     202 F---FGTNILGQF--KNLSK-NF--CILMDTF-YELE-HEI-IDNMC---KL--------
PgUGT84A23    198 F---LRRAILGQY--KNLEK-PF--CILMDTF-QELE-HEI-IEYTS---KI--------
PgUGT84A24    197 F---LRRAIMGQY--KNLDK-PF--CVLMDTF-QELE-HEI-IEYMS---KI--------
AtUGT76E1     187 ----LESILKVYSETVNIRTASA---VIINST-SCLE-SSS-LAWLQ--KQL--------
AtUGT76D1     180 ---GSMERLMILY--ENVSNRASSSGIIHNSS-DCLE-NSF-ITTAQ--EKW--------
SrUGT76G1     196 ----NWQILKEIL-GKMIKQTKASSGVIWNSF-KELEESEL-ETVIR--EIP--------
VvGT15        220 ----FFQILVNMY------KKKFSSPIIWNTM-DCLE-QSS-LTQRQ--QQL--------
AtUGT76C1     194 ----LDAYLLKIL---DATKPAS--GIIVMSC-KELD-HDSLAESNK--VFS--------
AtUGT76B1     185 ----GDKLQIGVM--KSLKSSS---GIIFNAI-EDLE-TDQ-LDEAR--IEF--------
CsUGT76F1     187 ----LYEIVNGMVDGAKVSS-----GIIWNTF-EDLE-ESA-LATLR--QQF--------
OsUGT709A4    207 DVHPKLQMLVDLTTGSCKAR-----AVILNTA-ASLE-APA-LAHIA--PRV--------
SrUGT85C2     207 ----VLMFTTEAP--QRSHKVSH---HIFHTF-DELE-PSI-IKTLS--LRY--------
CoUGT85N1     209 I---MFNCKIIEV--NSAFK-AK--GVILNTF-DDLE-QEV-LDAIK--SKI--------
MeUGT85K4     211 ----MFDFLGSEA--HKSLKADA---IILNTF-DELE-QEV-LDAIA--ARY--------
AtUGT85A1     214 ----MISFALRET--ERAKRASA---IILNTF-DDLE-HDV-VHAMQ--SIL--------
MtUGT85H2     212 ----MLEFFIEVA--DRVNKDTT---ILLNTF-NELE-SDV-INALS--STI--------
MdUGT71K1     199 ----LSAYIKLAS---RFRE-TR--GIIVNTF-VELE-THA-ITLFS--NDD-RV-----
AtUGT89A2     207 DLESIKDFSMNLL--------SY--GSVFNSS-EILE-DDY-LQYVK--QRMGH------
AtUGT89C1     168 ----LETATTESY-----------GLVINSF-YDLE-PEF-VETVK--TRFLNH-----
AtUGT89B1     203 ----AWEFIRDSF--RDNVA-SW--GLVVNSF-TAME-GVY-LEHLK---REMGH-----
NmUGT89P1     195 ----SWEFFKNGV--LANTQ-SW--GAIYNSF-RDLE-GVY-IDYIK--KKM-GH-----
AtUGT92A1     209 ----WSVFMKKII--PGWSD-FD--GFLFNTV-AEID-QMG-LSYFR--RIT--------
GbUGT92K1     210 ----WTLLWKRNT---SCNLSSW--GTIFNTF-EDLE-HDF-LDYFR--RIT--------
GmUGT92G4     205 ----WSRFLVPQI---QLSMKSD--GWICNTI-EKIE-PLG-LKLLR--NYL--------
PoUGT90A7     204 ----YMDFIMETV---IASANSY--GLITNSF-YELE-PLF-LDYLN--REA--------
AtUGT90A1     204 A---ALELSMDQI--KSTTT-SH--GFLVNSF-YELE-SAF-VDYNN---NS-GDK----
AcUGT73J1     186 ----NIPFLITMG---EAEAKSY--GVVVNTF-REME-PTY-VDFYK---GT--------
MtUGT73P1     203 ----GYTYLMKMI--KDSEKKSY--GSLFDSY-YEIE-GTY-EDYYK--IAM--------
SrUGT73E1     209 E---MGSWLRAVE---AEKA-SF--GIVVNTF-EELE-PEYVEEYKT--VKD--------
CpPGT4        199 ----RKELREQIL--AADKK-TY--GAIINTF-EELE-SPFIENYKK--AKQ--------
AtUGT73C1     202 ----WKDFLDGMT---EGDNTSY--GVIVNTF-EELE-PAYVRDYKK--VKA--------
CpPGT2        197 ---SRFNVFQKKM--GAADTGTY--GVIVNSF-EELE-PAYIKEYKK--IRH--------
PjGAT         206 ----GLDDIRDQM--REAESSAY--GVVVNSF-SELE-QGCSEEYKK--AIA--------
CtiUGT73AE1   203 ----TKEQLERVR--ETEAT-AY--GIVINSF-EELE-QEY-VNELK---KVKNG-----
CaUGT73AH1    203 A---NVNDIREQI--RATEVGAY--GVVINSF-EELE-HDY-VREFKKIKE---------
PzGAT2        202 ----AIDEIREQV--RATEVGAY--GVVINSF-EELE-QDY-VDEFK--KVR--------
AcUGT73G1     198 ----VFQLLIKQ---KEAEKLSY--GLIINSF-YELE-PGY-VDYFR--NVV--------
MtUGT73K1     193 ----LTESLIEAE-------NDSHGVIVNSF-AELD-EGY-TEYYE--NLT--------
GeUGT73F1     189 ----MSDFLESML--ETELK-SH--GLIVNNF-AELDGEEY-IEHYE--KTT--------
AtUGT73B1     207 ----MGRFMKAIR---DSERDSF--GVLVNSF-YELE-QAY-SDYFK--SFV--------
GuUGAT        196 ----VFTQLLEES--NESELKSF--GVIVNSF-YELE-PVY-ADHYR--NEL--------
NmUGT73BD1    196 ----GATELFKQI--NEADITSY--GIIINSF-NELE-KDY-VDYYR--NVC--------
NmUGT73BD1      1 -----------------------------------MTAQFHVVFFP-LM-AQGHLIP
```

```
AtUGT81A1     293 -------------TRCYC--PSTEVAKRAQKAGLETSQIKVYGLPVRPSFVKPVRPKVEL
AtUGT80A2     311 YGHTHVAEALKIPIHVFFTMP-WTPTSEFPHPLSRVKQPA------GYRLSYQIVDSLIW
AtUGT80B1     274 YGHVHVAEALGVPIHIFFTMP-WTPTNEFPHPLARVPQSA------AYWLSYIVVDLMVW
OsZOGT3       234 -------------KKLFAIGP-LNPLLNTGSSEQGRRRHE----------------CLDW
OsZOGT1       228 -------------LKLFAAGP-LNPLLDATARTPGQTRHE----------------CMDW
ZmcisZOG1     229 -------------QRFFAVGP-LNPLLLDADAPTTPPGQA-------------RHECLEW
GmUGT79A6     237 ------------RKPVLLSGP-LVPEPSTDVLEEK---------------------WSKW
AtUGT79B6     224 ------------QRKVLLTGP-MLPEPDNSKPLEDQWRQ--------------------W
GmUGT91H9     235 ------------KVPVVPVGL-VPPSIQIRDVEEEDNNPD-------------WVKIKDW
ZmUGT91L1     202 ------------GKPVIPVGL-LPPAPAGGNTQRDDSAA------------------LSW
PoUGT95A1     284 ------------KLPVWGVGP-LLPEQFWKSAGELLHDHE---MRSNHKSNYTEDEVVQW
MtUGT95B4     234 ------------KKPVCGVGP-LLPGQYWKSSGSIIHDRD---FRSNRLSNITEEEVIQW
PgUGT95B2     266 -------------KPVWGVGP-LLPEQYWRSAGSLLHDRE---IRTNRKSSITEDEVIQW
VvUGT95B6     259 ------------GIPVWGVGP-LLPDQYWKSSGSLLHDRD---IRPNKKSSCTEEEVIQW
AtUGT82A1     238 NGQN---------PQILHLGP-LHNQEATNNITITKTSF-----------WEEDMSCLGW
VpUGT94F1     225 -------------KQIIPTGL-LIANSDEKDEPE----------------------IMQW
AtUGT87A1     218 ------------DFPVYSTGP-LIPLEELSVGNENRELD-----------------YFKW
AtUGT72C1     233 -------------VPVYPVGP-LVRPAEPGLKHG----------------------VLDW
AtUGT72D1     236 -------------VPVYPIGP-IVRTNQHVDKPNS---------------------IFEW
LjUGT72AD1    236 -------------PHVYPVGP-FIVQKESYDESHGNEDET--------------DEYIRW
AtUGT72B1     216 -------------PPVYPVGP-LVNIGKQEAKQTEESE------------------CLKW
VvUGT1        232 -------------PPVYPVGP-LTRSGSTNGDDGSE--------------------CLTW
LjUGT72AH1    232 -------------PQVHPIGP-ITQTWSNNNKNCSE--------------------CLLW
LjUGT72Z2     240 RGKNRGNRD----SPVFLVGP-VIQTGPSSEPKGSESEY-----------------CVRW
GmUGT72X4     231 ------------KIRLYPVGP-ITQKGASNEADESDK-------------------CLRW
MtUGT72L1     239 -------------PSVYPVGP-IIQTVTGSVDDANGLE------------------CLSW
PgUGT72BD1    239 -------------VRILPIGP-LVSGFQSFGSSQPKDD------------------ILQW
AtUGT72E1     228 -------------VPVYPIGP-LSRPVDPSKTNHP---------------------VLDW
OsUGT706C1    237 -------------PAIYCVGP-LVDGGKLKENDAR-------------------HECLEW
OsUGT706D1    236 -------------PPVYCVGP-LVASGEEEGGVR--------------------HACLAW
MtUGT88E1     241 -------------PLLFCIGP-VVSTSCEEDKSG----------------------CLSW
VpUGT88D8     227 -------------PQVLFVAP-LVTGMNSRKGDSE-------------------HECLSW
NmUGT88P1     239 -------------PPVYCIGP-LIASGDKGNVNDAHGHEI-----------------LTW
AtUGT88A1     235 -------------RNIYPIGP-LIVNGRIEDRNDNKAVS-----------------CLNW
VvGT7         237 -------------SPIFCIGP-LIAADDRSGGGGGGGGGS------------GIPECLTW
MdUGT88F1     244 -------------PPVYYVGP-LIEEEKELSKDADAAEKE---------------DCLSW
ScUGT5        237 -------------PPIYNIGP-LIADADTKPADQNLKHHS-----------------LSW
OsUGT707A2    254 -------------PAIYTVGP-VLSFKTPPEKPHE---------------------CVRW
AtUGT71B1     231 -------------PPVYAVGP-IMDLESSGDEEKRKE-------------------ILHW
MdUGT71A15    241 -------------PPVYPVGP-LLNLKSSDEDKASD--------------------ILRW
BvUGT71F1     253 -------------PAIYPVGP-ILELDSGSQGEDHVS-------------------ILQW
Db6-GT        237 -------------PPVYPVGP-ILELNSKSRCGTKEDEEV---------------SIMRW
AtUGT71C1     249 -------------PTIYPIGP-ILCSNDRPNLDSSERDRI-----------------ITW
AtUGT71C4     245 -------------PPVYPVGP-ILSLKDRASPNEEAVDR---------------DQIVGW
AtUGT71D1     238 -------------PSVYAVGP-IFDLKAQPHPEQDLTRRD---------------ELMKW
FaUGT71W2     236 -------------PAVYPVGP-LIDTKGEHQVRSDRDR------------------IMEF
MtUGT71G1     239 -------------PPIYAVGP-LLDLKGQPNPKLDQAQHD---------------LILKW
MdUGT71K1     238 -------------PPVYPVGP-VIDLDDGQEHSNLDQAQR--------------DKIIKW
AtUGT89A2     246 -------------DRVYVIGP-LCSIGSGLKSNSGSVDPS----------------LLSW
AtUGT89C1     202 -------------HRIWTVGP-LLPFKAGVDRGGQSSIPP--------------AKVSAW
AtUGT89B1     243 -------------DRVWAVGP-IIPLSGDNRGGPTSVSVD---------------HVMSW
NmUGT89P1     235 -------------GRVWAVGP-LLPANDASKRGGSCVMPI--------------DDVMTW
AtUGT92A1     247 ------------GVPVWPVGP-VLKSPDKKVGSRSTEEA-----------------VKSW
GbUGT92K1     248 ------------GRPVWPVGP-ILPLTGTLKTKKIAWRGN--------ESAVDAETCLQW
GmUGT92G4     243 ------------QLPVWAVGP-LLPPASLMGSKHRSGKE----------TGIALDACMEW
PoUGT90A7     242 ------------KPKAWCVGP-LCLAADHGSDHKPKW--------------------VEW
AtUGT90A1     245 -------------PKSWCVGP-LCLTDPPKQGSAKPA-------------------WIHW
AcUGT73J1     223 -------------KKAWCIGP-LSLANKLDEEKTAGWIAE---------KEEVKEKIVKW
MtUGT73P1     242 ------------GSKSWSVGP-VSLWMNKDDSDKAGRGHG--------KEEDEEEGVLKW
SrUGT73E1     248 -------------KKMWCIGP-VSLCNKTGPDLAERGNKA----------AITEHNCLKW
CpPGT4        238 -------------GKVWRIGP-ASLCNKEPIDKAERGRTA----------SIDVPECLTW
AtUGT73C1     241 -------------GKIWSIGP-VSLCNKLGEDQAERGNKA----------DIDQDECIKW
CpPGT2        238 -------------DKVWCIGP-VSLSNKEYSDKAQRGNKA----------SVDEHQCLKW
PjGAT         246 -------------KKVWCIGP-VSLCNKDNLDKFERGNKA----------SIDETLCTEW
CtiUGT73AE1   243 --------------KVWCLGP-LSLTNNNDLGKSVRGNNS----------SIDEQRIVKW
CaUGT73AH1    244 -------------GKVWCVGP-LSLCNTDDIDKAQRGNNV---------AMDQQHKCLQW
PzGAT2        241 ------------RDKVWCVGP-LSLNNENMLDKAQRGHNN---------ASIDGNKCLQW
AcUGT73G1     236 ------------GRKAWHVGP-LLLNDKNVNTFDRGSKSA-----------IDEASCLSW
MtUGT73K1     228 ------------GRKVWHVGP-TSLMVEIPKKKKVVSTEN--------DSSITKHQSLTW
GeUGT73F1     228 ------------GHRAWHLGP-VSLIRRTSQEKAERGEKS----------VVSVHECLSW
AtUGT73B1     245 ------------AKRAWHIGP-LSLGNRKFEEKAERGKKA----------SIDEHECLKW
GuUGAT        235 ------------GRRAWHLGP-VSLCSRDTEEKSRRGREA----------AIDENECLKW
NmUGT73BD1    235 ------------GRRAWLLGP-LSLINRNKDKTLIDEHE-----------------CLKW
NmUGT73BD1      1 -------------------------------------MTAQFHVVFFP-LM-AQGHLIP
```

```
NtTOGT1      235 ------------GRRAWAIGP-LSMCNRDIEDKAERGKKS----------SIDKHECLKW
BvUGT73A4    241 ------------GRRAWNIGP-VSLYNRSNEEKAQRGKQA----------SIDEHECLKW
Db5-GT       242 ------------GRRAWHIGP-VSLCNRSIEDKAQRGRQT----------SIDEDECLKW
AtUGT86A1    250 -------------QPVYAIGP-VFSTDSVVPTSLWAESD-----------------CTEW
ZmUFGT1      250 -------------PNCVPFGP-YHLLLAEDDADTAAPADP--------------HGCLAW
CteUGT78K6   231 -------------QSLLYVVP-LPCPQLLLPEIDSNG-------------------CLSW
AtUGT78D1    236 -------------KRFLNIAP-LTLLSSTSEKEMRDPHG-----------------CFAW
CoUGT78B3    237 -------------QTCLTVGP-FTIVAPSISDQHDPHG------------------CLPW
MtUGT78G1    240 -------------KLLLNVGP-FNLTTPQRKVSDEHG-------------------CLEW
LgUGT78J1    231 -------------RHFLNIGP-SILPSPIADDKSG---------------------CLSW
CpPGT11      231 -------------PELLPIGP-LLASNRLGNSAGYFLPED--------------SKCVEW
AtUGT83A1    245 -------------PNIVPIGP-IGWAHSLEEGSTSLGSFL-----------PHDRDCLDW
FaUGT75T1    231 --------------NLITVGP-LIRSAEVCCDLFDKSGDD----------------YLQW
AtUGT75C1    231 ------------KLKMIPIGP-LVSSSEGKTDLFKSSDED----------------YTKW
AtUGT75B1    220 --------------DMVAVGP-LLPTEIFSGSTNKSVKDQ-------------SSSYTLW
AtUGT75D1    248 --------------KIVPVGP-LLTLRTDFSSRGE---------------------YIEW
ZmUGT74A1    231 -------------LKARAIGP-CVPLPTAGRTAGANGRIT-----YGANLVKPEDACTKW
AsUGT74H5    218 -------------WGAKTVGP-TVPSAYLDKRITDDVSYG------FHLYTPMTATTKAW
BdUGT74J7    237 -------------WRVKTIGP-TLPSFYLDDDRLPSNKTY------GFDLFDSTAPCMAW
SrUGT74G1    228 -------------WNLKVIGP-TLPSMYLDKRLDDDKDNG------FNLYKANHHECMNW
AtUGT74B1    229 -------------MKATLIGP-MIPSAYLDDRMEDDKDYG------ASLLKPISKECMEW
RsUGT74R1    219 -------------WQVLTVGP-TIPSMYLDKCVKDDRSYG------LNLFKPNRESCRDW
LuUGT74S1    228 -------------INFRTIGP-TIPSFYLDKQIPDDKDYD------ISIFNPQNQTCMNW
AtUGT74F1    217 -------------CPVLTIGP-TVPSMYLDQQIKSDNDYD-----LNLFDLKEAALCTDW
SgUGT74AC1   223 -------------RPVKTVGP-TVPSAYLDKRVENDKHYG------LSLFKPNEDVCLKW
AtUGT74C1    225 -------------WPVKNIGP-VVPSKFLDNRLPEDKDYE-----LENSKTEPDESVLKW
AtUGT74D1    224 -------------WPVKNIGP-MIPSMYLDKRLAGDKDYG------INLFNAQVNECLDW
AtUGT74E2    224 -------------WPVLNIGP-TVPSIYLDKRLSEDKNYG------FSLFNAKVVECMEW
AtUGT84B1    223 -------------KPVIPIGP-LVSPFLLGDGEEETLDGK------NLDFCKSDDCCMEW
DgpHBAGT     241 -------------PIIRPIGP-LYKWKEEKDVRGDMWSAA--------------EECIEW
MtUGT84F1    229 -------------IAIRPVGP-LFKNPKANGASNNILG--------DFTKSNDDCNIIEW
GtUF6CGT1    240 -------------CPIKPIGP-LFKIPKDPSSNGITGNFM------------KVDDCKEW
PgUGT84A23   236 -------------CPIKTVGP-LFKNPKAPNTTVKGDFMK-------------ADDCIGW
PgUGT84A24   235 -------------CPIKTVGP-LFKNPKAPNANVRGDFMK-------------ADDCISW
AtUGT76E1    227 ------------QVPVYPIGP-LHIAASAPSSLLEEDRS-----------------CLEW
AtUGT76D1    222 ------------GVPVYPVGP-LHMTNSAMSCPSLFEEER---------------NCLEW
SrUGT76G1    239 -------------APSFLI-P-LPKHLTASSSSLLDHDRT----------------VFQW
VvGT15       257 ------------QVPFFPIGP-LHKLAPPSSSSLLEEDSS----------------CITW
AtUGT76C1    233 -------------IPIFPIGP-FHIHDVPASSSSLLEPD---------------QSCIPW
AtUGT76B1    223 ------------PVPLFCIGP-FHRYVSASSSSLLAHDMT----------------CLSW
CsUGT76F1    225 ------------SIPIFPIGP-FHICIPASPSSLLTQDQS----------------CIAW
OsUGT709A4   249 -------------RDVFAVGP-LHAMSPAPAAATSLWRED--------------DGCMAW
SrUGT85C2    245 -------------NHIYTIGP-LQLLLDQIPEEKKQTGIT---SLHGYSLVKEEPECFQW
CoUGT85N1    248 -------------PQLYTIGP-LSMLCDHMLQPDSKLCEA--------SLWEEDTSCLEW
MeUGT85K4    249 ------------SKNIYTVGP-FILLEKGIPEIKSKAFRS--------SLWKEDLSCLEW
AtUGT85A1    252 -------------PPVYSVGP-LHLLANREIEEGSEIGMM------SSNLWKEEMECLDW
MtUGT85H2    250 -------------PSIYPIGP-LPSLLKQTPQIHQLDSLD-------SNLWKEDTECLDW
MdUGT71K1    238 -------------PPVYPVGP-VIDLDDGQEHSNLDQAQR--------------DKIIKW
AtUGT89A2    246 -------------DRVYVIGP-LCSIGSGLKSNSGSVDPS----------------LLSW
AtUGT89C1    202 -------------HRIWTVGP-LLPFKAGVDRGGQSSIPP--------------AKVSAW
AtUGT89B1    243 -------------DRVWAVGP-IIPLSGDNRGGPTSVSVD---------------HVMSW
NmUGT89P1    235 -------------GRVWAVGP-LLPANDASKRGGSCVMPI--------------DDVMTW
AtUGT92A1    247 ------------GVPVWPVGP-VLKSPDKKVGSRSTEEA-----------------VKSW
GbUGT92K1    248 ------------GRPVWPVGP-ILPLTGTLKTKKIAWRGN--------ESAVDAETCLQW
GmUGT92G4    243 ------------QLPVWAVGP-LLPPASLMGSKHRSGKE----------TGIALDACMEW
PoUGT90A7    242 ------------KPKAWCVGP-LCLAADHGSDHKPKW--------------------VEW
AtUGT90A1    245 -------------PKSWCVGP-LCLTDPPKQGSAKPA-------------------WIHW
AcUGT73J1    223 -------------KKAWCIGP-LSLANKLDEEKTAGWIAE---------KEEVKEKIVKW
MtUGT73P1    242 ------------GSKSWSVGP-VSLWMNKDDSDKAGRGHG--------KEEDEEEGVLKW
SrUGT73E1    248 -------------KKMWCIGP-VSLCNKTGPDLAERGNKA----------AITEHNCLKW
CpPGT4       238 -------------GKVWRIGP-ASLCNKEPIDKAERGRTA----------SIDVPECLTW
AtUGT73C1    241 -------------GKIWSIGP-VSLCNKLGEDQAERGNKA----------DIDQDECIKW
CpPGT2       238 -------------DKVWCIGP-VSLSNKEYSDKAQRGNKA----------SVDEHQCLKW
PjGAT        246 -------------KKVWCIGP-VSLCNKDNLDKFERGNKA----------SIDETLCTEW
CtiUGT73AE1  243 --------------KVWCLGP-LSLTNNNDLGKSVRGNNS----------SIDEQRIVKW
CaUGT73AH1   244 -------------GKVWCVGP-LSLCNTDDIDKAQRGNNV---------AMDQQHKCLQW
PzGAT2       241 ------------RDKVWCVGP-LSLNNENMLDKAQRGHNN---------ASIDGNKCLQW
AcUGT73G1    236 ------------GRKAWHVGP-LLLNDKNVNTFDRGSKSA-----------IDEASCLSW
MtUGT73K1    228 ------------GRKVWHVGP-TSLMVEIPKKKKVVSTEN--------DSSITKHQSLTW
GeUGT73F1    228 ------------GHRAWHLGP-VSLIRRTSQEKAERGEKS----------VVSVHECLSW
AtUGT73B1    245 ------------AKRAWHIGP-LSLGNRKFEEKAERGKKA----------SIDEHECLKW
GuUGAT       235 ------------GRRAWHLGP-VSLCSRDTEEKSRRGREA----------AIDENECLKW
NmUGT73BD1   235 ------------GRRAWLLGP-LSLINRNKDKTLIDEHE-----------------CLKW
NmUGT73BD1     1 --------------------------------------MTAQFHVVFFP-LM-AQGHLIP
```

```
AtUGT81A1     338 R-------RELGMDENLPAVLLMG---GGEGMGPIE---ATA---------RALADALYD
AtUGT80A2     364 LGIRDMVNDLRKKKLKLRPVTYLSGTQGSGS-NIPH---GYMWSPHLVPKPKDWGPQI--
AtUGT80B1     327 WSIRTYINDFRKRKLNLAPIAYFSTYHGSIS-HLPT---GYMWSPHVVPKPSDWGPLV--
OsZOGT3       264 L-------DRQ--PPD--SVLYVS--FGTTC-SLRV---EQV---------AELAATL--
OsZOGT1       258 L-------DKQ--PLA--SVLYVS--FGTTS-SLRG---DQV---------AELAAAL--
ZmcisZOG1     262 L-------DRQ--PPE--SVLYVS--FGTTS-CLHA---DQV---------AELAAAL--
GmUGT79A6     263 L-------DGF--PAK--SVILCS--FGSET-FLSD---YQI---------KELASGL--
AtUGT79B6     251 L-------SKF--DPG--SVIYCA--LGSQI-ILEK---DQF---------QELCLGM--
GmUGT91H9     269 L-------DKQ--ESS--SVVYIG--FGSEL-RLSQ---QDV---------TELAHGI--
ZmUGT91L1     231 L-------DEQ--PSK--SVVYVA--FGSEY-PMTV---KQL---------HEIARGL--
PoUGT95A1     328 L-------ESK--PRE--SVIYIS--FGSEV-GPTI---EEY---------KELAKAL--
MtUGT95B4     278 L-------DLK--PRS--SVLYVS--FGTEV-SPTM---EEY---------TELAQAM--
PgUGT95B2     309 L-------DSK--PSG--SVLYVS--FGSEV-GPTM---EEY---------PQLAQAL--
VvUGT95B6     303 L-------DSK--PRG--SVLYVS--FGSEV-GPTM---EGY---------AQLALAL--
AtUGT82A1     277 L-------QEQ--NPN--SVIYIS--FGSWVSPIGE---SNI---------QTLALAL--
VpUGT94F1     249 L-------DEK--SER--STVYIS--FGSEC-FLSK---EQI---------EEVAKGL--
AtUGT87A1     248 L-------DEQ--PES--SVLYIS--QGSFL-SVSE---AQM---------EEIVVGV--
AtUGT72C1     257 L-------DLQ--PKE--SVVYVL--LGVVG-ALTF---EQT---------NELAYGL--
AtUGT72D1     261 L-------DEQ--RER--SVVFVC--LGSGG-TLTF---EQT---------VELALGL--
LjUGT72AD1    268 L-------EKQ--EPN--SVLYIS--FGSAG-TLTH---DQI---------NELALGL--
AtUGT72B1     244 L-------DNQ--PLG--SVLYVS--FGSGG-TLTC---EQL---------NELALGL--
VvUGT1        258 L-------DHQ--PSG--SVLFVS--FGSGG-TLSQ---EQI---------TELALGL--
LjUGT72AH1    258 L-------GQQ--PPK--SVLYVS--FGSGG-TLSQ---DQI---------NELALGL--
LjUGT72Z2     278 L-------ENQ--IPK--SVLYVS--FGSGG-TLSQ---EQM---------NELAFGL--
GmUGT72X4     259 L-------DKQ--PPC--SVLYVS--FGSGG-TLSQ---NQI---------NELASGL--
MtUGT72L1     267 L-------DKQ--QSC--SVLYVS--FGSGG-TLSH---EQI---------VELALGL--
PgUGT72BD1    267 L-------DDQ--PTE--SVLYVS--FGSGG-TLSA---DQL---------TELAWGL--
AtUGT72E1     253 L-------NKQ--PDE--SVLYIS--FGSGG-SLSA---KQL---------TELAWGL--
OsUGT706C1    264 L-------DRQ--PKQ--SVVFLC--FGSRG-TFSV---SQL---------SEMARGI--
OsUGT706D1    263 L-------DAQ--PAR--SVVFLC--FGSMG-SFSA---AQL---------KEIARGL--
MtUGT88E1     265 L-------DSQ--PGQ--SVVLLS--FGSLG-RFSK---AQI---------NQIAIGL--
VpUGT88D8     254 L-------DSQ--PSK--SVIFLC--FGRKG-FFSK---QQL---------QEIATGL--
NmUGT88P1     268 L-------NSQ--PSK--SVVFLC--FGSLG-TFKE---DQL---------KEIAIGL--
AtUGT88A1     264 L-------DSQ--PEK--SVVFLC--FGSLG-LFSK---EQV---------IEIAVGL--
VvGT7         271 L-------ESQ--PKR--SVLFLC--FGSLG-LFSE---EQL---------KEIAVGL--
MdUGT88F1     275 L-------DKQ--PSR--SVLFLC--FGSMG-SFPA---AQL---------KEIANGL--
ScUGT5        266 L-------DRQ--PNQ--SVVFLC--FGSRG-SFST--DQL---------KEIAKGL--
OsUGT707A2    279 L-------DAQ--PRA--SVVFLC--FGSMG-SFAP--PQV---------LEIAAGL--
AtUGT71B1     258 L-------KEQ--PTK--SVVFLC--FGSMG-GFSE--EQA---------REIAVAL--
MdUGT71A15    267 L-------DDQ--PPF--SVVFLC--FGSMG-SFGE--AQV---------KEIACAL--
BvUGT71F1     280 L-------DKQ--PSS--SVVFLC--FGSMG-SFDA--NEV---------KEIANGL--
Db6-GT        268 L-------DEQ--PVN--SVLFVC--FGSMG-TFDE--DQV---------KEIANGL--
AtUGT71C1     278 L-------DDQ--PES--SVVFLC--FGSLK-NLSA--TQI---------NEIAQAL--
AtUGT71C4     276 L-------DDQ--PES--SVVFLC--FGSRG-SVDE--PQV---------KEIARAL--
AtUGT71D1     269 L-------DDQ--PEA--SVVFLC--FGSMA-RLRG--SLV---------KEIAHGL--
FaUGT71W2     264 L-------DNQ--PPK--SVVFLC--FGSFG-SFDE--AQL---------REIAIGL--
MtUGT71G1     270 L-------DEQ--PDK--SVVFLC--FGSMGVSFGP--SQI---------REIALGL--
MdUGT71K1     270 L-------DDQ--PQK--SVVFLC--FGSMG-SFGA--EQV---------KEIAVGL--
AtUGT89A2     276 L-------DGS--PNG--SVLYVC--FGSQK-ALTK--DQC---------DALALGL--
AtUGT89C1     234 L-------DSC--PED-NSVVYVG--FGSQI-RLTA--EQT---------AALAAAL--
AtUGT89B1     274 L-------DAR--EDN--HVVYVC--FGSQV-VLTK--EQT---------LALASGL--
NmUGT89P1     267 L-------DTKTNSDN--SVVYVC--FGSRV-ELTT--EQL---------DSLAAAL--
AtUGT92A1     277 L-------DSK--PDH--SVVYVC--FGSMN-SILQ---THM---------LELAMAL--
GbUGT92K1     287 L-------DSH--PAS--SVLYIS--FGSQN-SISV---SQM---------RELSLGL--
GmUGT92G4     280 L-------DSK--DEN--SVLYIS--FGSLH-TINA---SQM---------MALAEGL--
PoUGT90A7     269 L-------DQKLAQGC--SVLYVA--FGSQA-EIST---KQL---------EAISKGL--
AtUGT90A1     272 L-------DQK--REEGRPVLYVA--FGTQA-EISN---KQL---------MELAFGL--
AcUGT73J1     260 L-------DGK--EEG--SVLYVC--FGSLC-HFSG---GQL---------RELALGL--
MtUGT73P1     281 L-------DSK--KYD--SVLYVS--FGSMN-KFPT---PQL---------VEIAHAL--
SrUGT73E1     284 L-------DER--KLG--SVLYVC--LGSLA-RISA---AQA---------IELGLGL--
CpPGT4        274 L-------DSQ--QPS--SVVYVC--LGSIC-NLPS---SQL---------IELGLGL--
AtUGT73C1     277 L-------DSK--EEG--SVLYVC--LGSIC-NLPL---SQL---------KELGLGL--
CpPGT2        274 L-------DSK--APK--SVVYAC--LGSLC-NLIP---SQM---------RELGLGL--
PjGAT         282 L-------DSM--KPK--SVIYAC--LGSQC-RLVP---AQL---------MELGLAL--
CtiUGT73AE1   278 L-------DSR--EPG--SVIYAC--FGSSS-RVIP---QQL---------IELGLGL--
CaUGT73AH1    281 L-------DAE--KNG--SVIYVC--LGSLS-SLSNLSGAQI---------IELALGL--
PzGAT2        279 L-------DNW--ANG--SVIYAC--LGSIS-SLTC---TQL---------MELALGL--
AcUGT73G1     272 L-------GKK--SAG--SVLYVC--FGSAS-FFTT---RQL---------REIAVGL--
MtUGT73K1     267 L-------DTK--EPS--SVLYIS--FGSLC-RLSN---EQL---------KEMANGI--
GeUGT73F1     265 L-------DSK--RDD--SVLYIC--FGSLC-HFSD---KQL---------YEIACGV--
AtUGT73B1     282 L-------DSK--KCD--SVIYMA--FGTMS-SFKN---EQL---------IEIAAGL--
GuUGAT        272 L-------QSK--EPN--SVVYVC--FGSMM-VFSD---AQL---------KEIAMGL--
NmUGT73BD1    265 L-------DSK--KPY--SVIYAC--FGSVG-RLSK---LQL---------HEIALGL--
NmUGT73BD1      1 --------------------------------------MTAQFHVVFFP-LM-AQGHLIP
```

```
NtTOGT1      272 L-------DSK--KPS--SVVYIC--FGSVA-NFTA---SQL---------HELAMGV--
BvUGT73A4    278 L-------NSK--KPN--SVIYIC--FGSTM-HMIP---SQL---------NEIAMGL--
Db5-GT       279 L-------NSK--KPD--SVIYIC--FGSTG-HLIA---PQL---------HEIATAL--
AtUGT86A1    279 L-------KGR--PTG--SVLYVS--FGSYA-HVGK---KEI---------VEIAHGL--
ZmUFGT1      282 L-------GRQ--PAR--GVAYVS--FGTVA-CPRP---DEL---------RELAAGL--
CteUGT78K6   258 L-------DSK--SSR--SVAYVC--FGTVV-SPPP---QEV---------VAVAEAL--
AtUGT78D1    265 M-------GKR--SAA--SVAYIS--FGTVM-EPPP---EEL---------VAIAQGL--
CoUGT78B3    265 L-------DAQP-KPS--SVAYIS--FGTMA-TPPP---QEL---------KALAEGI--
MtUGT78G1    267 L-------DQH--ENS--SVVYIS--FGSVV-TPPP---HEL---------TALAESL--
LgUGT78J1    256 L-------GKQT-RPK--SVVYIS--FSTVA-TPPE---KEL---------VALAEAL--
CpPGT11      263 L-------DQQ--QAN--SVIYVA--LGSHT-VLEQ---NQF---------QELALGL--
AtUGT83A1    280 L-------DRQ--IPG--SVIYVA--FGSFG-VMGN---PQL---------EELAIGL--
FaUGT75T1    260 L-------DTK--ADS--SVVYVS--FGSMV-VLRS---GQM---------EEMLHGL--
AtUGT75C1    262 L-------DSK--LER--SVIYIS--LGTHADDLPE---KHM---------EALTHGV--
AtUGT75B1    252 L-------DSK--TES--SVIYVS--FGTMV-ELSK---KQI---------EELARAL--
AtUGT75D1    272 L-------DTK--ADS--SVLYVS--FGTLA-VLSK---KQL---------VELCKAL--
ZmUGT74A1    272 L-------DTK--PDR--SVAYVS--FGSLA-SLGN---AQK---------EELARGL--
AsUGT74H5    258 L-------DAQ--PPR--SVTYVS--FGSMA-TPGP---TEM---------AEMAEGL--
BdUGT74J7    277 L-------DSH--PPC--SVVYAS--YGTVA-DLDQ---AQL---------EEIGNGL--
SrUGT74G1    268 L-------DDK--PKE--SVVYVA--FGSLV-KHGP---EQV---------EEITRAL--
AtUGT74B1    269 L-------ETK--QAQ--SVAFVS--FGSFG-ILFE---KQL---------AEVAIAL--
RsUGT74R1    259 L-------CER--RAS--SVIYVS--FGSMA-ILKQ---EQI---------EEIAKCL--
LuUGT74S1    268 L-------QSK--PDG--SVVYVS--FGSLA-RLSP---QQT---------EELYFGL--
AtUGT74F1    258 L-------DKR--PEG--SVVYIA--FGSMA-KLSS---EQM---------EEIASAI--
SgUGT74AC1   263 L-------DSK--PSG--SVLYVS--YGSLV-EMGE---EQL---------KELALGI--
AtUGT74C1    266 L-------GNR--PAK--SVVYVA--FGTLV-ALSE---KQM---------KEIAMAI--
AtUGT74D1    264 L-------DSK--PPG--SVIYVS--FGSLA-VLKD---DQM---------IEVAAGL--
AtUGT74E2    264 L-------NSK--EPN--SVVYLS--FGSLV-ILKE---DQM---------LELAAGL--
AtUGT84B1    263 L-------DKQ--ARS--SVVYIS--FGSML-ETLE---NQV---------ETIAKAL--
DgpHBAGT     273 L-------DSK--PVG--SVVYVS--FGSVA-VLEQ---QSM---------EEITRGL--
MtUGT84F1    267 L-------NTK--PKG--SVVYIS--FGTVV-YLPQ---ELV---------YEIAYGL--
GtUF6CGT1    274 L-------DSR--PTS--TVVYVS--VGSVV-YLKQ---EQV---------TEMAYGI--
PgUGT84A23   269 L-------DSK--PAS--SVVYVS--FGSVV-YLKQ---DQW---------DEIAYGL--
PgUGT84A24   268 L-------DSK--PPA--SVVYVS--FGSVV-YLKQ---DQW---------DEIAFGL--
AtUGT76E1    257 L-------NKQ--KIG--SVIYIS--LGSLA-LMET---KDM---------LEMAWGL--
AtUGT76D1    254 L-------EKQ--ETS--SVIYIS--MGSLA-MTQD---IEA---------VEMAMGF--
SrUGT76G1    268 L-------DQQ--PPS--SVLYVS--FGSTS-EVDE---KDF---------LEIARGL--
VvGT15       288 L-------DKH--SPK--SVIYVS--WGSLA-CMDA---KDL---------AEVAWGL--
AtUGT76C1    264 L-------DMR--ETR--SVVYVS--LGSIA-SLNE---SDF---------LEIACGL--
AtUGT76B1    254 L-------DKQ--ATN--SVIYAS--LGSIA-SIDE---SEF---------LEIAWGL--
CsUGT76F1    256 L-------DKQ--APK--SVIYVS--FGSIA-AVSE---AEF---------LEIAWGL--
OsUGT709A4   281 L-------DGQ--ADR--SVVYVS--LGSLT-VISP---EQF---------TEFLSGL--
SrUGT85C2    288 L-------QSK--EPN--SVVYVN--FGSTT-VMSL---EDM---------TEFGWGL--
CoUGT85N1    286 L-------QEK--DPK--SVLYVN--IGSLA-TMTS---QQL---------GEFAWGL--
MeUGT85K4    288 L-------DKR--EPD--SVVYVN--YGCVT-TITN---EQL---------NEFAWGL--
AtUGT85A1    292 L-------DTK--TQN--SVIYIN--FGSIT-VLSV---KQL---------VEFAWGL--
MtUGT85H2    289 L-------ESK--EPG--SVVYVN--FGSIT-VMTP---EQL---------LEFAWGL--
MdUGT71K1    270 L-------DDQ--PQK--SVVFLC--FGSMG-SFGA---EQV---------KEIAVGL--
AtUGT89A2    276 L-------DGS--PNG--SVLYVC--FGSQK-ALTK---DQC---------DALALGL--
AtUGT89C1    234 L-------DSC--PED-NSVVYVG--FGSQI-RLTA---EQT---------AALAAAL--
AtUGT89B1    274 L-------DAR--EDN--HVVYVC--FGSQV-VLTK---EQT---------LALASGL--
NmUGT89P1    267 L-------DTKTNSDN--SVVYVC--FGSRV-ELTT---EQL---------DSLAAAL--
AtUGT92A1    277 L-------DSK--PDH--SVVYVC--FGSMN-SILQ---THM---------LELAMAL--
GbUGT92K1    287 L-------DSH--PAS--SVLYIS--FGSQN-SISV---SQM---------RELSLGL--
GmUGT92G4    280 L-------DSK--DEN--SVLYIS--FGSLH-TINA---SQM---------MALAEGL--
PoUGT90A7    269 L-------DQKLAQGC--SVLYVA--FGSQA-EIST---KQL---------EAISKGL--
AtUGT90A1    272 L-------DQK--REEGRPVLYVA--FGTQA-EISN---KQL---------MELAFGL--
AcUGT73J1    260 L-------DGK--EEG--SVLYVC--FGSLC-HFSG---GQL---------RELALGL--
MtUGT73P1    281 L-------DSK--KYD--SVLYVS--FGSMN-KFPT---PQL---------VEIAHAL--
SrUGT73E1    284 L-------DER--KLG--SVLYVC--LGSLA-RISA---AQA---------IELGLGL--
CpPGT4       274 L-------DSQ--QPS--SVVYVC--LGSIC-NLPS---SQL---------IELGLGL--
AtUGT73C1    277 L-------DSK--EEG--SVLYVC--LGSIC-NLPL---SQL---------KELGLGL--
CpPGT2       274 L-------DSK--APK--SVVYAC--LGSLC-NLIP---SQM---------RELGLGL--
PjGAT        282 L-------DSM--KPK--SVIYAC--LGSQC-RLVP---AQL---------MELGLAL--
CtiUGT73AE1  278 L-------DSR--EPG--SVIYAC--FGSSS-RVIP---QQL---------IELGLGL--
CaUGT73AH1   281 L-------DAE--KNG--SVIYVC--LGSLS-SLSNLSGAQI---------IELALGL--
PzGAT2       279 L-------DNW--ANG--SVIYAC--LGSIS-SLTC---TQL---------MELALGL--
AcUGT73G1    272 L-------GKK--SAG--SVLYVC--FGSAS-FFTT---RQL---------REIAVGL--
MtUGT73K1    267 L-------DTK--EPS--SVLYIS--FGSLC-RLSN---EQL---------KEMANGI--
GeUGT73F1    265 L-------DSK--RDD--SVLYIC--FGSLC-HFSD---KQL---------YEIACGV--
AtUGT73B1    282 L-------DSK--KCD--SVIYMA--FGTMS-SFKN---EQL---------IEIAAGL--
GuUGAT       272 L-------QSK--EPN--SVVYVC--FGSMM-VFSD---AQL---------KEIAMGL--
NmUGT73BD1   265 L-------DSK--KPY--SVIYAC--FGSVG-RLSK---LQL---------HEIALGL--
NmUGT73BD1     1 --------------------------------------MTAQFHVVFFP-LM-AQGHLIP
```

```
AtUGT81A1     376 KNLGEAVGQVLIIC-G---RNKKL-------------QSKLSSLDWKI-----------
AtUGT80A2     418 ----DVVGFCYLDL-ASNYEPPAELVEWLEAGDKPIYIGFGSLPVQEPEKMTEIIVEALQ
AtUGT80B1     381 ----DVVGYCFLNL-GSKYQPREEFLHWIERGSPPVYIGFGSMPLDDPKQTMDIILETLK
OsZOGT3       296 ----RGSKQRFIWV-M---RDADR------GNIFTDTGEGETRHAKLL----SEFSKQT-
OsZOGT1       290 ----KGSKQRFIWV-L---RDADR--------ANIFADSGESRHAELL----SRFTAET-
ZmcisZOG1     294 ----KGSKQRFVWV-L---RDADR--------ADIYAESGESRHAMFL----SEFTRET-
GmUGT79A6     295 ----ELTGLPFILV-L---NFPSN------------LSAKAELERALP----KGYLERV-
AtUGT79B6     283 ----ELTGLPFLVA-V---KPPKG---------------SSTIQEALP----KGFEERV-
GmUGT91H9     301 ----ELSGLRFFWA-L---RNLQK--------------------EDLP----HGFEERT-
ZmUGT91L1     263 ----ELAGTRFLWA-L---KRPSV---------------AHPDEDLLP----PGFEERT-
PoUGT95A1     360 ----EESDQPFIWV-IQPGSGKSGIPRSFLGPAAAHTDDSEEEEGYYP----DGLDVTV-
MtUGT95B4     310 ----ESCEQPFIWV-VQTGKGRPS----PPRLRGEPGLGIPKAEGYFP----HGLDKRV-
PgUGT95B2     341 ----EESSSPFIWV-I---QPGSGRGGPPRTFLGGKPDSDPDKEGYFP----HGLKEKV-
VvUGT95B6     335 ----EASNRPFIWV-I---QPGSG----RPGPPRRPGSDSNEDSGYYP----DGLEEKV-
AtUGT82A1     310 ----EASGRPFLWA-L-----------------------NRVWQEGLP----PGFVHRVT
VpUGT94F1     281 ----ELSNVNFIWI-I---RFPEG-------------KNSMTVENALP----EGFLERV-
AtUGT87A1     280 ----REAGVKFFWV-A---RGGEL---------------------KLK----EALEGSL-
AtUGT72C1     289 ----ELTGHRFVWV-V---RPPAE--DDPSASMFDKTKNETEPLDFLP----NGFLDRT-
AtUGT72D1     293 ----ELSGQRFVWV-L---RRPAS-------YLGAISSDDEQVSASLP----EGFLDRT-
LjUGT72AD1    300 ----ELSGEKFLWV-I---RPPHK-----LEFIGDFGVGDEDPLKCLP----DGFLERT-
AtUGT72B1     276 ----ADSEQRFLWV-I---RSPSG----IANSSYFDSHSQTDPLTFLP----PGFLERT-
VvUGT1        290 ----EMSGQRFLWV-V---KSPHE---TAANASFFSAQTIKDPFDFLP----KGFLDRT-
LjUGT72AH1    290 ----ELSRKKFLWVNV---RAPND--SAT-ASYLSDDNELSDPLQFLP----PGFLERT-
LjUGT72Z2     310 ----ELSGQKFLWV-V---RAPSD-----SANAAYLGVGNEDPLKFLP----SGFIERT-
GmUGT72X4     291 ----ELSGQRFLWV-L---RAPNN-----SASAAYLEASKEDPLQFLP----SGFLERT-
MtUGT72L1     299 ----ELSNQKFLWV-V---RAPSS---SSSNAAYLSAQNDVDALQFLP----SGFLERT-
PgUGT72BD1    299 ----EQSQHRFIWV-V---RPPQE--NSACGAYFEQAKRADGTPEYLP----DGFVTRT-
AtUGT72E1     285 ----EMSQQRFVWV-V---RPPVDGSACSAYLSANSGKIRDGTPDYLP----EGFVSRT-
OsUGT706C1    296 ----ENSGHRFLWA-V---RSNLG---------------EVDLEALFP----EGFLERT-
OsUGT706D1    295 ----ESSGHRFLWV-V---RSPRQ-----DPANLLEHLPEPDLAALLP----EGFLERT-
MtUGT88E1     297 ----EKSEQRFLWI-V---RSDME-------------SEELSLDELLP----EGFLERT-
VpUGT88D8     286 ----ENSGHRFLWS-V---RNPPG-----------INNEDPDLETLLP----EGFLERT-
NmUGT88P1     300 ----ENSGHRFLWV-M---KSPPI----DDKTKRFLPPPEPDFNVLLP----EGFLERT-
AtUGT88A1     296 ----EKSGQRFLWV-V---RNPPE-----------LEKTELDLKSLLP----EGFLERT-
VvGT7         303 ----ERSGQRFLWV-V---RSPPS----KDPSRRFLAPPEPDLNSLLP----EGFLSRT-
MdUGT88F1     307 ----EASGQRFLWV-V---KKPPV----EEKSKQVHGVDDFDLKGVLP----DGFLDRT-
ScUGT5        298 ----ERSGQRFLWA-V---KKPPF----DKNSKEVEELGEFNVMEIMP----EGFLDRT-
OsUGT707A2    311 ----ERSGHRFLWV-L-RGRPPAG----------SPYPTDADADELLP----EGFLERT-
AtUGT71B1     290 ----ERSGHRFLWS-L---RRASP----VGNKSNPPPGEFTNLEEILP----KGFLDRT-
MdUGT71A15    299 ----EHSGHRFLWS-L---RRPPP------QGKRAMPSDYEDLKTVLP----EGFLDRT-
BvUGT71F1     312 ----EKSGHRFLWS-L---RKPPS-------AGTTQPSQDQTFVEALP----EGFVDRT-
Db6-GT        300 ----EQSGYCFLWS-L---RQPPP--------EGKATPSEEAFLDTLP----EGFVERT-
AtUGT71C1     310 ----EIVDCKFIWS-F---RTNPK--------------EYASPYEALP----HGFMDRV-
AtUGT71C4     308 ----ELVGCRFLWS-I---RTSGD--------------VETNPNDVLP----EGFMGRV-
AtUGT71D1     301 ----ELCQYRFLWS-L---RKEEV------------------TKDDLP----EGFLDRV-
FaUGT71W2     296 ----EKSGHRFLWS-V-RQRPPKG--------KTEFPGEYKNYEDFLP----QGFLERT-
MtUGT71G1     303 ----KHSGVRFLWS-----NSAEK--------------------KVFP----EGFLEWM-
MdUGT71K1     302 ----EQSGQRFLWS-L---RMPSP--------KGIVPSDCSNLEEVLP----DGFLERT-
AtUGT89A2     308 ----EKSMTRFVWV-V---KKDPI-----------------------P----DGFEDRV-
AtUGT89C1     267 ----EKSSVRFIWA-V---RDAAK---------KVNSSDNSVEEDVIP----AGFEERV-
AtUGT89B1     306 ----EKSGVHFIWA-V---KEPVE---------------KDSTRGNIL----DGFDDRV-
NmUGT89P1     301 ----EISGVHFILC-V---KLHQE----------------------IS----KEYEDRV-
AtUGT92A1     309 ----ESSEKNFIWV-V---RPPIG----------VEVKSEFDVKGYLP----EGFEERI-
GbUGT92K1     319 ----EASQQAFVWA-L---RPPVG--------------TTELSSDHLP----HGFEERM-
GmUGT92G4     312 ----EESGKSFIWV-I---RPPVG-----------FDINGEFSAEWLP----KGFEESM-
PoUGT90A7     303 ----EESGVNFLWA-VRKYETSAV----------------------------DELQERV-
AtUGT90A1     306 ----EDSKVNFLWV-T---RKDVE--------------------EIIG----EGFNDRI-
AcUGT73J1     292 ----EKCNKNFLWV-V---RKEAE-------------GDDVSEKEWMP----ENYKERV-
MtUGT73P1     313 ----EDSGHDFIWV-V---RKIED---------------AEDGDDGFL----SEFEKRM-
SrUGT73E1     316 ----ESINRPFIWC-V---RNETD-----------------ELKTWFL----DGFEERV-
CpPGT4        306 ----EASNKPFVWV-I---RGVSK---------------LEALEKWLV---QENFEERI-
AtUGT73C1     309 ----EESQRPFIWV-I---RGWEK---------------YNELLEWIS---ESGYKERI-
CpPGT2        306 ----EASNRPFIWV-I---REGET---------------SKELKKWVV---EDGFEERI-
PjGAT         314 ----ESSKHPFIWV-I---KEGER---------------FQELEKWLV---EEEFEERI-
CtiUGT73AE1   310 ----ESSNRPFIWV-I---RAGDR---------------ATEIEEWIT---ETGFEERT-
CaUGT73AH1    316 ----EMSGYPFIWA-L---RPGIK---------------HKEIEKWVI---EEGFEERV-
PzGAT2        311 ----EASERPFVWV-V---RAGGK---------------QKEIEKWIL---EEGFEEST-
AcUGT73G1     304 ----EGSGHAFIWV-V---RDDGD-------------------EQWMP----EGCEERI-
MtUGT73K1     299 ----EASKHQFLWV-V------HG-------------KEGEDEDNWLP----KGFVERM-
GeUGT73F1     297 ----EASGHEFIWV-VPEKKGKED-------------ESEEEKEKWMP----KGFEERK-
AtUGT73B1     314 ----DMSGHDFVWV-V--NRKGSQ----------------VEKEDWLP----EGFEEKT-
GuUGAT        304 ----EASGKPFIWV-V---KKGGA-------------KSEGEKLEWLP----EGFEERM-
NmUGT73BD1    297 ----ESSGHYFIWV-V---KKSEE------------------AEKWLP----EGFEERI-
NmUGT73BD1      1 --------------------------------------MTAQFHVVFFP-LM-AQGHLIP
```

```
NtTOGT1       304 ----EASGQEFIWV-V---RTELD-----------------NEDWLP----EGFEERT-
BvUGT73A4     310 ----EASGKDFIWV-V---RNEDD--------------------------LGEFEQRM-
Db5-GT        311 ----EASGQDFIWA-V---RGDHG--------------QGNSEEWLP----PGYEHRL-
AtUGT86A1     311 ----LLSGISFIWV-L---RPDIV--------------GSNVPDFLP----AGFVDQA-
ZmUFGT1       314 ----EASGAPFLWS-L---REDSW-------------T------LLP----PGFLDRA-
CteUGT78K6    290 ----EESGFPFVWA-L---KESLL-------------------SILP----KGFVERT-
AtUGT78D1     297 ----ESSKVPFVWS-L---KEKNM-------------------VHLP----KGFLDRT-
CoUGT78B3     298 ----EASGVPFLWS-L---KDSVK-------------------LHLP----HGFLERT-
MtUGT78G1     299 ----EECGFPFIWS-F---RGDPK-------------------EKLP----KGFLERT-
LgUGT78J1     289 ----EACQFPFLWS-L---KEQAR-------------------ESLP----DGFLERT-
CpPGT11       295 ----EICNRAFLWV-V---RPDIT---------------NDANDAYP----EGFRERV-
AtUGT83A1     312 ----ELTKRPVLWV---------------------------TGDQQP----IKLG----
FaUGT75T1     292 ----VDSGLPVLWV-I---RKSGN------------EGDQETENLIN----NTLKKEQ-
AtUGT75C1     295 ----LATNRPFLWI-V---REKNP------------------EEKKK----NRFLELI-
AtUGT75B1     284 ----IEGKRPFLWV-I---TDKSN---------RETKTEGEEETEIE--KIAGFRHEL-
AtUGT75D1     304 ----IQSRRPFLWV-I---TDKSY--------RNKEDEQEKEEDCIS--SSEKSFDEI-
ZmUGT74A1     304 ----LAAGKPFLWV-V---RASDE-------------------HQVP----RYLLAEA-
AsUGT74H5     290 ----HSSGKAFLWV-V---RASEA-------------------SKIP----DGFQERV-
BdUGT74J7     309 ----CNSGKRFLWV-V---RSVDE-------------------HKLS----EELRGKC-
SrUGT74G1     300 ----IDSDVNFLWV-I---KHKEE-------------------GKLP----ENLSEVI-
AtUGT74B1     301 ----QESDLNFLWV-I---KEAHI-------------------AKLP----EGFVEST-
RsUGT74R1     291 ----ENLQTRFIWV-V---RETEM-------------------AKLP----SEFVEWN-
LuUGT74S1     300 ----KNSNHYFLWV-V---RESEV-------------------AKLP--KEEYLS---
AtUGT74F1     290 ------SNFSYLWV-V---RASEE-------------------SKLP----PGFLETV-
SgUGT74AC1    295 ----KETGKFFLWV-V---RDTEA-------------------EKLP----PNFVESV-
AtUGT74C1     298 ----SQTGYHFLWS-V---RESER-------------------SKLP----SGFIEEA-
AtUGT74D1     296 ----KQTGHNFLWV-V---RETET-------------------KKLP----SNYIEDI-
AtUGT74E2     296 ----KQSGRFFLWV-V---RETET-------------------HKIP----RNYVEEI-
AtUGT84B1     295 ----KNRGLPFLWV-I---RPKEK---------------AQNVAVLQ----EMVKE---
DgpHBAGT      305 ----LNCGQPFLWV-V---RPPPK-------------ESSKGGGRLP----DWLVQQA-
MtUGT84F1     299 ----LDSQVTFLWA-----KKQHD--------------------DLP----YGFLEET-
GtUF6CGT1     306 ----LNSEVSFLWV-L---RPPSK-------------RIGTEPHVLP----EEFWEKA-
PgUGT84A23    301 ----LNSGVNFLWV-M---KPPHK-------------DSGYTVLTLP----EGFLEKA-
PgUGT84A24    300 ----LNSGLNFLWV-M---KPPHK-------------DSGYQLLTLP----EGFLEKA-
AtUGT76E1     289 ----RNSNQPFLWV-I---RPGSI-------------PGSEWTESLP----EEFSRLV-
AtUGT76D1     286 ----VQSNQPFLWV-I---RPGSI-------------NGQESLDFLP----EQFNQTV-
SrUGT76G1     300 ----VDSKQSFLWV-V---RPGFV-------------KGSTWVEPLP----DGF---L-
VvGT15        320 ----ANSNQPFLWV-V---RPGSV-------------RGSQWIEQLP----ETFMDTV-
AtUGT76C1     296 ----RNTNQSFLWV-V---RPGSV-------------HGRDWIESLP----SGFMESL-
AtUGT76B1     286 ----RNSNQPFLWV-V---RPGLI-------------HGKEWIEILP----KGFIKNL-
CsUGT76F1     288 ----ANCKLPFLWV-V---RPGLT-------------RGSDCLEPLP----SGFMEMV-
OsUGT709A4    313 ----VAAGHPFLWV-L---RPDMV------------------TARLQ---HADLQEAVA
SrUGT85C2     320 ----ANSNHYFLWI-I---RSNLV---------------IGENAVLP----PELEEHI-
CoUGT85N1     318 ----ANSMCPFLWV-I---RPDIL---------------DRASGIVS----EDYKKEI-
MeUGT85K4     320 ----ANSKHPFLWI-V---RPDVV---------------MGESAVLP----EEFYEEI-
AtUGT85A1     324 ----AGSGKEFLWV-I---RPDLV---------------AGEEAMVP----PDFLMET-
MtUGT85H2     321 ----ANCKKSFLWI-I---RPDLV---------------IGGSVIFS----SEFTNEI-
MdUGT71K1     302 ----EQSGQRFLWS-L---RMPSP-------KGIVPSDCSNLEEVLP----DGFLERT-
AtUGT89A2     308 ----EKSMTRFVWV-V---KKDPI----------------------P----DGFEDRV-
AtUGT89C1     267 ----EKSSVRFIWA-V---RDAAK--------KVNSSDNSVEEDVIP----AGFEERV-
AtUGT89B1     306 ----EKSGVHFIWA-V---KEPVE--------------KDSTRGNIL----DGFDDRV-
NmUGT89P1     301 ----EISGVHFILC-V---KLHQE---------------------IS----KEYEDRV-
AtUGT92A1     309 ----ESSEKNFIWV-V---RPPIG---------VEVKSEFDVKGYLP----EGFEERI-
GbUGT92K1     319 ----EASQQAFVWA-L---RPPVG-------------TTELSSDHLP----HGFEERM-
GmUGT92G4     312 ----EESGKSFIWV-I---RPPVG----------FDINGEFSAEWLP----KGFEESM-
PoUGT90A7     303 ----EESGVNFLWA-VRKYETSAV---------------------------DELQERV-
AtUGT90A1     306 ----EDSKVNFLWV-T---RKDVE-------------------EIIG----EGFNDRI-
AcUGT73J1     292 ----EKCNKNFLWV-V---RKEAE------------GDDVSEKEWMP----ENYKERV-
MtUGT73P1     313 ----EDSGHDFIWV-V---RKIED--------------AEDGDDGFL----SEFEKRM-
SrUGT73E1     316 ----ESINRPFIWC-V---RNETD----------------ELKTWFL----DGFEERV-
CpPGT4        306 ----EASNKPFVWV-I---RGVSK--------------LEALEKWLV---QENFEERI-
AtUGT73C1     309 ----EESQRPFIWV-I---RGWEK--------------YNELLEWIS---ESGYKERI-
CpPGT2        306 ----EASNRPFIWV-I---REGET--------------SKELKKWVV---EDGFEERI-
PjGAT         314 ----ESSKHPFIWV-I---KEGER--------------FQELEKWLV---EEEFEERI-
CtiUGT73AE1   310 ----ESSNRPFIWV-I---RAGDR--------------ATEIEEWIT---ETGFEERT-
CaUGT73AH1    316 ----EMSGYPFIWA-L---RPGIK--------------HKEIEKWVI---EEGFEERV-
PzGAT2        311 ----EASERPFVWV-V---RAGGK--------------QKEIEKWIL---EEGFEEST-
AcUGT73G1     304 ----EGSGHAFIWV-V---RDDGD------------------EQWMP----EGCEERI-
MtUGT73K1     299 ----EASKHQFLWV-V------HG------------KEGEDEDNWLP----KGFVERM-
GeUGT73F1     297 ----EASGHEFIWV-VPEKKGKED------------ESEEEKEKWMP----KGFEERK-
AtUGT73B1     314 ----DMSGHDFVWV-V--NRKGSQ---------------VEKEDWLP----EGFEEKT-
GuUGAT        304 ----EASGKPFIWV-V---KKGGA------------KSEGEKLEWLP----EGFEERM-
NmUGT73BD1    297 ----ESSGHYFIWV-V---KKSEE-----------------AEKWLP----EGFEERI-
NmUGT73BD1      1 -------------------------------------MTAQFHVVFFP-LM-AQGHLIP
```

```
AtUGT81A1     407 -------PVQVKGFITKMEE------------------CMGACDCIITKAGPGTIAEAMI
AtUGT80A2     473 RTKQR--GIINKGWGGLGNL-KEPKDFVYLLDNVPHDWLFPRCKAVVHHGGAGTTAAGLK
AtUGT80B1     436 DTEQR--GIVDRGWGGLGNLATEVPENVFLVEDCPHDWLFPQCSAVVHHGGAGTTATGLK
OsZOGT3       337 --EGT--GMVITGWAPQLEI-----------------LAHGATAAFMSHCGWNSTMESMS
OsZOGT1       329 --EGV--GLVITGWAPQLEI-----------------LAHGATAAFMSHCGWNSTMESLS
ZmcisZOG1     333 --EGT--GLVITGWAPQLEI-----------------LAHGATAAFMSHCGWNSTIESLS
GmUGT79A6     330 --KNR--GVVHSGWFQQQLV-----------------LKHSSVGCYVCHGGFSSVIEAMV
AtUGT79B6     315 --KAR--GVVWGGWVQQPLI-----------------LAHPSIGCFVSHCGFGSMWEALV
GmUGT91H9     328 --KER--GIVWKSWAPQIKI-----------------LGHAAIGGCITHCGTNSLVEMLN
ZmUGT91L1     295 --RGR--GSVVTGWVPQTSI-----------------LGHGAVAAFMMHCGWGSTIEALQ
PoUGT95A1     410 --GNR--GLIITGWAPQLLI-----------------LSHPSTGGFLSHCGWNSTAEAIG
MtUGT95B4     356 --GNR--GLIIRGWAPQLLI-----------------LSHTSTGGFLSHCGWNSTIEAIG
PgUGT95B2     388 --GDR--GLIIRGWAPQLLI-----------------LSHPSTGGFLSHCGWNSTVEAIG
VvUGT95B6     378 --GKR--GLIIRGWAPQLLI-----------------LSHPSTGGFLSHCGWNSTVEAIG
AtUGT82A1     338 ITKNQ--GRIVS-WAPQLEV-----------------LRNDSVGCYVTHCGWNSTMEAVA
VpUGT94F1     315 --KGR--GMVIWKFAPQTRI-----------------LAHKSIGGFVSHCGWSSITESVY
AtUGT87A1     306 -------GVVVS-WCDQLRV-----------------LCHAAIGGFWTHCGYNSTLEGIC
AtUGT72C1     334 --KDI--GLVVRTWAPQEEI-----------------LAHKSTGGFVTHCGWNSVLESIV
AtUGT72D1     333 --RGV--GIVVTQWAPQVEI-----------------LSHRSIGGFLSHCGWSSALESLT
LjUGT72AD1    342 --KGQ--GFLVPYWANQIEI-----------------LSTGVIGGFLCHCGWNSTLESIV
AtUGT72B1     319 --KKR--GFVIPFWAPQAQV-----------------LAHPSTGGFLTHCGWNSTLESVV
VvUGT1        334 --QGL--GLVVSSWAPQVQV-----------------LSHGSTGGFLTHCGWNSTLETIV
LjUGT72AH1    335 --KGQ--GFVMSGWAPQSAI-----------------LSHGSIGAFLTHCGWNSILESVV
LjUGT72Z2     352 --KGQ--GLVIPSWAPQTQI-----------------LSHSSIGGFLTHCGWNSVLESIV
GmUGT72X4     333 --KEK--GLVVASWAPQVQV-----------------LGHNSVGGFLSHCGWNSTLESVQ
MtUGT72L1     343 --KEE--GFVITSWAPQIQI-----------------LSHSSVGGFLSHCGWSSTLESVV
PgUGT72BD1    344 ---QK-LGLVVPMWAPQREI-----------------LAHPSIGGFLSHCGWNSTLESLI
AtUGT72E1     332 --HER--GFMVSSWAPQAEI-----------------LAHQAVGGFLTHCGWNSILESVV
OsUGT706C1    328 --QGR--GFVVKNWAPQSAV-----------------LQHGAVGAFVTHCGWNSSLEAIM
OsUGT706D1    337 --ADK--GMVVKSWAPQAKV-----------------LRHAATAAFVTHCGWNSTLEGIT
MtUGT88E1     331 --KEK--GMVVRNWAPQGSI-----------------LRHSSVGGFVTHCGWNSVLEAIC
VpUGT88D8     322 --KER--GFVIKSWAPQKEV-----------------LSHESVGGFVTHCGRSSILEAVS
NmUGT88P1     343 --KER--GVIVKSWAPQLAI-----------------LNHDAIGGFVTHCGWNSVLEAIC
AtUGT88A1     332 --EDK--GMVVKSWAPQVPV-----------------LNHKAVGGFVTHCGWNSILEAVC
VvGT7         346 --KER--GLMVKSWAPQVAV-----------------LNHASVGGFVTHCGWNSVLEAVC
MdUGT88F1     350 --ADR--GMVVKSWAPQVVV-----------------LKKESVGGFVTHCGWNSVLEAVV
ScUGT5        341 --KDR--GMVVESWVPQVKV-----------------LEHPAVGGFVTHCGWNSVLEAVM
OsUGT707A2    350 --KGR--GMVWPTWAPQKDI-----------------LAHAAVGGFVTHGGWNSTLESLW
AtUGT71B1     333 --VEI--GKIIS-WAPQVDV-----------------LNSPAIGAFVTHCGWNSILESLW
MdUGT71A15    340 --ATV--GKVIG-WAPQAAI-----------------LGHPATGGFVSHCGWNSTLESLW
BvUGT71F1     352 ---AK-IGKIIS-WAPQVSI-----------------LSHPSVGGFVSHCGWNSTLESMW
Db6-GT        339 --SHK--GKIIG-WAPQVSI-----------------LAHKAVGGFVSHCGWNSTLESLW
AtUGT71C1     343 --MDQ--GIVCG-WAPQVEI-----------------LAHKAVGGFVSHCGWNSILESLG
AtUGT71C4     341 --AGR--GLVCG-WAPQVEV-----------------LAHKAIGGFVSHCGWNSTLESLW
AtUGT71D1     330 --DGR--GMICG-WSPQVEI-----------------LAHKAVGGFVSHCGWNSIVESLW
FaUGT71W2     337 --KGV--GMLCG-WAPQVEV-----------------LGHKSTGGFVSHCGWNSILESLW
MtUGT71G1     329 ELEGK--GMICG-WAPQVEV-----------------LAHKAIGGFVSHCGWNSILESMW
MdUGT71K1     341 --NGK--KGLICGWAPQVEI-----------------LAHSATGGFLSHCGWNSILESLW
AtUGT89A2     332 --SGR--GLVVRGWVSQLAV-----------------LRHVAVGGFLSHCGWNSVLEGIT
AtUGT89C1     305 --KEK--GLVIRGWAPQTMI-----------------LEHRAVGSYLTHLGWGSVLEGMV
AtUGT89B1     338 --AGR--GLVIRGWAPQVAV-----------------LRHRAVGAFLTHCGWNSVVEAVV
NmUGT89P1     326 --AGR--GLIIRGWAPQVAI-----------------LRHRAVGAFLTHCGWNSILEGIA
AtUGT92A1     346 TRSER--GLLVKKWAPQVDI-----------------LSHKATCVFLSHCGWNSILESLS
GbUGT92K1     352 RANNK--GFLIRGWAPQLMI-----------------LSHPSTGGFLSHCGWNSVLESIS
GmUGT92G4     348 RDTKR--GLLVHKWGPQLEI-----------------LSHTSTGAFLSHCGWNSVLESLS
PoUGT90A7     329 --GER--GLIVTEWVDQMEI-----------------LKHESVKGFVSHCGWNSVLESIC
AtUGT90A1     333 --RES--GMIVRDWVDQWEI-----------------LSHESVKGFLSHCGWNSAQESIC
AcUGT73J1     326 --GER--GLVVKGWVPQTTV-----------------LDHKSVGWFVTHCGWNSLQESTC
MtUGT73P1     345 --KERNKGYLIWGWAPQLLI-----------------LEHGAVGAVVTHCGWNTIMESVN
SrUGT73E1     346 --RDR--GLIVHGWAPQVLI-----------------LSHPTIGGFLTHCGWNSTIESIT
CpPGT4        339 --KGR--GLLIRGWAPQVLI-----------------LSHPAVGGFLTHCGWNSSLEGIS
AtUGT73C1     342 --KER--GLLITGWSPQMLI-----------------LTHPAVGGFLTHCGWNSTLEGIT
CpPGT2        339 --KGR--GLVIWGWAPQVLI-----------------LSHPSIGGFSTHCGWNSTLEGVS
PjGAT         347 --KRR--GLLIKGWAPQVLI-----------------LSHPAIKAFLTHCGWNSTIEGVC
CtiUGT73AE1   343 --KDR--GLLIRDWAPQLLI-----------------LSHPSVGGFLTHCGWNSILEGVC
CaUGT73AH1    349 --KGR--GMLIWGWAPQVLI-----------------LSHRAIGGFLTHCGWNSTIEGIC
PzGAT2        344 --KGR--GLLIRGWAPQVLI-----------------LSHPAIGGFVTHCGWNSTIEGIC
AcUGT73G1     332 --EGR--GLIIKGWAPQMMI-----------------LNHEAVGGYLTHCGWNSSLEGIC
MtUGT73K1     330 KEEKK--GMLIKGWVPQALI-----------------LDHPSIGGFLTHCGWNATVEAIS
GeUGT73F1     334 ----K--GLIMRGWAPQVLI-----------------LSHRAVGAFVTHCGWNSTVEAVS
AtUGT73B1     346 --KGK--GLIIRGWAPQVLI-----------------LEHKAIGGFLTHCGWNSLLEGVA
GuUGAT        338 GESNK--GLIIRGWAPQVMI-----------------LDHGAVGGFVTHCGWNSTLEGVC
NmUGT73BD1    326 --KDK--GLIIRGWAPQVSI-----------------LEQESTGGFVTHCGWNSTLEAIS
NmUGT73BD1      1 --------------------------------------MTAQFHVVFFP-LM-AQGHLIP
```

```
NtTOGT1       333 --KEK--GLIIRGWAPQVLI-----------------LDHESVGAFVTHCGWNSTLEGVS
BvUGT73A4     334 --EGK--GLIIRGWAPQVLI-----------------LEHEVIGAFVTHCGWNSTIEGIA
Db5-GT        343 --QGK--GLIIRGWAPQVLI-----------------LEHEATGGFLTHCGWNSALEGIS
AtUGT86A1     343 --QDR--GLVVQ-WCCQMEV-----------------ISNPAVGGFFTHCGWNSILESVW
ZmUFGT1       341 AGTGS--GLVVP-WAPQVAV-----------------LRHPSVGAFVTHAGWASVLEGVS
CteUGT78K6    317 --STR--GKVVS-WVPQSHV-----------------LSHGSVGVFVTHCGANSVMESVS
AtUGT78D1     324 --REQ--GIVVP-WAPQVEL-----------------LKHEAMGVNVTHCGWNSVLESVS
CoUGT78B3     325 --SER--GKVVP-WTPQSRL-----------------LRHPAVGVIVTHCGWNSIMESIM
MtUGT78G1     326 --KTK--GKIVA-WAPQVEI-----------------LKHSSVGVFLTHSGWNSVLECIV
LgUGT78J1     316 --TSF--GKIVS-WAPQLQV-----------------LAHDSVGVFVSHCGWNSIIESIS
CpPGT11       326 --AAR--GQMIS-WSPQQKF-----------------LTHPSISCFMSHCGWNSTTEGVS
AtUGT83A1     332 --SDR--VKVVR-WAPQREV-----------------LSSGAIGCFVSHCGWNSTLEGAQ
FaUGT75T1     326 -------GLIVP-WCSQVEV-----------------LSHKSVGCCVSHCGWNSTIESLA
AtUGT75C1     323 RGSDR--GLVVG-WCSQTAV-----------------LAHCAVGCFVTHCGWNSTLESLE
AtUGT75B1     323 --EEV--GMIVS-WCSQIEV-----------------LSHRAVGCFVTHCGWSSTLESLV
AtUGT75D1     344 -------GMVVS-WCDQFRV-----------------LNHRSIGCFVTHCGWNSTLESLV
ZmUGT74A1     331 --TATGAAMVVP-WCPQLDV-----------------LAHPAVGCFVTHCGWNSTLEALS
AsUGT74H5     317 --GGR--GLVVT-WVAQLEV-----------------LAHGAIGCFVTHCGWNSTMEALG
BdUGT74J7     336 --NEM--GLIVS-WCPQLEV-----------------LSHKATGCFLTHCGWNSTTEAIV
SrUGT74G1     327 -KTGK--GLIVA-WCKQLDV-----------------LAHESVGCFVTHCGFNSTLEAIS
AtUGT74B1     328 --KDR--ALLVS-WCNQLEV-----------------LAHESIGCFLTHCGWNSTLEGLS
RsUGT74R1     318 LSSGL--GLVVT-WCNQLDI-----------------LAHETVGCFVTHCGWNSVLEALC
LuUGT74S1     326 --GEK--GLVVS-WCSQLQV-----------------LASGKVGCFVTHCGWNSTLEALS
AtUGT74F1     315 -DKDK--SLVLK-WSPQLQV-----------------LSNKAIGCFMTHCGWNSTMEGLS
SgUGT74AC1    322 --AEK--GLVVS-WCSQLEV-----------------LAHPSVGCFFTHCGWNSTLEALC
AtUGT74C1     325 --EEKDSGLVAK-WVPQLEV-----------------LAHESIGCFVSHCGWNSTLEALC
AtUGT74D1     323 --CDK--GLIVN-WSPQLQV-----------------LAHKSIGCFMTHCGWNSTLEALS
AtUGT74E2     323 --GEK--GLIVS-WSPQLDV-----------------LAHKSIGCFLTHCGWNSTLEGLS
AtUGT84B1     324 ---GQ--GVVLE-WSPQEKI-----------------LSHEAISCFVTHCGWNSTMETVV
DgpHBAGT      338 --DGK--GLVVQ-WCPQERV-----------------LAHPSVACFITHCGWNSSMEILS
MtUGT84F1     324 --SGR--GKVVN-WSPQEQV-----------------LAHPSVACFITHCGWNSSMEALT
GtUF6CGT1     339 --GDR--GKVVQ-WSPQEQV-----------------LAHPATVGFLTHCGWNSTQEAIS
PgUGT84A23    334 --GDR--GKVVQ-WSPQEQV-----------------LAHPATACFVTHCGWNSSMEALT
PgUGT84A24    333 --GDK--GKVVQ-WSPQEQV-----------------LAHPSVACFVTHCGWNSSMEALS
AtUGT76E1     322 --SER--GYIVK-WAPQIEV-----------------LRHPAVGGFWSHCGWNSTLESIG
AtUGT76D1     319 -TDGR--GFVVK-WAPQKEV-----------------LRHRAVGGFWNHGGWNSCLESIS
SrUGT76G1     330 --GER--GRIVK-WVPQQEV-----------------LAHGAIGAFWTHSGWNSTLESVC
VvGT15        353 --GER--CHIVK-WAPQKEV-----------------LGHRAVGGFWSHCGWNSTLESIS
AtUGT76C1     329 --DGK--GKIVR-WAPQLDV-----------------LAHRATGGFLTHNGWNSTLESIC
AtUGT76B1     319 --EGR--CKIVK-WAPQPEV-----------------LAHRATGGFLTHCGWNSTLEGIC
CsUGT76F1     321 --DGR--GHLVK-WAPQQEV-----------------LAHPAVGAFWTHNGWNSTLESIC
OsUGT709A4    343 AAAGHSKARVVR-WAPQRDV-----------------LRHRAVGCFLTHAGWNSTLEAAV
SrUGT85C2     351 --KKR--GFIAS-WCSQEKV-----------------LKHPSVGGFLTHCGWGSTIESLS
CoUGT85N1     349 --GGR--GLLVS-WCQQEKV-----------------LKHPSIGGFLTHCGWNSTLESLC
MeUGT85K4     351 --KDR--GLLVS-WVPQDRV-----------------LQHPAVGVFLSHCGWNSTIECIS
AtUGT85A1     355 --KDR--SMLAS-WCPQEKV-----------------LSHPAIGGFLTHCGWNSILESLS
MtUGT85H2     352 --ADR--GLIAS-WCPQDKV-----------------LNHPSIGGFLTHCGWNSTTESIC
MdUGT71K1     341 --NGK--KGLICGWAPQVEI-----------------LAHSATGGFLSHCGWNSILESLW
AtUGT89A2     332 --SGR--GLVVRGWVSQLAV-----------------LRHVAVGGFLSHCGWNSVLEGIT
AtUGT89C1     305 --KEK--GLVIRGWAPQTMI-----------------LEHRAVGSYLTHLGWGSVLEGMV
AtUGT89B1     338 --AGR--GLVIRGWAPQVAV-----------------LRHRAVGAFLTHCGWNSVVEAVV
NmUGT89P1     326 --AGR--GLIIRGWAPQVAI-----------------LRHRAVGAFLTHCGWNSILEGIA
AtUGT92A1     346 TRSER--GLLVKKWAPQVDI-----------------LSHKATCVFLSHCGWNSILESLS
GbUGT92K1     352 RANNK--GFLIRGWAPQLMI-----------------LSHPSTGGFLSHCGWNSVLESIS
GmUGT92G4     348 RDTKR--GLLVHKWGPQLEI-----------------LSHTSTGAFLSHCGWNSVLESLS
PoUGT90A7     329 --GER--GLIVTEWVDQMEI-----------------LKHESVKGFVSHCGWNSVLESIC
AtUGT90A1     333 --RES--GMIVRDWVDQWEI-----------------LSHESVKGFLSHCGWNSAQESIC
AcUGT73J1     326 --GER--GLVVKGWVPQTTV-----------------LDHKSVGWFVTHCGWNSLQESTC
MtUGT73P1     345 --KERNKGYLIWGWAPQLLI-----------------LEHGAVGAVVTHCGWNTIMESVN
SrUGT73E1     346 --RDR--GLIVHGWAPQVLI-----------------LSHPTIGGFLTHCGWNSTIESIT
CpPGT4        339 --KGR--GLLIRGWAPQVLI-----------------LSHPAVGGFLTHCGWNSSLEGIS
AtUGT73C1     342 --KER--GLLITGWSPQMLI-----------------LTHPAVGGFLTHCGWNSTLEGIT
CpPGT2        339 --KGR--GLVIWGWAPQVLI-----------------LSHPSIGGFSTHCGWNSTLEGVS
PjGAT         347 --KRR--GLLIKGWAPQVLI-----------------LSHPAIKAFLTHCGWNSTIEGVC
CtiUGT73AE1   343 --KDR--GLLIRDWAPQLLI-----------------LSHPSVGGFLTHCGWNSILEGVC
CaUGT73AH1    349 --KGR--GMLIWGWAPQVLI-----------------LSHRAIGGFLTHCGWNSTIEGIC
PzGAT2        344 --KGR--GLLIRGWAPQVLI-----------------LSHPAIGGFVTHCGWNSTIEGIC
AcUGT73G1     332 --EGR--GLIIKGWAPQMMI-----------------LNHEAVGGYLTHCGWNSSLEGIC
MtUGT73K1     330 KEEKK--GMLIKGWVPQALI-----------------LDHPSIGGFLTHCGWNATVEAIS
GeUGT73F1     334 ----K--GLIMRGWAPQVLI-----------------LSHRAVGAFVTHCGWNSTVEAVS
AtUGT73B1     346 --KGK--GLIIRGWAPQVLI-----------------LEHKAIGGFLTHCGWNSLLEGVA
GuUGAT        338 GESNK--GLIIRGWAPQVMI-----------------LDHGAVGGFVTHCGWNSTLEGVC
NmUGT73BD1    326 --KDK--GLIIRGWAPQVSI-----------------LEQESTGGFVTHCGWNSTLEAIS
NmUGT73BD1      1 --------------------------------------MTAQFHVVFFP-LM-AQGHLIP
```

```
AtUGT81A1     442 RGLPIILNGYIAGQ-EAGNVPYVVENGCG-KFS----------------------KSPKE
AtUGT80A2     530 ASCPTTIVPFFGDQPFWGERVHARGVGPSPIPVDEFSLHKL--------EDAINFMLDDK
AtUGT80B1     494 AGCPTTIVPFFGDQFFWGDRIYEKGLGPAPIPIAQLSVENL--------SSSIRFMLQPE
OsZOGT3       376 HGKPILAWPMHSDQ-PWDAELVCKYFKAG-LLV-RPWEKHG------------EVLPAAT
OsZOGT1       368 YGKPILAWPMHSDQ-PWDAELVCKYLKAG-LLV-RPWEKHS------------EVVPAEA
ZmcisZOG1     372 HGKPVLAWPMHSDQ-PWDSELLCKYFKAG-LLV-RPWEKHA------------EIVPAQA
GmUGT79A6     369 NECQLVLLPFKGDQ-FFNSKLIANDLKAG-VEV-NRSDEDG-------------FFHKED
AtUGT79B6     354 NDCQIVFIPHLGEQ-ILNTRLMSEELKVS-VEV-KREETGW--------------FSKES
GmUGT91H9     367 FGHVLVTLPYLLDQ-ALFSRVLEEK-KVG-IEVPRSEKDGS--------------FTRDD
ZmUGT91L1     334 YGHPLVMMPVLVDH-LSTARVMEQR-KVG-VKVRKEKSDEA--------------FLGDN
PoUGT95A1     449 RGVPILGWPIRGDQ-FDNAKLVAYHLKIG--HVMSRGANGE---------VGPGKFTKDD
MtUGT95B4     395 RGIPLLAWPIRGDQ-YHNAKLVVSRLRVG-YMV-SNDLSEK--------------VAKDE
PgUGT95B2     427 RGVPFLTWPIRGDQ-YHNAKLVVAHLRMGHVIL---DDMSL-------------PMKKDD
VvUGT95B6     417 RGVPFLAWPIRGDQ-YSDAMLVVKHLKIG-YMV-FAKDASE-------------NIVKEA
AtUGT82A1     378 SSRRLLCYPVAGDQ-FVNCKYIVDVWKIG-VRL--------------------SGFGEKE
VpUGT94F1     354 FGVPIIAMPMKFEQ-VVNGVVVVEV-GVG-VEV-EKDGSGQ--------------YLGEE
AtUGT87A1     341 SGVPLLTFPVFWDQ-FLNAKMIVEEWRVG-MGI-ERKKQME------------LLIVSDE
AtUGT72C1     373 NGVPMVAWPLYSEQ-KMNARMVSGELKIA-LQI--NVADGI--------------VKKEV
AtUGT72D1     372 KGVPIIAWPLYAEQ-WMNATLLTEEIGVA-VRT-SELPSER-------------VIGREE
LjUGT72AD1    381 HGIPIIAWPLFAEQ-KMNAAMLSDGLKVA-LRP-KRNEKGI--------------VGREV
AtUGT72B1     358 SGIPLIAWPLYAEQ-KMNAVLLSEDIRAA-LRP-RAGDDGL--------------VRREE
VvUGT1        373 QGVPIIAWPLFAEQ-RMNATLLANDLKAA-VTL--NNNNGL--------------VSREE
LjUGT72AH1    374 HGVPMIAWPLFAEQ-RLNAALVTDGLKIA-LRV-KIGGNGV--------------VVKEE
LjUGT72Z2     391 LGVPMITWPLFAEQ-RMNAVLLTDGVKVA-LRP-KFDENGI--------------VKREE
GmUGT72X4     372 EGVPLITWPLFAEQ-RMNAVMLTDGLKVA-LRP-KFNEDGI--------------VEKEE
MtUGT72L1     382 HGVPLITWPMFAEQ-GMNAVLVTEGLKVG-LRP-RVNENGI--------------VERVE
PgUGT72BD1    383 NGVPLIAWPLYAEQ-TMNATMLTDQLGIA-ARS-RELMPNG-------------VLGRHE
AtUGT72E1     371 GGVPMIAWPLFAEQ-MMNATLLNEELGVA-VRS-KKLPSEG-------------VITRAE
OsUGT706C1    367 SGVPMICWPLYAEQ-RLNKAHLVEEMKLG-VLV--EGYDGE-------------LVKADE
OsUGT706D1    376 AGVPLLCWPLYAEQ-RMNKVFIVEEMKVG-VVI--DGYDEE-------------MVSAEE
MtUGT88E1     370 EGVPMITWPLYAEQ-KMNRLILVQEWKVA-LEL-NESKDGF--------------VSENE
VpUGT88D8     361 FGVPMIGFPIYAEQ-RMNRVFMVEEMKVS-LPL---DEAGD------------GLVTSGE
NmUGT88P1     382 GGVPMLAWPLYAEQ-RVNRVCMVEEMKVA-LPL-EESVDGF--------------VMASE
AtUGT88A1     371 AGVPMVAWPLYAEQ-RFNRVMIVDEIKIA-ISM-NESETGF--------------VSSTE
VvGT7         385 AGVPMVAWPLYAEQ-RFNRVVLVEEMKLA-FPM-EESEEGF--------------VTATE
MdUGT88F1     389 AGVPMIAWPLYAEQ-HMNRNVLVTDMEIA-IGVEQRDEEGG-------------FVSGEE
ScUGT5        380 AGVPMVAWPLYAEQ-HLNKAALVEDMKMA-IPM-DPREDDE-------------FMFAEE
OsUGT707A2    389 HGVPMAPWPLYAEQ-HLNAFELVRDMGVA-VEMEVDRKRGN-------------LVEAAE
AtUGT71B1     371 FGVPMAAWPIYAEQ-QFNAFHMVDELGLA-AEVKKEYRRDF-------LVEEPEIVTADE
MdUGT71A15    378 NGVPIAAWPLYAEQ-NLNAFQLVVELGLA-VEI-KMDYRRD----------SDVVVSAED
BvUGT71F1     390 FGVPVATWPLHAEQ-QLNAFELIKELGLA-VEIRMDYRHDW-----KTRKANFVVTAEE
Db6-GT        377 FGVPMATWPISAEQ-QLNAFELVKEFGMA-VEI-RMDFWRD----CRKNTQSFVVTSEE
AtUGT71C1     381 FGVPIATWPMYAEQ-QLNAFTMVKELGLA-LEMRLDYVSED----------GDIVKADE
AtUGT71C4     379 FGVPVATWPMYAEQ-QLNAFTLVKELGLA-VDL-RMDYVSS----------RGGLVTCDE
AtUGT71D1     368 FGVPIVTWPMYAEQ-QLNAFLMVKELKLA-VELKLDYRVHS----------DEIVNANE
FaUGT71W2     375 YGVPIVTWPLYAEQ-QVNAFLIARDLGLG-VELRLDYVVGS----------GDFVSADE
MtUGT71G1     369 FGVPILTWPIYAEQ-QLNAFRLVKEWGVG-LGL-RVDYRKG----------SDVVAAEE
MdUGT71K1     380 HGVPIATWPMYAEQ-QLNAFRMVRELGMA-LEM-RLDYKAG---------SADVVGADE
AtUGT89A2     371 SGAVILGWPMEADQ-FVNARLLVEHLGVA-VRV-CEGGETV--------------PDSDE
AtUGT89C1     344 GGVMLLAWPMQADH-FFNTTLIVDKLRAA-VRV---GENRD-------------SVPDSDK
AtUGT89B1     377 AGVLMLTWPMRADQ-YTDASLVVDELKVG-VRA-CEGPDTV--------------PDPDE
NmUGT89P1     365 AGVVMLTWPMGADQ-FTNANLLVDELKVA-MKA-CEGGDSN-------------VPNPAM
AtUGT92A1     387 HGVPLLGWPMAAEQ-FFNSILMEKHIGVS-VEV-ARGKRCE--------------IKCDD
GbUGT92K1     393 QGVPIIGWPIAAEQ-FYNSKLLEEEVGVV-VEL-CRGIDGE--------------VRKNN
GmUGT92G4     389 YGVPMIGWPIVADQ-PYNVKMLVEEMGVA-VEL-TRSTETV--------------VSREK
PoUGT90A7     368 SEVPILAWPMMAEQ-PLNTRMVVEELKIG-LRVETCDGSVK------------GFVKSEG
AtUGT90A1     372 VGVPLLAWPMMAEQ-PLNAKMVVEEIKVG-VRV-ETEDGSV-----------KGFVTREE
AcUGT73J1     365 AGVPMITWPLFHEQ-FINAEFLVETMGIG-ERMWEGFRKSE------YRKFDDVIVTADE
MtUGT73P1     386 AGLPLATWPLFAEQ-FFNERLLVDVLKIG-VAV---GAKEWRNWNEFG----DDVVKRED
SrUGT73E1     385 AGVPMITWPFFADQ-FLNEAFIVEVLKIG-VRI---GVERA--CLFGEEDKVGVLVKKED
CpPGT4        378 AGVQMLTWPLFADQ-FCNEKLIVNVLRIG-VSVGVEVPMNF-----GEEEKIGVLVNKEN
AtUGT73C1     381 SGVPLLTWPLFGDQ-FCNEKLAVQILKAG-VRAGVEESMRW-----GEEEKIGVLVDKEG
CpPGT2        378 AGLPLLTWPLFGDQ-FMNEVFVVQILKIG-VKVGVESPMIW-----GEEQKIGVLMKRDD
PjGAT         386 SGVPMITWPMFAEQ-FFNEKLIVDILRIG-IKVGVQVSVRW-----GEEEKIGVLVKREQ
CtiUGT73AE1   382 AGVPLVTWPLFAEQ-FLNEKLVVEVLGVG-VPVGAPRVVHW-----GMEDKFGVTVRSDQ
CaUGT73AH1    388 AGVPMITWPLFAEQ-FVNEKFLVQVVKTG-VRVGVEAVVDM-----GYKEEFGVKVKRED
PzGAT2        383 SGVPMITWPLFAEQ-FFNEKLVVQVLETG-VSVGAKEVMPL-----GEEEKFGVKVRSKN
AcUGT73G1     371 VGLPFVTWPLFAEQ-PYNERLIVDVLKVG-VAVGVKEYSFD--------PEERTVIEAGS
MtUGT73K1     371 SGVPMVTMPGFGDQ-YYNEKLVTEVHRIG-VEV---GAAEW---SMSPYDAKKTVVRAER
GeUGT73F1     371 AGVPMITWPVHGEQ-FYNEKLVTQVRGIG-VEV---GAEEW---SAIGFGEREKVVCRES
AtUGT73B1     385 AGLPMVTWPVGAEQ-FYNEKLVTQVLKTG-VSVGVKKMMQV----------VGDFISREK
GuUGAT        379 AGVPMVTWPMYGEQ-FYNAKFLTDIVKIG-VGV---GVQTW------IGMGGGEPVKKEV
NmUGT73BD1    365 AGVPMATLPTFAEQ-PFNEKLLTRVLKIG-IPVGSPLSNRA-----------VSLVKREE
NmUGT73BD1      1 --------------------------------------MTAQFHVVFFP-LM-AQGHLIP
```

```
NtTOGT1        372 GGVPMVTWPVFAEQ-FFNEKLVTEVLKTG-AGVGSIQWKRS----------ASEGVKREA
BvUGT73A4      373 AGVPMVTWPVFAEQ-FLNEKLITRVLRIG-IPV---GAKKW-----DCKPSEEYVVKKND
Db5-GT         382 AGVPMVTWPTFAEQ-FHNEQLLTQILKVG-VAV---GSKKW-----TLKPSIEDVIKAED
AtUGT86A1      381 CGLPLLCYPLLTDQ-FTNRKLVVDDWCIG-INL---CEKKT---------------ITRDQ
ZmUFGT1        381 SGVPMACRPFFGDQ-RMNARSVAHVWGFG-AAF-----EGA---------------MTSAG
CteUGT78K6     355 NGVPMICRPFFGDQ-GIAARVIQDIWEVG-VIV-----EGK--------------VFTKNG
AtUGT78D1      362 AGVPMIGRPILADN-RLNGRAVEVVWKVG-VMM----DNGV---------------FTKEG
CoUGT78B3      363 GAVPIVYRPFFGDH-MLISRFVSDIWKIG-VSA------GD-------------VVFTKDG
MtUGT78G1      364 GGVPMISRPFFGDQ-GLNTILTESVLEIG-VGV----DNGV---------------LTKES
LgUGT78J1      354 SGVPMICRPFFGDQ-KLNSRMIQDSWKIG-LRI----EGGV---------------FSKSG
CpPGT11        364 NGVPFLCWPFFADQ-FMNTTYICDVWKVG-LRL-ERNQSGI---------------IGREE
AtUGT83A1      370 NGIPFLCIPYFADQ-FINKAYICDVWKIG-LGL-ERDARGV---------------VPRLE
FaUGT75T1      361 AGVPVVGCPHFADQ-TTNAKLVEELWGSG-VRA-RANEEGV---------------IERAE
AtUGT75C1      363 SGVPVVAFPQFADQ-CTTAKLVEDTWRIG-VKV-KVGEEGD---------------VDGEE
AtUGT75B1      361 LGVPVVAFPMWSDQ-PTNAKLLEESWKTG-VRV-RENKDGL---------------VERGE
AtUGT75D1      379 SGVPVVAFPQWNDQ-MTNAKLLEDCWKTG-VRVMEKKEEEG------------VVVVDSEE
ZmUGT74A1      371 FGVPMVAMALWTDQ-PTNARNVELAWGAG-VRARRDAGAGV---------------FLRGE
AsUGT74H5      355 AGVPMVAVPQWSDQ-PTNAKFVEDVWCVG-VRA-RRDPEGV---------------VRREE
BdUGT74J7      374 TGVPLLAMPQWTDQ-PTTAKYVESAWGIG-VRV-HRDNEGV---------------VRKEE
SrUGT74G1      366 LGVPVVAMPQFSDQ-TTNAKLLDEILGVG-VRV-KADENGI---------------VRRGN
AtUGT74B1      366 LGVPMVGVPQWSDQ-MNDAKFVEEVWKVG-YRA-KEEAGEV--------------IVKSEE
RsUGT74R1      358 LGVPMVGVPNWSDQ-PTNAKFVEDVWKVG-VRA-KEDEDGI---------------VKSMV
LuUGT74S1      364 LGVPMVAMPECGDQ-LTNAKFIKDVWKTG-VRAEADDGKGI----------MWGMIKREV
AtUGT74F1      354 LGVPMVAMPQWTDQ-PMNAKYIQDVWKVG-VRVKAEKESGI---------------CKREE
SgUGT74AC1     360 LGVPVVAFPQWADQ-VTNAKFLEDVWKVG-KRV-KRNEQRL---------------ASKEE
AtUGT74C1      365 LGVPMVGVPQWTDQ-PTNAKFIEDVWKIG-VRV-RTDGEGL---------------SSKEE
AtUGT74D1      361 LGVALIGMPAYSDQ-PTNAKFIEDVWKVG-VRV-KADQNGF---------------VPKEE
AtUGT74E2      361 LGVPMIGMPHWTDQ-PTNAKFMQDVWKVG-VRV-KAEGDGF---------------VRREE
AtUGT84B1      361 AGVPVVAYPSWTDQ-PIDARLLVDVFGIG-VRMRNDSVDGE---------------LKVEE
DgpHBAGT       376 SGVPIVAAPQWGDQ-VTNAKFLTDVYGVG-VRL-LRSENDL-------------NTFSSER
MtUGT84F1      362 LGVPMLTFPTFGDQ-LTNAKFLVDVYGVG-IRL-ARGERKL---------------VRRDD
GtUF6CGT1      377 SGVPVITFPQFGDQ-VTNAKFLVEEFKVG-VRL-GRGELEN-------------RIITRDE
PgUGT84A23     372 SGMPVVAFPQWGDQ-VTDAKYLVDEFKVG-VRM-CRGEAED-------------KLITRDV
PgUGT84A24     371 SGMPVVAFPQWGDQ-VTDAKYLVDVFKVG-VRM-CRGEAEN-------------KLIMRDV
AtUGT76E1      360 EGVPMICRPFTGDQ-KVNARYLERVWRIG-VQL-----EGE---------------LDKGT
AtUGT76D1      358 SGVPMICRPYSGDQ-RVNTRLMSHVWQTA-YEI-----EGE---------------LERGA
SrUGT76G1      368 EGVPMIFSDFGLDQ-PLNARYMSDVLKVG-VYL----ENGW----------------ERGE
VvGT15         391 EGVPMICRPYSGDQ-RVNTRYISHVWKVG-LEL----ESDE---------------LERVE
AtUGT76C1      367 EGVPMICLPCKWDQ-FVNARFISEVWRVG-IHL-----EGR---------------IERRE
AtUGT76B1      357 EAIPMICRPSFGDQ-RVNARYINDVWKIG-LHL-----ENK---------------VERLV
CsUGT76F1      359 EGIPMICMPCFTDQ-KVNARYVSDVWKVG-LQL----ENGL----------------KREE
OsUGT709A4     385 EGVPTVCWPFFVDQ-QINSRFVGGVWGTG-LDM------------------KDACDAAV
SrUGT85C2      389 AGVPMICWPYSWDQ-LTNCRYICKEWEVG-LEM------------------GTKVKRDE
CoUGT85N1      387 EGVPMICWPFFAEQ-QTNCFYICNKWGIG-MEI------------------DFDVKRVE
MeUGT85K4      389 GGKPMICWPFFAEQ-QTNCKYACDVWKTG-VEL------------------STNLKREE
AtUGT85A1      393 CGVPMVCWPFFADQ-QMNCKFCCDEWDVG-IEI-----GGD---------------VKREE
MtUGT85H2      390 AGVPMLCWPFFADQ-PTDCRFICNEWEIG-MEI------------------DTNVKREE
MdUGT71K1      380 HGVPIATWPMYAEQ-QLNAFRMVRELGMA-LEM-RLDYKAG----------SADVVGADE
AtUGT89A2      371 SGAVILGWPMEADQ-FVNARLLVEHLGVA-VRV-CEGGETV---------------PDSDE
AtUGT89C1      344 GGVMLLAWPMQADH-FFNTTLIVDKLRAA-VRV---GENRD--------------SVPDSDK
AtUGT89B1      377 AGVLMLTWPMRADQ-YTDASLVVDELKVG-VRA-CEGPDTV---------------PDPDE
NmUGT89P1      365 AGVVMLTWPMGADQ-FTNANLLVDELKVA-MKA-CEGGDSN--------------VPNPAM
AtUGT92A1      387 HGVPLLGWPMAAEQ-FFNSILMEKHIGVS-VEV-ARGKRCE---------------IKCDD
GbUGT92K1      393 QGVPIIGWPIAAEQ-FYNSKLLEEEVGVV-VEL-CRGIDGE---------------VRKNN
GmUGT92G4      389 YGVPMIGWPIVADQ-PYNVKMLVEEMGVA-VEL-TRSTETV---------------VSREK
PoUGT90A7      368 SEVPILAWPMMAEQ-PLNTRMVVEELKIG-LRVETCDGSVK-------------GFVKSEG
AtUGT90A1      372 VGVPLLAWPMMAEQ-PLNAKMVVEEIKVG-VRV-ETEDGSV------------KGFVTREE
AcUGT73J1      365 AGVPMITWPLFHEQ-FINAEFLVETMGIG-ERMWEGFRKSE------YRKFDDVIVTADE
MtUGT73P1      386 AGLPLATWPLFAEQ-FFNERLLVDVLKIG-VAV---GAKEWRNWNEFG----DDVVKRED
SrUGT73E1      385 AGVPMITWPFFADQ-FLNEAFIVEVLKIG-VRI---GVERA--CLFGEEDKVGVLVKKED
CpPGT4         378 AGVQMLTWPLFADQ-FCNEKLIVNVLRIG-VSVGVEVPMNF-----GEEEKIGVLVNKEN
AtUGT73C1      381 SGVPLLTWPLFGDQ-FCNEKLAVQILKAG-VRAGVEESMRW-----GEEEKIGVLVDKEG
CpPGT2         378 AGLPLLTWPLFGDQ-FMNEVFVVQILKIG-VKVGVESPMIW-----GEEQKIGVLMKRDD
PjGAT          386 SGVPMITWPMFAEQ-FFNEKLIVDILRIG-IKVGVQVSVRW-----GEEEKIGVLVKREQ
CtiUGT73AE1    382 AGVPLVTWPLFAEQ-FLNEKLVVEVLGVG-VPVGAPRVVHW-----GMEDKFGVTVRSDQ
CaUGT73AH1     388 AGVPMITWPLFAEQ-FVNEKFLVQVVKTG-VRVGVEAVVDM-----GYKEEFGVKVKRED
PzGAT2         383 SGVPMITWPLFAEQ-FFNEKLVVQVLETG-VSVGAKEVMPL-----GEEEKFGVKVRSKN
AcUGT73G1      371 VGLPFVTWPLFAEQ-PYNERLIVDVLKVG-VAVGVKEYSFD--------PEERTVIEAGS
MtUGT73K1      371 SGVPMVTMPGFGDQ-YYNEKLVTEVHRIG-VEV---GAAEW---SMSPYDAKKTVVRAER
GeUGT73F1      371 AGVPMITWPVHGEQ-FYNEKLVTQVRGIG-VEV---GAEEW---SAIGFGEREKVVCRES
AtUGT73B1      385 AGLPMVTWPVGAEQ-FYNEKLVTQVLKTG-VSVGVKKMMQV----------VGDFISREK
GuUGAT         379 AGVPMVTWPMYGEQ-FYNAKFLTDIVKIG-VGV---GVQTW------IGMGGGEPVKKEV
NmUGT73BD1     365 AGVPMATLPTFAEQ-PFNEKLLTRVLKIG-IPVGSPLSNRA----------VSLVKREE
NmUGT73BD1       1 -----------------------------------------MTAQFHVVFFP-LM-AQGHLIP
```

```
AtUGT81A1     478 ISKIVADWF----GPASK--ELEIMSQN----ALRLAKPEAV------FKIVHDMHELVR
AtUGT80A2     582 VKSSAETLAKAMKDEDGVAGAVKAFFKHLPS-AKQNISDPIP-E----PSGFLSFRKCFG
AtUGT80B1     546 VKSQVMELAKVLENEDGV--AAAVDAFH----RHLPPELPLP-ESSSEKKDEDDRPDLLQ
OsZOGT3       421 IQEVIKKMM---ASDEGL--AVRQRAKA----LGDAVR-----------SSRNDLEDFIA
OsZOGT1       413 IQEVIEEAM---LPEKGM--TIRRRAKE----LGEAVRASVA-D-G--GSSRKGLDDFVG
ZmcisZOG1     417 IQKVIEEAM---LSDSGM--AVRQRAKE----LGEAVRASVA-D-G--GNSRKDLDDFIG
GmUGT79A6     413 ILEALKTVMLEDNKEQGK--QIRENHMQ----WSKFLSN----K----EIQNKFITDLVA
AtUGT79B6     397 LSGAVRSVM-DRDSELGN--WARRNHVK----WKESLLRH--------GLMSGYLNKFVE
GmUGT91H9     410 VAKTLKLAI---VDEEGS--DYRKNAKE----MGKVFSST--------DLHSRYIDDCIV
ZmUGT91L1     377 IATAIRAVM--CEEESKR--IFVANAKR----MQEIVAD----D----ECHKRYIDEFVQ
PoUGT95A1     497 ITSGIEKLM------KDE--KVHKQAKE----LSKEFEGGFP------VSSVKALGAFVE
MtUGT95B4     438 IVMGIERLM----GDE----EMKKTAEV----LSAKFRSGFP------RSSLAALDAFKD
PgUGT95B2     470 IVKGIERLM----GDEGV----KERAAQ----LGKRFEGGFP------ASSAAGLDAFAE
VvUGT95B6     461 IVDGIEKVM------SDK--DMKKRAET----ISGKFGNGFP------ATSAAALDAFRD
AtUGT82A1     416 VEDGLRKVM------EDQ--DMGERLRK----LRDRAMGNEA-R----LSSEMNFTFLKN
VpUGT94F1     396 VAKALDKVF--GDNEFSK--EVRYRASN----LSDKIRENEE------QEEDKVAEQLMS
AtUGT87A1     386 IKELVKRFM-DGESEEGK--EMRRRTCD----LSEICRGAVA-K-G--GSSDANIDAFIK
AtUGT72C1     415 IAEMVKRVM---DEEEGK--EMRKNVKE----LKKTAEEALN-----------MTHIPS
AtUGT72D1     416 VASLVRKIM-AEEDEEGQ--KIRAKAEE----VRVSSERAWS-K-D--GSSYNSLFEWAK
LjUGT72AD1    424 VAEVIKNIL---VGEEGK--GIHQRMKR----LQDVAIDALKED----GSSTRTMTQLAL
AtUGT72B1     401 VARVVKGLM---EGEEGK--GVRNKMKE----LKEAACRVLKDD----GTSTKALSLVAL
VvUGT1        415 IAKTVKSLI---EGEKGK--MIRNKIKD----LKDAATMALSQD----GSSTRSLAEVAQ
LjUGT72AH1    417 IVGLVKGVM---EGLDGG--EIWKRVKE----LQGFAACAMMED----GSSTKTLSKLAI
LjUGT72Z2     434 IAVVVKGLM---QGEEGN--GIHHRIEV----LKEAAADALKED----GSSTRALSQFGN
GmUGT72X4     415 IAKVIKCLM---DGEEGI--GMRERMGN----LKDSAASALK-D----GSSSQTLSQLAS
MtUGT72L1     425 VAKVIKRLM---EGEECE--KLHNNMKE----LKEVASNALKED----GSSTKTISQLTL
PgUGT72BD1    427 IEKMVRTVM-NKEDAKGE--TIRARSQD----LKRTALLAVA-E-G--GSSYAALSQVAQ
AtUGT72E1     415 IEALVRKIM---VEEEGA--EMRKKIKK----LKETAAESLSCD-G--GVAHESLSRIAD
OsUGT706C1    410 LETKVRLVM---ESEEGK--RLRERSAM----AKEMAADAVK-D-G--GSSDMAFAEFLN
OsUGT706D1    419 VEAKVRLVM---ESEEGG--KLLERLAV----ARAKAVEALAEE----GPSRVAFDEFID
MtUGT88E1     413 LGERVKELM---ESEKGK--EVRETILK----MKISAKEARG-G-G--GSSLVDLKKLGD
VpUGT88D8     404 LEKRVKELM---GSVSGK--AIRQRVNE----LKVSGEAAVK-E-G--GSSVVDLDKFIK
NmUGT88P1     425 IEKRVKELV---DYESSE--AIRDQVKI----MSEKAKTAVAVS----GSSHDALTKLLD
AtUGT88A1     414 VEKRVQEII----GEC----PVRERTMA----MKNAAELALTET----GSSHTALTTLLQ
VvGT7         428 VEKRVRELM---ESEEGN--TLRLRIMA----MKEAAETAMS-D-G--GSSRTALTKLVK
MdUGT88F1     434 VERRVRELM---ESEGGR--VLRERCKK----LGEMASAALGET----GSSTRNLVNFVS
ScUGT5        424 VEKRIREVM---DGEKSK--ELREQCHK----MKNMAIGAWERL----GSSTVALDKVVH
OsUGT707A2    434 LERAVRCLM-DEGSEEGR--MAREKAAA----AKAACRNAVD-G-G--GSSIAALRKLTQ
AtUGT71B1     422 IERGIKCAM-----EQDS--KMRKRVME----MKDKLHVALV-D-G--GSSNCALKKFVQ
MdUGT71A15    425 IERGIRRVM-----ELDS--DVRKRVKE----MSEKSKKALV-D-G--GSSYSSLGRFID
BvUGT71F1     442 VENGVQKLM-----SLDE--ETKKRVRQ----MRDEGRKALE-D-G--GSSHMSLARFIQ
Db6-GT        429 IENGVKKLM-----SMDE--EMVEKVKK----MSDKSRKTLE-D-G--GSSHHSLGRFIN
AtUGT71C1     428 IAGTVRSLM---DGVDVP----KSKVKE----IAEAGKEAVD---G--GSSFLAVKRFIG
AtUGT71C4     426 IARAVRSLM-----DGGD--EKRKKVKE----MADAARKALM-D-G--GSSSLATARFIA
AtUGT71D1     415 IETAIRYVM----DTDNN--VVRKRVMD----ISQMIQRATK-N-G--GSSFAAIEKFIY
FaUGT71W2     422 IERAVTGLM---VGDS----EIRKRVVE----MSEMCRRAVD-D-G--GSSSTSLGSLIK
MtUGT71G1     415 IEKGLKDLM-----DKDS--IVHKKVQE----MKEMSRNAVV-D-G--GSSLISVGKLID
MdUGT71K1     427 IEKAVVGVM-----EKDS--EVRKKVEE----MGKMARKAVK-D-G--GSSFASVGRFIE
AtUGT89A2     414 LGRVIAETM----GEGGR--EVAARAEE----IRRKTEAAVT-EAN--GSSVENVQRLVK
AtUGT89C1     387 LARILAESA---REDLPE----RVTLMK----LREKAMEAIK-E-G--GSSYKNLDELVA
AtUGT89B1     420 LARVFADSV---TGNQTE----RIKAVE----LRKAALDAIQER----GSSVNDLDGFIQ
NmUGT89P1     409 LANVLAESI---NGGRAE----RERVTE----LCDAALKAVQ-SGN--GSSAKDLDSLTN
AtUGT92A1     430 IVSKIKLVM--EETEVGK--EIRKKARE----VKELVRRAMV-D-GVKGSSVIGLEEFLD
GbUGT92K1     436 VERIVKMLF-DGHDEKGI--ELRKRAMD----LKAAATIAVSST----GSSITHLDDFIQ
GmUGT92G4     432 VKKTIEIVM-D-YEGKGK--VMKEKANEIAAYIREAKTEKGKEK----GSSVRAMDDLVT
PoUGT90A7     414 LKKMVKELM---EGENGK--EVWKKVKE----VGEAAKVAMA-E-G--GSSWRTLNELID
AtUGT90A1     418 LSGKIKELM---EGETGK--TARKNVKE----YSKMAKAALV-EGT--GSSWKNLDMILK
AcUGT73J1     417 IAGVVGRVM--GGGEKYE--EMKRKAKD----YGEKAKKAVD-E-G--GSSYNDVVALIE
MtUGT73P1     437 IGKAIGLLM-G-GGEECL--EMRKRVKA----LSGAAKKAIE-V-G--GSSYTKLKELIE
SrUGT73E1     438 VKKAVECLM--DEDEDGD--QRRKRVIE----LAKMAKIAMA-E-G--GSSYENVSSLIR
CpPGT4        431 VKTAINILM---DDGEER--NARRSARE----FGELAKRAID-E-G--GSSYNNVELFMQ
AtUGT73C1     434 VKKAVEELM--GDSNDAK--ERRKRVKE----LGELAHKAVE-E-G--GSSHSNITFLLQ
CpPGT2        431 VRNAVEKLM--DEGKEGE--ERRNRAVK----LGQMANMAVQ-E-G--GSSHLNVTLVIQ
PjGAT         439 IQKAIETIM-NGGGEEGG---IRKRATK----LSKVGARAME-D-G--GSSHFNISLLIQ
CtiUGT73AE1   435 VSNAIQKAM--DVGNEGN--ERRRKVKA----LGKVAENAIE-E-G--GSSYKNLTLLIK
CaUGT73AH1    441 VKNAVECIM--SEDEEGE--YRRNRAKK----LAEMAVKAVE-E-G--GSSNLNMTLLIQ
PzGAT2        436 VKEAIECIM--LEGKEGQ--ERRKRARE----LAKEAVRAVE-E-G--GSSHLNITLLIE
AcUGT73G1     421 IETAVKKLM--GDDEEAE--ERRRRAKE----LAAMARKAVE-E-G--GSSYELMSDLIR
MtUGT73K1     423 IEKAVKKLM--DSNGEGG--EIRKRAKE----MKEKAWKAVQ-E-G--GSSQNCLTKLVD
GeUGT73F1     423 IEKAVRRLM--DGGDEAE--KIRRRARE----FRDKATRAVQ-E-G--GSSHNNLTALID
AtUGT73B1     433 VEGAVREVM------VGE--ERRKRAKE----LAEMAKNAVK-E-G--GSSDLEVDRLME
GuUGAT        428 IEQAVRRIM---VGQEAE--EMRNRAKE----LSQMAKRAVE-E-G--GSSHNDFNSLIE
NmUGT73BD1    412 VSDAVKRVM---EGEEAI--EMRTRAKS----LKKMACKAVE-E-G--GSSYNDLTSLID
NmUGT73BD1      1 ---------------------------------------MTAQFHVVFFP-LM-AQGHLIP
```

```
NtTOGT1       420 IAKAIKRVM---VSEEAD--GFRNRAKA----YKEMARKAIE-E-G--GSSYTGLTTLLE
BvUGT73A4     423 IEKALREVM---EGNEAE--ERRTRAKE----YKEMAWKALQ-E-G--GSSYSDLSALID
Db5-GT        432 IEKAVREVM---VGEEGE--ERRRRAKK----LKEMAWRAIE-E-G--GSSYSDLSALIE
AtUGT86A1     422 VSANVKRLM---NGETSS--ELRNNVEK----VKRHLKDAVTTV----GSSETNFNLFVS
ZmUFGT1       420 VAAAVEELL---RGEEGA--RMRARAKV----LQALVAEAFG-P-G--GECRKNFDRFVE
CteUGT78K6    395 FVKSLNLIL---VQEDGK--KIRDNALK----VKQIVQDAVGPH----GQAAEDFNTLVE
AtUGT78D1     402 FEKCLNDVF-V--HDDGK--TMKANAKK----LKEKLQEDFS-M-K--GSSLENFKILLD
CoUGT78B3     403 VVNALDTIL---HKEEGK--RIRESVGM----WKLKATQVVGDS----GSTTHNLDKLLK
MtUGT78G1     404 IKKALELTM---SSEKGG--IMRQKIVK----LKESAFKAVEQN----GTSAMDFTTLIQ
LgUGT78J1     394 AMEALNRIM---TGDEGK--IIRENVNV----LKEKATTAVEPQ----GSSSKNFQKLLQ
CpPGT11       407 IKNKVDQVL----GDQ----NFKARALK----LKEKALSSVR-E-G--GSSNKAIQNFVQ
AtUGT83A1     413 VKKKIDEIM-R----DGG--EYEERAMK----VKEIVMKSVA-K-D--GISCENLNKFVN
FaUGT75T1     404 IKRCLEVVM--GDGVRGE--EMRRNAQK----WKSLAMKAVNES----GSSDDNLRNFVR
AtUGT75C1     406 IRRCLEKVM--SGGEEAE--EMRENAEK----WKAMAVDAAA-E-G--GPSDLNLKGFVD
AtUGT75B1     404 IRRCLEAVM----EEKSV--ELRENAKK----WKRLAMEAGR-E-G--GSSDKNMEAFVE
AtUGT75D1     426 IRRCIEEVM----EDKAE--EFRGNATR----WKDLAAEAVR-E-G--GSSFNHLKAFVD
ZmUGT74A1     415 VERCVRAVM--DGGEAAS--AARKAAGE----WRDRARAAVA-P-G--GSSDRNLDEFVQ
AsUGT74H5     398 LERCIREVT----GDD----KYACNALD----WKEKSKRAMS-Q-G--GSSDMNITEFLQ
BdUGT74J7     417 VERCIREVL---DGERKE--EYRKNAAR----WMKKAKEAMQ-E-G--GSSDKNIAEFAA
SrUGT74G1     409 LASCIKMIM---EEERGV--IIRKNAVK----WKDLAKVAVH-E-G--GSSDNDIVEFVS
AtUGT74B1     410 LVRCLKGVM---EGESSV--KIRESSKK----WKDLAVKAMS-E-G--GSSDRSINEFIE
RsUGT74R1     401 LEKCVRAVL---EGEKGE--VVRRNAGK----IKRWALEAVQLG----GSSDNNIAKFVT
LuUGT74S1     412 IERCIREVM------EGE--ETRRNADK----WGKIIKEAVV-E-G--GSSDKNTEDFAT
AtUGT74F1     398 IEFSIKEVM---EGEKSK--EMKENAGK----WRDLAVKSLS-E-G--GSTDININEFVS
SgUGT74AC1    403 VRSCIWEVM---EGERAS--EFKSNSME----WKKWAKEAVD-E-G--GSSDKNIEEFVA
AtUGT74C1     408 IARCIVEVM---EGERGK--EIRKNVEK----LKVLAREAIS-E-G--GSSDKKIDEFVA
AtUGT74D1     404 IVRCVGEVM-EDMSEKGK--EIRKNARR----LMEFAREALS-D-G--GNSDKNIDEFVA
AtUGT74E2     404 IVRTVGEVM---EGEKGK--EIRKNAEK----WKVLAQEAVS-E-G--GSSDKSINEFVS
AtUGT84B1     405 VERCIEAVT---EGPAAV--DIRRRAAE----LKRVARLALA-P-G--GSSTRNLDLFIS
DgpHBAGT      421 VANCIVEVT---KGPKAE--EFKKNALK----WKKAADDAAR-E-G--GTSYNNLDKFVM
MtUGT84F1     405 LKKCLLEVT---TGEKAE--TLKKNATK----LKKAAEEAVA-V-G--GSSDRHLDAFME
GtUF6CGT1     422 VERALREIT---SGPKAE--EVKENALK----WKKKAEETVA-K-G--GYSERNLVGFIE
PgUGT84A23    417 VEQCLREAT---QGPKAA--EMKKNALK----WKAAAEASFV-E-G--GSSDRNLQAFVD
PgUGT84A24    416 VEKCLLEAT---VGPKAA--EVKENALK----WKAAAEAAVA-E-G--GSSDRNIQAFVD
AtUGT76E1     399 VERAVERLI---MDEEGA--EMRKRVIN----LKEKLQASVKSR----GSSFSSLDNFVN
AtUGT76D1     397 VEMAVRRLI---VDQEGQ--EMRMRATI----LKEEVEASVTTE----GSSHNSLNNLVH
SrUGT76G1     407 IANAIRRVM---VDEEGE--YIRQNARV----LKQKADVSLM-K-G--GSSYESLESLVS
VvGT15        431 IERAVRRLM---VDGEGE--EMRQRAME----LKEKVDICTS-E-G--GSSNRALKELVE
AtUGT76C1     406 IERAVIRLM---VESKGE--EIRGRIKV----LRDEVRRSVK-Q-G--GSSYRSLDELVD
AtUGT76B1     396 IENAVRTLM---TSSEGE--EIRKRIMP----MKETVEQCLK-L-G--GSSFRNLENLIA
CsUGT76F1     398 IEKTIRRVM---VEKQGE--EIRSRIFR----LKEKANHSWK-Q-G--RSSFESINSLVT
OsUGT709A4    424 VARMVREAM------ESG--EIRATAQA----LAEKVRRDVA-D-G--GSSATEFKRLVG
SrUGT85C2     428 VKRLVQELM----GEGGH--KMRNKAKD----WKEKARIAIAPN----GSSSLNIDKMVK
CoUGT85N1     426 IGMMVKELM---KGEKGL--EMRNKVED----LMSKAIKATT-P-G--GSSHTNFEMLME
MeUGT85K4     428 LVSIIKEMM---ETEIGR--ERRRRAVE----WRKKAEEAIS-V-G--GVSYNNFDTFIK
AtUGT85A1     432 VEAVVRELM---DGEKGK--KMREKAVE----WQRLAEKATE---HKLGSSVMNFETVVS
MtUGT85H2     429 LAKLINEVI---AGDKGK--KMKQKAME----LKKKAEENTR-P-G--GCSYMNLNKVIK
MdUGT71K1     427 IEKAVVGVM-----EKDS--EVRKKVEE----MGKMARKAVK-D-G--GSSFASVGRFIE
AtUGT89A2     414 LGRVIAETM----GEGGR--EVAARAEE----IRRKTEAAVT-EAN--GSSVENVQRLVK
AtUGT89C1     387 LARILAESA---REDLPE----RVTLMK----LREKAMEAIK-E-G--GSSYKNLDELVA
AtUGT89B1     420 LARVFADSV---TGNQTE----RIKAVE----LRKAALDAIQER----GSSVNDLDGFIQ
NmUGT89P1     409 LANVLAESI---NGGRAE----RERVTE----LCDAALKAVQ-SGN--GSSAKDLDSLTN
AtUGT92A1     430 IVSKIKLVM--EETEVGK--EIRKKARE----VKELVRRAMV-D-GVKGSSVIGLEEFLD
GbUGT92K1     436 VERIVKMLF-DGHDEKGI--ELRKRAMD----LKAAATIAVSST----GSSITHLDDFIQ
GmUGT92G4     432 VKKTIEIVM-D-YEGKGK--VMKEKANEIAAYIREAKTEKGKEK----GSSVRAMDDLVT
PoUGT90A7     414 LKKMVKELM---EGENGK--EVWKKVKE----VGEAAKVAMA-E-G--GSSWRTLNELID
AtUGT90A1     418 LSGKIKELM---EGETGK--TARKNVKE----YSKMAKAALV-EGT--GSSWKNLDMILK
AcUGT73J1     417 IAGVVGRVM--GGGEKYE--EMKRKAKD----YGEKAKKAVD-E-G--GSSYNDVVALIE
MtUGT73P1     437 IGKAIGLLM-G-GGEECL--EMRKRVKA----LSGAAKKAIE-V-G--GSSYTKLKELIE
SrUGT73E1     438 VKKAVECLM--DEDEDGD--QRRKRVIE----LAKMAKIAMA-E-G--GSSYENVSSLIR
CpPGT4        431 VKTAINILM---DDGEER--NARRSARE----FGELAKRAID-E-G--GSSYNNVELFMQ
AtUGT73C1     434 VKKAVEELM--GDSNDAK--ERRKRVKE----LGELAHKAVE-E-G--GSSHSNITFLLQ
CpPGT2        431 VRNAVEKLM--DEGKEGE--ERRNRAVK----LGQMANMAVQ-E-G--GSSHLNVTLVIQ
PjGAT         439 IQKAIETIM-NGGGEEGG---IRKRATK----LSKVGARAME-D-G--GSSHFNISLLIQ
CtiUGT73AE1   435 VSNAIQKAM--DVGNEGN--ERRRKVKA----LGKVAENAIE-E-G--GSSYKNLTLLIK
CaUGT73AH1    441 VKNAVECIM--SEDEEGE--YRRNRAKK----LAEMAVKAVE-E-G--GSSNLNMTLLIQ
PzGAT2        436 VKEAIECIM--LEGKEGQ--ERRKRARE----LAKEAVRAVE-E-G--GSSHLNITLLIE
AcUGT73G1     421 IETAVKKLM--GDDEEAE--ERRRRAKE----LAAMARKAVE-E-G--GSSYELMSDLIR
MtUGT73K1     423 IEKAVKKLM--DSNGEGG--EIRKRAKE----MKEKAWKAVQ-E-G--GSSQNCLTKLVD
GeUGT73F1     423 IEKAVRRLM--DGGDEAE--KIRRRARE----FRDKATRAVQ-E-G--GSSHNNLTALID
AtUGT73B1     433 VEGAVREVM------VGE--ERRKRAKE----LAEMAKNAVK-E-G--GSSDLEVDRLME
GuUGAT        428 IEQAVRRIM---VGQEAE--EMRNRAKE----LSQMAKRAVE-E-G--GSSHNDFNSLIE
NmUGT73BD1    412 VSDAVKRVM---EGEEAI--EMRTRAKS----LKKMACKAVE-E-G--GSSYNDLTSLID
NmUGT73BD1      1 ------------------------------------MTAQFHVVFFP-LM-AQGHLIP
```

```
AtUGT81A1     522 KK--------NSLPQLSCTA----------------------------------------
AtUGT80A2     636 FS----------------------------------------------------------
AtUGT80B1     599 WF--------FIQIGKKCCLPCGGV-----------------------------------
OsZOGT3       461 HI--------TR------------------------------------------------
OsZOGT1       460 YI--------TR------------------------------------------------
ZmcisZOG1     464 YI--------TR------------------------------------------------
GmUGT79A6     459 QL--------KSMA----------------------------------------------
AtUGT79B6     442 AL--------EKLVQNINLE----------------------------------------
GmUGT91H9     453 AL--------QKYKTPNSNC----------------------------------------
ZmUGT91L1     421 SL--------RTYKN---------------------------------------------
PoUGT95A1     539 FI--------SQKAT---------------------------------------------
MtUGT95B4     480 FI--------KQRFV---------------------------------------------
PgUGT95B2     512 FI--------RQKTK---------------------------------------------
VvUGT95B6     503 FI--------NQRAP---------------------------------------------
AtUGT82A1     459 EL--------N-------------------------------------------------
VpUGT94F1     442 LC--------AKNKLQKCD-----------------------------------------
AtUGT87A1     435 DI--------TKIV----------------------------------------------
AtUGT72C1     454 AY--------FT------------------------------------------------
AtUGT72D1     465 RC--------YLVP----------------------------------------------
LjUGT72AD1    471 KW--------KRLAVEGEGN----------------------------------------
AtUGT72B1     448 KW--------KAHKKELEQNGNH-------------------------------------
VvUGT1        462 IW--------KNIKV---------------------------------------------
LjUGT72AH1    464 KW--------KSLGTITQD-----------------------------------------
LjUGT72Z2     481 QI--------ENFLALK-------------------------------------------
GmUGT72X4     461 QW--------ECFSGNC-------------------------------------------
MtUGT72L1     472 KW--------RNLVQKNQI-----------------------------------------
PgUGT72BD1    476 EC--------KIGLVGLKQRARGA------------------------------------
AtUGT72E1     463 ES--------EHLLERVRCMARGA------------------------------------
OsUGT706C1    457 NL--------GTNNVKSGPRDTPVHD----------------------------------
OsUGT706D1    466 RL--------VTSE----------------------------------------------
MtUGT88E1     460 SW--------REHASWTSVSPNSPFLFA--------------------------------
VpUGT88D8     451 LV--------IG------------------------------------------------
NmUGT88P1     472 GW--------K-------------------------------------------------
AtUGT88A1     458 SW--------SPK-----------------------------------------------
VvGT7         475 SW--------RPG-----------------------------------------------
MdUGT88F1     481 SI--------T-------------------------------------------------
ScUGT5        471 VW--------FGN-----------------------------------------------
OsUGT707A2    483 EM--------AHMSSI--------------------------------------------
AtUGT71B1     467 DV--------VDNVP---------------------------------------------
MdUGT71A15    470 KI----------------------------------------------------------
BvUGT71F1     487 DV--------LTFE----------------------------------------------
Db6-GT        474 DL--------LENAGF--------------------------------------------
AtUGT71C1     472 DL--------IDGVSISK------------------------------------------
AtUGT71C4     471 EL--------FEDGSSC-------------------------------------------
AtUGT71D1     461 DV--------IGIKP---------------------------------------------
FaUGT71W2     467 VL--------SQNLENN-------------------------------------------
MtUGT71G1     460 DI--------TGSN----------------------------------------------
MdUGT71K1     472 DV--------IGQN----------------------------------------------
AtUGT89A2     461 EF--------EKV-----------------------------------------------
AtUGT89C1     432 EM--------CL------------------------------------------------
AtUGT89B1     465 HV--------VSLGLNK-------------------------------------------
NmUGT89P1     455 QL--------NGLKVKIN------------------------------------------
AtUGT92A1     480 QA--------MVKKVEN-------------------------------------------
GbUGT92K1     485 KL--------KYSKGMENQG----------------------------------------
GmUGT92G4     484 TI--------LSPKVL--------------------------------------------
PoUGT90A7     461 EL--------QSVRN---------------------------------------------
AtUGT90A1     466 EL--------CKSRDSNGASE---------------------------------------
AcUGT73J1     465 EL--------KTL-----------------------------------------------
MtUGT73P1     485 EL--------KSFKLEKINKKLVSVT----------------------------------
SrUGT73E1     486 DV--------TETVRAPH------------------------------------------
CpPGT4        478 DI--------MQQPSSEVI-----------------------------------------
AtUGT73C1     482 DI--------MQLEQPKK------------------------------------------
CpPGT2        479 DI--------MKHVHSSSQAN---------------------------------------
PjGAT         487 DI--------WKQKNNQEKL----------------------------------------
CtiUGT73AE1   483 DI--------RELAKDH-------------------------------------------
CaUGT73AH1    489 DI--------IEQVKDRLALS---------------------------------------
PzGAT2        484 DI--------M-------------------------------------------------
AcUGT73G1     469 EL--------EGLRDRRNK-----------------------------------------
MtUGT73K1     471 YL--------HSVVVTKSVELN--------------------------------------
GeUGT73F1     471 DL--------RRLRDRKVLD----------------------------------------
AtUGT73B1     477 EL--------TLVKLQKEKV----------------------------------------
GuUGAT        475 DL--------RSRAH---------------------------------------------
NmUGT73BD1    459 GV--------KAYRSQSSEAEEEHN-----------------------------------
NmUGT73BD1      1 --------------------------------------MTAQFHVVFFP-LM-AQGHLIP
```

```
NtTOGT1      467 DI--------STYSSTGH------------------------------------------
BvUGT73A4    470 EL--------RGLST---------------------------------------------
Db5-GT       479 EL--------KGYHTSEKE-----------------------------------------
AtUGT86A1    469 EV--------RNRIETKLCNVNGLEISPSN------------------------------
ZmUFGT1      467 IV--------CRA-----------------------------------------------
CteUGT78K6   442 VI--------SSS-----------------------------------------------
AtUGT78D1    449 EI--------VKV-----------------------------------------------
CoUGT78B3    450 IV--------TARN----------------------------------------------
MtUGT78G1    451 IV--------TS------------------------------------------------
LgUGT78J1    441 II--------CI------------------------------------------------
CpPGT11      451 SI--------KQWPA---------------------------------------------
AtUGT83A1    458 WI--------KSQVN---------------------------------------------
FaUGT75T1    452 SL--------A-------------------------------------------------
AtUGT75C1    454 ED--------E-------------------------------------------------
AtUGT75B1    450 DICGESLIQNLCEAEEVKVK----------------------------------------
AtUGT75D1    472 EH--------M-------------------------------------------------
ZmUGT74A1    463 FV--------RAGATEK-------------------------------------------
AsUGT74H5    442 AL--------RRSRKSYEAKPIEPLLVGLDA-----------------------------
BdUGT74J7    464 KY--------ASS-----------------------------------------------
SrUGT74G1    456 EL--------IKA-----------------------------------------------
AtUGT74B1    457 SL--------GK------------------------------------------------
RsUGT74R1    448 GL--------ALKD----------------------------------------------
LuUGT74S1    456 SL--------INFAETFQFSC---------------------------------------
AtUGT74F1    445 KI--------QIK-----------------------------------------------
SgUGT74AC1   450 ML--------KQT-----------------------------------------------
AtUGT74C1    455 LL--------T-------------------------------------------------
AtUGT74D1    453 KI--------VR------------------------------------------------
AtUGT74E2    451 MF--------C-------------------------------------------------
AtUGT84B1    452 DI--------TIA-----------------------------------------------
DgpHBAGT     468 EV--------QALKQGLCTSEEDPHQS---------------------------------
MtUGT84F1    452 DI--------KKHKRC--------------------------------------------
GtUF6CGT1    469 EV--------ARKTGTK-------------------------------------------
PgUGT84A23   464 EV--------KRRSIEITASKPAVKAAPNGVVAAAESVVETKANGKVELAA---------
PgUGT84A24   463 EV--------KRRSIAIQSNKSEPKPVVQNAAVADHFGAKATTNGVAADLAGSNADGKVE
AtUGT76E1    446 SL--------KMMNFM--------------------------------------------
AtUGT76D1    444 AI--------MMQIDEQ-------------------------------------------
SrUGT76G1    454 YI--------SSL-----------------------------------------------
VvGT15       478 YI--------SSF-----------------------------------------------
AtUGT76C1    453 RI--------SIIIEPLVPT----------------------------------------
AtUGT76B1    443 YI--------LSF-----------------------------------------------
CsUGT76F1    445 HI--------LSLETFTFHTQ---------------------------------------
OsUGT709A4   468 FL--------QELATRIQHAN---------------------------------------
SrUGT85C2    474 EI--------TVLARN--------------------------------------------
CoUGT85N1    473 DV--------AKW-----------------------------------------------
MeUGT85K4    475 EV--------ILQQQTQ-------------------------------------------
AtUGT85A1    480 KF--------LLGQKSQD------------------------------------------
MtUGT85H2    476 DV--------LLKQN---------------------------------------------
MdUGT71K1    472 DV--------IGQN----------------------------------------------
AtUGT89A2    461 EF--------EKV-----------------------------------------------
AtUGT89C1    432 EM--------CL------------------------------------------------
AtUGT89B1    465 HV--------VSLGLNK-------------------------------------------
NmUGT89P1    455 QL--------NGLKVKIN------------------------------------------
AtUGT92A1    480 QA--------MVKKVEN-------------------------------------------
GbUGT92K1    485 KL--------KYSKGMENQG----------------------------------------
GmUGT92G4    484 TI--------LSPKVL--------------------------------------------
PoUGT90A7    461 EL--------QSVRN---------------------------------------------
AtUGT90A1    466 EL--------CKSRDSNGASE---------------------------------------
AcUGT73J1    465 EL--------KTL-----------------------------------------------
MtUGT73P1    485 EL--------KSFKLEKINKKLVSVT----------------------------------
SrUGT73E1    486 DV--------TETVRAPH------------------------------------------
CpPGT4       478 DI--------MQQPSSEVI-----------------------------------------
AtUGT73C1    482 DI--------MQLEQPKK------------------------------------------
CpPGT2       479 DI--------MKHVHSSSQAN---------------------------------------
PjGAT        487 DI--------WKQKNNQEKL----------------------------------------
CtiUGT73AE1  483 DI--------RELAKDH-------------------------------------------
CaUGT73AH1   489 DI--------IEQVKDRLALS---------------------------------------
PzGAT2       484 DI--------M-------------------------------------------------
AcUGT73G1    469 EL--------EGLRDRRNK-----------------------------------------
MtUGT73K1    471 YL--------HSVVVTKSVELN--------------------------------------
GeUGT73F1    471 DL--------RRLRDRKVLD----------------------------------------
AtUGT73B1    477 EL--------TLVKLQKEKV----------------------------------------
GuUGAT       475 DL--------RSRAH---------------------------------------------
NmUGT73BD1   459 GV--------KAYRSQSSEAEEEHN-----------------------------------
NmUGT73BD1     1 --------------------------------------MTAQFHVVFFP-LM-AQGHLIP
```

```
AtUGT81A1             ----------STYSSTGH------------------------------------------
AtUGT80A2             ----------RGLST---------------------------------------------
AtUGT80B1             ----------KGYHTSEKE-----------------------------------------
OsZOGT3               ----------RNRIETKLCNVNGLEISPSN------------------------------
OsZOGT1               ----------CRA-----------------------------------------------
ZmcisZOG1             ----------SSS-----------------------------------------------
GmUGT79A6             ----------VKV-----------------------------------------------
AtUGT79B6             ----------TARN----------------------------------------------
GmUGT91H9             ----------TS------------------------------------------------
ZmUGT91L1             ----------CI------------------------------------------------
PoUGT95A1             ----------KQWPA---------------------------------------------
MtUGT95B4             ----------KSQVN---------------------------------------------
PgUGT95B2             ----------A-------------------------------------------------
VvUGT95B6             ----------E-------------------------------------------------
AtUGT82A1             ---GESLIQNLCEAEEVKVK----------------------------------------
VpUGT94F1             ----------M-------------------------------------------------
AtUGT87A1             ----------RAGATEK-------------------------------------------
AtUGT72C1             ----------RRSRKSYEAKPIEPLLVGLDA-----------------------------
AtUGT72D1             ----------ASS-----------------------------------------------
LjUGT72AD1            ----------IKA-----------------------------------------------
AtUGT72B1             ----------GK------------------------------------------------
VvUGT1                ----------ALKD----------------------------------------------
LjUGT72AH1            ----------INFAETFQFSC---------------------------------------
LjUGT72Z2             ----------QIK-----------------------------------------------
GmUGT72X4             ----------KQT-----------------------------------------------
MtUGT72L1             ----------T-------------------------------------------------
PgUGT72BD1            ----------VR------------------------------------------------
AtUGT72E1             ----------C-------------------------------------------------
OsUGT706C1            ----------TIA-----------------------------------------------
OsUGT706D1            ----------QALKQGLCTSEEDPHQS---------------------------------
MtUGT88E1             ----------KKHKRC--------------------------------------------
VpUGT88D8             ----------ARKTGTK-------------------------------------------
NmUGT88P1             ----------KRRSIEITASKPAVKAAPNGVVAAAESVVETKANGKVELAA---------
AtUGT88A1             ----------KRRSIAIQSNKSEPKPVVQNAAVADHFGAKATTNGVAADLAGSNADGKVE
VvGT7                 ----------KMMNFM--------------------------------------------
MdUGT88F1             ----------MMQIDEQ-------------------------------------------
ScUGT5                ----------SSL-----------------------------------------------
OsUGT707A2            ----------SSF-----------------------------------------------
AtUGT71B1             ----------SIIIEPLVPT----------------------------------------
MdUGT71A15            ----------LSF-----------------------------------------------
BvUGT71F1             ----------LSLETFTFHTQ---------------------------------------
Db6-GT                ----------QELATRIQHAN---------------------------------------
AtUGT71C1             ----------TVLARN--------------------------------------------
AtUGT71C4             ----------AKW-----------------------------------------------
AtUGT71D1             ----------ILQQQTQ-------------------------------------------
FaUGT71W2             ----------LLGQKSQD------------------------------------------
MtUGT71G1             ----------LLKQN---------------------------------------------
MdUGT71K1             ----------IGQN----------------------------------------------
AtUGT89A2             ----------EKV-----------------------------------------------
AtUGT89C1             ----------CL------------------------------------------------
AtUGT89B1             ----------VSLGLNK-------------------------------------------
NmUGT89P1             ----------NGLKVKIN------------------------------------------
AtUGT92A1             ----------MVKKVEN-------------------------------------------
GbUGT92K1             ----------KYSKGMENQG----------------------------------------
GmUGT92G4             ----------LSPKVL--------------------------------------------
PoUGT90A7             ----------QSVRN---------------------------------------------
AtUGT90A1             ----------CKSRDSNGASE---------------------------------------
AcUGT73J1             ----------KTL-----------------------------------------------
MtUGT73P1             ----------KSFKLEKINKKLVSVT----------------------------------
SrUGT73E1             ----------TETVRAPH------------------------------------------
CpPGT4                ----------MQQPSSEVI-----------------------------------------
AtUGT73C1             ----------MQLEQPKK------------------------------------------
CpPGT2                ----------MKHVHSSSQAN---------------------------------------
PjGAT                 ----------WKQKNNQEKL----------------------------------------
CtiUGT73AE1           ----------RELAKDH-------------------------------------------
CaUGT73AH1            ----------IEQVKDRLALS---------------------------------------
PzGAT2                ----------M-------------------------------------------------
AcUGT73G1             ----------EGLRDRRNK-----------------------------------------
MtUGT73K1             ----------HSVVVTKSVELN--------------------------------------
GeUGT73F1             ----------RRLRDRKVLD----------------------------------------
AtUGT73B1             ----------TLVKLQKEKV----------------------------------------
GuUGAT                ----------RSRAH---------------------------------------------
NmUGT73BD1            ----------KAYRSQSSEAEEEHN-----------------------------------
NmUGT73BD1         1  --------------------------------------MTAQFHVVFFP-LM-AQGHLIP
```

```
NtTOGT1                  ----------STYSSTGH------------------------------------------
BvUGT73A4                ----------RGLST---------------------------------------------
Db5-GT                   ----------KGYHTSEKE-----------------------------------------
AtUGT86A1                ----------RNRIETKLCNVNGLEISPSN------------------------------
ZmUFGT1                  ----------CRA-----------------------------------------------
CteUGT78K6               ----------SSS-----------------------------------------------
AtUGT78D1                ----------VKV-----------------------------------------------
CoUGT78B3                ----------TARN----------------------------------------------
MtUGT78G1                ----------TS------------------------------------------------
LgUGT78J1                ----------CI------------------------------------------------
CpPGT11                  ----------KQWPA---------------------------------------------
AtUGT83A1                ----------KSQVN---------------------------------------------
FaUGT75T1                ----------A-------------------------------------------------
AtUGT75C1                ----------E-------------------------------------------------
AtUGT75B1                ---GESLIQNLCEAEEVKVK----------------------------------------
AtUGT75D1                ----------M-------------------------------------------------
ZmUGT74A1                ----------RAGATEK-------------------------------------------
AsUGT74H5                ----------RRSRKSYEAKPIEPLLVGLDA-----------------------------
BdUGT74J7                ----------ASS-----------------------------------------------
SrUGT74G1                ----------IKA-----------------------------------------------
AtUGT74B1                ----------GK------------------------------------------------
RsUGT74R1                ----------ALKD----------------------------------------------
LuUGT74S1                ----------INFAETFQFSC---------------------------------------
AtUGT74F1                ----------QIK-----------------------------------------------
SgUGT74AC1               ----------KQT-----------------------------------------------
AtUGT74C1                ----------T-------------------------------------------------
AtUGT74D1                ----------VR------------------------------------------------
AtUGT74E2                ----------C-------------------------------------------------
AtUGT84B1                ----------TIA-----------------------------------------------
DgpHBAGT                 ----------QALKQGLCTSEEDPHQS---------------------------------
MtUGT84F1                ----------KKHKRC--------------------------------------------
GtUF6CGT1                ----------ARKTGTK-------------------------------------------
PgUGT84A23               ----------KRRSIEITASKPAVKAAPNGVVAAAESVVETKANGKVELAA---------
PgUGT84A24           515 LVA-------KRRSIAIQSNKSEPKPVVQNAAVADHFGAKATTNGVAADLAGSNADGKVE
AtUGT76E1                ----------KMMNFM--------------------------------------------
AtUGT76D1                ----------MMQIDEQ-------------------------------------------
SrUGT76G1                ----------SSL-----------------------------------------------
VvGT15                   ----------SSF-----------------------------------------------
AtUGT76C1                ----------SIIIEPLVPT----------------------------------------
AtUGT76B1                ----------LSF-----------------------------------------------
CsUGT76F1                ----------LSLETFTFHTQ---------------------------------------
OsUGT709A4               ----------QELATRIQHAN---------------------------------------
SrUGT85C2                ----------TVLARN--------------------------------------------
CoUGT85N1                ----------AKW-----------------------------------------------
MeUGT85K4                ----------ILQQQTQ-------------------------------------------
AtUGT85A1                ----------LLGQKSQD------------------------------------------
MtUGT85H2                ----------LLKQN---------------------------------------------
MdUGT71K1                ----------IGQN----------------------------------------------
AtUGT89A2                ----------EKV-----------------------------------------------
AtUGT89C1                ----------CL------------------------------------------------
AtUGT89B1                ----------VSLGLNK-------------------------------------------
NmUGT89P1                ----------NGLKVKIN------------------------------------------
AtUGT92A1                ----------MVKKVEN-------------------------------------------
GbUGT92K1                ----------KYSKGMENQG----------------------------------------
GmUGT92G4                ----------LSPKVL--------------------------------------------
PoUGT90A7                ----------QSVRN---------------------------------------------
AtUGT90A1                ----------CKSRDSNGASE---------------------------------------
AcUGT73J1                ----------KTL-----------------------------------------------
MtUGT73P1                ----------KSFKLEKINKKLVSVT----------------------------------
SrUGT73E1                ----------TETVRAPH------------------------------------------
CpPGT4                   ----------MQQPSSEVI-----------------------------------------
AtUGT73C1                ----------MQLEQPKK------------------------------------------
CpPGT2                   ----------MKHVHSSSQAN---------------------------------------
PjGAT                    ----------WKQKNNQEKL----------------------------------------
CtiUGT73AE1              ----------RELAKDH-------------------------------------------
CaUGT73AH1               ----------IEQVKDRLALS---------------------------------------
PzGAT2                   ----------M-------------------------------------------------
AcUGT73G1                ----------EGLRDRRNK-----------------------------------------
MtUGT73K1                ----------HSVVVTKSVELN--------------------------------------
GeUGT73F1                ----------RRLRDRKVLD----------------------------------------
AtUGT73B1                ----------TLVKLQKEKV----------------------------------------
GuUGAT                   ----------RSRAH---------------------------------------------
NmUGT73BD1               ----------KAYRSQSSEAEEEHN-----------------------------------
NmUGT73BD1             1 --------------------------------------MTAQFHVVFFP-LM-AQGHLIP
```

```
NtTOGT1            ----------STYSSTGH------------------------------------------
BvUGT73A4          ----------RGLST---------------------------------------------
Db5-GT             ----------KGYHTSEKE-----------------------------------------
AtUGT86A1          ----------RNRIETKLCNVNGLEISPSN------------------------------
ZmUFGT1            ----------CRA-----------------------------------------------
CteUGT78K6         ----------SSS-----------------------------------------------
AtUGT78D1          ----------VKV-----------------------------------------------
CoUGT78B3          ----------TARN----------------------------------------------
MtUGT78G1          ----------TS------------------------------------------------
LgUGT78J1          ----------CI------------------------------------------------
CpPGT11            ----------KQWPA---------------------------------------------
AtUGT83A1          ----------KSQVN---------------------------------------------
FaUGT75T1          ----------A-------------------------------------------------
AtUGT75C1          ----------E-------------------------------------------------
AtUGT75B1          ---GESLIQNLCEAEEVKVK----------------------------------------
AtUGT75D1          ----------M-------------------------------------------------
ZmUGT74A1          ----------RAGATEK-------------------------------------------
AsUGT74H5          ----------RRSRKSYEAKPIEPLLVGLDA-----------------------------
BdUGT74J7          ----------ASS-----------------------------------------------
SrUGT74G1          ----------IKA-----------------------------------------------
AtUGT74B1          ----------GK------------------------------------------------
RsUGT74R1          ----------ALKD----------------------------------------------
LuUGT74S1          ----------INFAETFQFSC---------------------------------------
AtUGT74F1          ----------QIK-----------------------------------------------
SgUGT74AC1         ----------KQT-----------------------------------------------
AtUGT74C1          ----------T-------------------------------------------------
AtUGT74D1          ----------VR------------------------------------------------
AtUGT74E2          ----------C-------------------------------------------------
AtUGT84B1          ----------TIA-----------------------------------------------
DgpHBAGT           ----------QALKQGLCTSEEDPHQS---------------------------------
MtUGT84F1          ----------KKHKRC--------------------------------------------
GtUF6CGT1          ----------ARKTGTK-------------------------------------------
PgUGT84A23         ----------KRRSIEITASKPAVKAAPNGVVAAAESVVETKANGKVELAA---------
PgUGT84A24     515 LVA-------KRRSIAIQSNKSEPKPVVQNAAVADHFGAKATTNGVAADLAGSNADGKVE
AtUGT76E1          ----------KMMNFM--------------------------------------------
AtUGT76D1          ----------MMQIDEQ-------------------------------------------
SrUGT76G1          ----------SSL-----------------------------------------------
VvGT15             ----------SSF-----------------------------------------------
AtUGT76C1          ----------SIIIEPLVPT----------------------------------------
AtUGT76B1          ----------LSF-----------------------------------------------
CsUGT76F1          ----------LSLETFTFHTQ---------------------------------------
OsUGT709A4         ----------QELATRIQHAN---------------------------------------
SrUGT85C2          ----------TVLARN--------------------------------------------
CoUGT85N1          ----------AKW-----------------------------------------------
MeUGT85K4          ----------ILQQQTQ-------------------------------------------
AtUGT85A1          ----------LLGQKSQD------------------------------------------
MtUGT85H2          ----------LLKQN---------------------------------------------
MdUGT71K1          ----------IGQN----------------------------------------------
AtUGT89A2          ----------EKV-----------------------------------------------
AtUGT89C1          ----------CL------------------------------------------------
AtUGT89B1          ----------VSLGLNK-------------------------------------------
NmUGT89P1          ----------NGLKVKIN------------------------------------------
AtUGT92A1          ----------MVKKVEN-------------------------------------------
GbUGT92K1          ----------KYSKGMENQG----------------------------------------
GmUGT92G4          ----------LSPKVL--------------------------------------------
PoUGT90A7          ----------QSVRN---------------------------------------------
AtUGT90A1          ----------CKSRDSNGASE---------------------------------------
AcUGT73J1          ----------KTL-----------------------------------------------
MtUGT73P1          ----------KSFKLEKINKKLVSVT----------------------------------
SrUGT73E1          ----------TETVRAPH------------------------------------------
CpPGT4             ----------MQQPSSEVI-----------------------------------------
AtUGT73C1          ----------MQLEQPKK------------------------------------------
CpPGT2             ----------MKHVHSSSQAN---------------------------------------
PjGAT              ----------WKQKNNQEKL----------------------------------------
CtiUGT73AE1        ----------RELAKDH-------------------------------------------
CaUGT73AH1         ----------IEQVKDRLALS---------------------------------------
PzGAT2             ----------M-------------------------------------------------
AcUGT73G1          ----------EGLRDRRNK-----------------------------------------
MtUGT73K1          ----------HSVVVTKSVELN--------------------------------------
GeUGT73F1          ----------RRLRDRKVLD----------------------------------------
AtUGT73B1          ----------TLVKLQKEKV----------------------------------------
GuUGAT             ----------RSRAH---------------------------------------------
NmUGT73BD1         ----------KAYRSQSSEAEEEHN-----------------------------------
NmUGT73BD1       1 --------------------------------------MTAQFHVVFFP-LM-AQGHLIP
```
